# Supplementary material for: A ribosomally synthesised and post-translationally modified peptide containing a β-enamino acid and a macrocyclic motif
Source: Nat Commun. 2022 Aug 26;13:5044. doi: 10.1038/s41467-022-32774-3 (PMC9415263; doi:10.1038/s41467-022-32774-3)
Supplement: Supplementary file 1 — Supplementary information [file 41467_2022_32774_MOESM1_ESM.pdf]

Supplementary Information

A ribosomally synthesized and post-translationally modified peptide containing a β-enamino acid and a macrocyclic motif

Shan Wang[1#], Sixing Lin[2#], Qing Fang[1], Roland Gyampoh[3], Zhou Lu[1], Yingli Gao[1,4], David J. Clarke[5], Kewen Wu[1], Laurent Trembleau[1], Yi Yu*[2], Kwaku Kyeremeh*[3], Bruce F. Milne*[1,6], Jioji Tabudravu*[7], Hai Deng*[1]

[1] Department of Chemistry, University of Aberdeen, Aberdeen AB24 3UE, UK

[2] Key Laboratory of Combinatorial Biosynthesis and Drug Discovery (MOE) and Hubei Province Engineering and Technology Research Centre for Fluorinated Pharmaceuticals, School of Pharmaceutical Sciences, Wuhan University, Wuhan 430071, China

[3] Department of Chemistry, University of Ghana, P.O. Box LG56, Legon-Accra, Ghana

[4] College of Marine Life and Fisheries, Jiangsu Ocean University, Lianyungang, Jiangsu Province, China

[5] EastChem, School of Chemistry, University of Edinburgh, Edinburgh, EH9 3FJ, UK

[6] CFisUC, Department of Physics, University of Coimbra, Rua Larga, 3004-516, Coimbra, Portugal

[7] School of Forensic & Applied Sciences, Faculty of Science & Technology, University of Central Lancashire, Preston, Lancashire, PR1 2HE, England, UK

Corresponding authors:

Dr Hai Deng, h.deng@abdn.ac.uk

Dr Jioji Tabudravu, JTabudravu@uclan.ac.uk

Dr Bruce F. Milne, bfmilne@uc.pt

Professor Kwaku Kyeremeh, kkyeremeh@ug.edu.gh

Professor Yi Yu, yu_yi@whu.edu.cn

[#] These authors contributed equally to this work

Supplementary Methods 6
1. Molecular network methods 6
2. Isolation and structure determination of **7** 6
3. Ab-initio molecular dynamics (AIMD) simulation of kintamdin **7** 6
4. Simulation results 7
5. Antiproliferative/Cytotoxicity Assays 8
6. Antibacterial assays 8
7. Heterologous expression of the *kin* BGC 8
8. PCR targeting of the *orf*s in the *kin* BGC 9
9. Purification of the recombinant KinI and KinO in *E. coli* 9
10. Biochemical assay for methyltransferase KinO 10
11. Cultivation of *Streptomyces kurssanovii* 10

Supplementary tables 11
Supplementary Table 1. Fragmentation ions of **7** observed in HR-MS analysis. 11
Supplementary Table 2. Chemical shifts of NMR analysis of **7**. 12
Supplementary Table 3. The retention time comparison of derivatized standard amino acids and amino acid residues from kintamdin **7** after acidic hydrolysis. 16
Supplementary Table 4. Computational calculation of distances of key correlations in NOE among eight diastereoisomers in **7**. 17
Supplementary Table 5. Summary of distance monitor satisfaction in the MD simulations of the kintamdin peptide stereoisomers. 32
Supplementary Table 6. Bioassay activity evaluation of kintamdin **7**. 33
Supplementary Table 7. Deduced Functions of *orfs* in the kintamdin biosynthetic gene cluster (accession number: MW923391). 34
Supplementary Table 8 sequence analysis of key ORFs, KinC, D, H and I with the corresponding enzymes in TVA pathway. 36
Supplementary Table 9. Plasmids used in this study. 37
Supplementary Table 10. Strains used in this study. 38
Supplementary Table 11. Primers used in this study. 40
Supplementary Table 12. KinA homologs identified by BlastP. 44

Supplementary figures 45
Supplementary Fig. 1. High Resolution ESI FT-ICR Mass Spectrometry analysis of **7.** 45
Supplementary Fig. 2. ^{1}H NMR of **7** in CD_3OD at 600 MHz. 46
Supplementary Fig. 3. ^{13}C NMR spectrum for **7** in CD_3OD at 150 MHz. 47
Supplementary Fig. 4. COSY spectrum of **7**. 48
Supplementary Fig. 5. HMBC spectrum of **7**. 49
Supplementary Fig. 6. HSQC-TOCSY of **7**. 50
Supplementary Fig. 7. HSQC spectrum of **7**. 51
Supplementary Fig. 8. Substructure of **7** containing multiple (*Z*)-Dhb motif. 52

Supplementary Fig. 9. Key ROESY correlations indicating *Z* configurations of Dhbs 2, 3, 4, and 6. 53

Supplementary Fig. 10. Key correlations in the ROESY spectra in support of Supplementary Figure 3. 54

Supplementary Fig. 11. Three key substructures based on detail analysis of Hα-NH (i, i+1) ROESY correlations and NH to -CO, Hα to -CO HMBC cross peaks between adjacent amino acids allowed partial determination of peptide sequence. 55

Supplementary Fig. 12. HSQC chemical shifts for Aaa-7. Inset, ^{1}H splitting of 8.4 Hz indicating *cis*-geometry for Aaa-7. 56

Supplementary Fig. 13. Key COSY correlation within Aaa-7 subunit. 57

Supplementary Fig. 14. Key HMBC Correlation within Aaa-7. 58

Supplementary Fig. 15. High Resolution *De Novo* Tandem MS analysis of kintamdin **7.** 59

Supplementary Fig. 16. The putative BGC responsible for the kintamdin production in *Streptomyces* sp. RK44. 60

Supplementary Fig. 17. Expanded and annotated ^{1}H NMR spectrum of **7**. 61

Supplementary Fig. 18. Expanded and annotated ^{1}H NMR spectrum of **7**. 62

Supplementary Fig. 19. Expanded and annotated ^{1}H NMR spectrum of **7**. 63

Supplementary Fig. 20. Expanded and annotated ^{1}H NMR spectrum of **7**. 64

Supplementary Fig. 21. Expanded and annotated ^{1}H NMR spectrum of **7**. 65

Supplementary Fig. 22. Expanded and annotated ^{1}H NMR spectrum of **7**. 66

Supplementary Fig. 23. Expanded and annotated ^{1}H NMR spectrum of **7**. 67

Supplementary Fig. 24. Expanded and annotated ^{1}H NMR spectrum of **7**. 68

Supplementary Fig. 25. Expanded and annotated HSQC spectrum of **7**. 69

Supplementary Fig. 26. Expanded and annotated HSQC spectrum of **7**. 70

Supplementary Fig. 27. Annotated HSQC spectrum of **7**. 71

Supplementary Fig. 28. Key cross-peaks in the COSY, HMBC, and ROESY spectra of the *bis*-thioethane unit of **7**, indicating an unprecendented *bis*-thioether cross link has been found to be present in **7**. 72

Supplementary Fig. 29. Assignment of NHs in key amino acid residues using COSY correlations between α to NH of amino acid residue. 73

Supplementary Fig. 30. Key correlations in the ROESY spectrum showing link between Ala(S1)-11 and aminoethane-1,2-dithiol group. 74

Supplementary Fig. 31. The planar structure of **7**. 75

Supplementary Fig. 32. **a**. EIC chromatography of proteinogenic amino acids residues from kintamdin from advanced Marfey's derivatization. 76

Supplementary Fig. 33. Extracted Ion Chromatogram (EIC) chromatography of derivatized Ala residues from **7** (top), standard L-Ala (middle) and standard D-Ala. 77

Supplementary Fig. 34. Observed ROE correlation between Aaa-7-2 (αH) and the methyl group of Abu(S_2)-22-4. 78

Supplementary Fig. 35. Observed ROE correlation between Ala-21-2 (αH) and Asp-24-3A (βH). 79

Supplementary Fig. 36. Observed ROE correlation between Aminoethane-1,2-dithiol(*S*1, *S*2)-27-1 (αH) and Leu-14-3A (βH). 80

Supplementary Fig. 37. Snapshots of **7** from AIMD simulation. 81

Supplementary Fig. 38. Scheme of a modified TAR cloning strategy by inserting two arms into the original pCAP03-*acc(3)IV* plasmid to generate pCAP03-*kin*LR construct. 82

Supplementary Fig. 39. MS analysis of **7** identified from heterologous expression system. 83

Supplementary Fig. 40. MS^2 fragmentation analysis of **7** identified in the heterologous expression system. 84

Supplementary Fig. 41. EIC extraction of **7** in the variants of the heterologous expression systems. 85

Supplementary Fig 42. Sequence comparison of KinD, KinH, LxmX and Cao14 with two kinase homologues, $TvaC_{S\text{-}87}$ (active kinase) and $TvaE_{S\text{-}87}$ (inactive kinase), in the TVA pathway, suggesting that, while KinD is likely to be an active kinase, KinH is an inactive kinase homologue. 86

Supplementary Fig 43. Sequence similarity network (SSN) using the tools of the Enzyme Function Initiative (EFI). 87

Supplementary Fig. 44. MS analysis of the intermediate **8** accumulated in the *kin*O variant. 88

Supplementary Fig. 45. MS analysis of the intermediate **9** accumulated in the *kinI* variant. 89

Supplementary Fig. 46. SDS page analysis of recombinant KinI (Right) and KinO (Left) with the corrected molecular weights, respectively. 90

Supplementary Fig. 47. EIC analysis of the product generated in the assay of incubation of KinO with **7** accumulated. 91

Supplementary Fig. 48. MS^2 fragmentation analysis of the first peptidyl fragments from the Gluc-treated linear peptide from the co-expression of *kin*AD in *E. coli*. 92

Supplementary Fig. 49. MS and MS^2 fragmentation analysis of the unmodified peptidyl fragment from the co-expression of *kin*AD in *E. coli* after Gluc-treatment. 93

Supplementary Fig. 50. MS^2 fragmentation analysis of the first peptidyl fragments from the Gluc-treated linear peptide from the co-expression of *kin*ACD in *E. coli*. 94

Supplementary Fig. 51. MS and MS^2 fragmentation analysis of the modified CP from the co-expression of *kin*ACDH in *E. coli* after Gluc-treatment. 95

Supplementary Fig. 52. MS and MS^2 fragmentation analysis of the modified CP from the co-expression of *kin*ACDI in *E. coli* after Gluc-treatment. 96

Supplementary Fig. 53. MS^2 fragmentation analysis of the modified CP from the co-expression of *kin*ACDHI in *E. coli* after Gluc-treatment. 97

Supplementary Fig. 54. Peptides containing dehydroamino acid residues tend to be 2.0_5-helix (left) [15, 16] where some peptides containing normal α-amino acid form 3_{10}-helix (right). 98

Supplementary Fig. 55. MS and MS^2 fragmentation analysis of the modified CP from the co-expression of *kin*A_{T2A}CD in *E. coli* after Gluc-treatment. 99

Supplementary Fig. 56. MS and MS^2 fragmentation analysis of the modified CP from the co-expression of *kin*A_{T3A}CD in *E. coli* after Gluc-treatment. 100

Supplementary Fig. 57. MS and MS^2 fragmentation analysis of the modified CP from the co-expression of *kin*A_{T4A}CD in *E. coli* after Gluc-treatment. 101

Supplementary Fig. 58. MS and MS^2 fragmentation analysis of the modified CP from the co-expression of *kin*A_{T6A}CD in *E. coli* after Gluc-treatment. 102

Supplementary Fig. 59. MS and MS^2 fragmentation analysis of the modified CP from the co-expression of *kin*A_{S8A}CD in *E. coli* after Gluc-treatment. 103

Supplementary Fig. 60. MS comparison of ion intensities of the fully dehydrated and partially dehydrated linear peptides from the co-expression of *kin*A_{TtoA}CD in *E. coli* after Gluc-treatment. 104

Supplementary Fig. 61. MS and MS^2 fragmentation analysis of the fully dehydrated CP from the co-expression of *kin*A_{TtoA}CD in *E. coli* after Gluc-treatment. 105

Supplementary Fig. 62. MS and MS^2 fragmentation analysis of the partially dehydrated CP from the co-expression of *kin*A_{TtoA}CD in *E. coli* after Gluc-treatment. 106

Supplementary Fig. 63. MS and MS^2 fragmentation analysis of the fully dehydrated mutated CP from the co-expression of *kin*A_{S7T}CD in *E. coli* after Gluc-treatment. 107

Supplementary Fig. 64. MS analysis of trace amount of two dehydrated peptidyl fragments from the co-expression of *kin*A_{C11A}CDHI in *E. coli* after Gluc-treatment. 108

Supplementary Fig. 65. The representative KinA-like peptide sequences identified from conserved genome context 109

Supplementary Fig. 66. Representative BGCs containing *kinA*-like precursor peptides identified by NCBI BlastP listed in **Supplementary Table S12**. 110

Supplementary Fig. 67. The BGC responsible for new putative β-bithionin RiPPs in *Streptomyces kurssanovii* NCIMB 12788 111

Supplementary Fig. 68. Identification of one of the new β-bithionin RiPPs from the culture broth of *Streptomyces kurssanovii* NCIMB 12788 from conserved genomic analysis and experimental evidence. 112

Supplementary Fig. 69. Identification of the second new β-bithionin RiPP from the culture broth of *Streptomyces kurssanovii* NCIMB 12788 from conserved genomic analysis and experimental evidence. 113

Supplementary Fig. 70. Proposed mechanisms of activation and aziridination on Thr residues in chemical and biochemical reactions. 114

Supplementary References 115

Supplementary Methods

1. Molecular network methods

HRMS raw data were analysed by Thermo Xcalibur (version 3.1) and further processed with ProteoWizard MSconvert software and MZmine 2.38. MS/MS metabolite identifications were made by comparing experimental MS/MS spectra with library spectra from GNPS[1] (Global Natural Products Social Molecular Networking) library. For the metabolites that were selected for more in-depth characterization, classification of structure or substructure was performed by searching databases such as Antibase 2012, NP Atlas, Dictionary of Natural Products and GNPS. Predicted structures resulting from a matched intact mass (Δ4 ppm error) were subsequently validated through manual analysis of fragmentation mass spectra.

2. Isolation and structure determination of **7**

Streptomyces sp. RK44 was cultivated in ISP2 medium (10 L) at 28 °C for 7 days. Subsequently, Diaion® HP-20 (3.0 g/50 mL) was added to the culture broth and incubated overnight under the same culture conditions. The mixture was filtered under vacuum, after which the residue consisting of the mycelium and HP-20 resin was submerged in 100% methanol (3 x 500mL) for 24h. The methanol extract was concentrated under reduced pressure to give a crude extract (10.0 g) which was then fractionated by solid phase extraction (SPE) using Strata® C18-E resulting six fractions (S1–S6) and analysed by HRMS. Fraction S2 contained **7** was further purified using High Pressure Liquid Chromatography (HPLC, Agilent Technologies 1260 Infinity, Waldbronn, Germany) over an ACE reversed-phase column(C-18 ,10 μM 10 × 250 mm), and eluting with the following gradient method: (mobile phase A: water +0.1% formic acid; mobile phase B: Methanol) 0–20 min 50% B; 20–20.1 min 50–100% B; 20.1–28 min 100% B; 28–28.1 min 100–40% B; and 28.1–33 min 40% B; flow rate 1.5 mL min^{-1}; injection volume 0.02 mL. Absorbance was monitored at 220 nm. HPLC data were processed using Agilent ChemStation v 17. The pure compound was subjected to analysis by HRMS and 1D and 2D NMR as described in the main text.

For advanced Marfey's analysis, the purified **7** (0.5 mg) was hydrolyzed in 1 mL HCl (6 M) at 115 °C for 17 h. The hydrolysate was evaporated to dryness under a stream of dry N_2 and the residue was dissolved in 100 µL of water and treated with 20 µL $NaHCO_3$(1 M) and 100 µL 1-fluoro-2, 4-dinitrophenyl-5-L-leucinamide (L-FDLA) (1 M) at 40 °C for 1 h. The reaction was quenched with 20 µL of HCl (2 M) and diluted with 200 µL of MeOH. The standard amino acids were derivatized with L-FDLA and analyzed by HPLC-DAD in the same manner as **7**. The stereochemistry was determined by comparing the retention time of the L-FDLA derivatized samples using LC-HRMS analysis.

3. Ab-initio molecular dynamics (AIMD) simulation of kintamdin **7**

Initial structures for the kintamdin peptide **7** were constructed with standard amino acids using the Avogadro software 1.2.0 [3]. Amino acid modifications and cyclisations were then performed by hand, hydrogens were added to correspond to expected ionisation states at pH 7.4 and the resulting structures energy minimized using the MMFF94 molecular mechanics force field. Each of the stereoisomers corresponding to different combinations of chiral centres in the *bis*-thioether bridge were created at this stage and minimised in the same way to produce starting structures for further analysis. The chiral centre at the methyl group in the *bis*-thioether bridge (attached to β-carbon of the Abu(S_2)-22 residue) was kept fixed in the *S*-configuration based on biosynthetic precedent.

The resulting structures were then optimised at the density functional tight-binding level of theory with the xTB software 6.3.0 using the GFN2-xTB electronic structure method which incorporates the accurate D4 correction for modelling dispersion effects [4,5]. Whilst more computationally demanding than classical force-field approaches, this method was chosen because of the large number of non-standard features in **7** which meant that conventional force-field approaches were likely to be

unreliable due to the lack of accurate force-field parameters. Solvent (methanol) effects were included through a GB/SA continuum treatment.

The GFN2-xTB optimised structures were then used as starting points for NVT ensemble molecular dynamics simulations which were performed at 298K, again including methanol solvent effects. All bonds were constrained using the SHAKE algorithm which permitted the use of a 4 fs time step in the simulations [6,7]. A total of 300 ps of simulation was performed for each of the eight possible *bis*-thioether bridge stereoisomers. The initial structures in the MD simulations were seen to undergo large changes in the conformation of the acyclic peptide chain (residues 1 – 10) which folded rapidly within the first 50 to 100 ps and thereafter remained relatively fixed. The 100 – 200 ps period was treated as further equilibration and then in the final 100 ps of the simulations distance monitors corresponding to the NMR coupling data from the NOESY experiments were used to collect data to be used for the evaluation of the closeness of fit between the different structural models and the experimental observations. The full distance monitor data is provided in Supplementary Table 4.

In the candidate isomers, short- and medium- range monitors provided little useful guidance to help differentiate between possible stereoisomers. This is not completely unexpected since distances of the (next-) nearest-neighbour kind are less likely to display significant changes upon stereochemical inversion in positions that are a short distance away (Supplementary Table 4). However, NOE signals corresponding to interactions between protons at large separations in the primary structure were a much more sensitive indicator of higher-order geometrical features since only structures capable of folding in the correct way could give rise to these signals in the experiment.

The 3-D structure of **7** was visualized in UCSF Chimera 1.15. It contains a fused bicyclic structure rigidified by the two crosslinks as well as important backbone and side chain hydrogen bonds (Supplementary Fig. 37). Extensive hydrogen bonds among the backbone of amino acids residues (such as carbonyl at DAla-13 with NH at Val-15, carbonyl at Leu-14 with NHs of Ala-17 and DAla-18, respectively, carbonyl of Ala-17 with Dhb-20, carbonyl of DAla-18 with NH of MAbi residue) within the fused macrocycle were also observed, giving the ring system a cage-like secondary structure (Supplementary Fig. 37). Interestingly, the N-terminal tail is also structured through hydrogen bonds among the side chain of Glu-9, the NH and carbonyl groups in the backbone, making a helix-like N-terminus coil toward the fused macrocyclic cage (Supplementary Fig. 37). The *Z*-configuration at Aaa-7, possessing an N-H••O=C intra-residue H-bond within an average distance of 2.14±0.20 Å, makes an interesting kink formation at the N-terminal chain (Supplementary Fig. 37), a similar phenomenon observed in the synthetic peptidyl foldamer nanomaterials containing Aaa residues [21-22]. Together, the study above provides a conformational model for 7 and indicate that the crosslinks are generated with the *R*, *S*, *S, S* configurations. The cage-like macrocycle C-terminus as well as the helix-like *N*-terminus are significantly stabilised via hydrogen bonding, a prediction that can be tested experimentally in the future.

4. Simulation results

Supplementary Table 5 shows a summary of the results of the distance monitors used in the kintamdin peptide molecular dynamics simulations used to check agreement with the interactions observed in the ROESY experiments. Looking at the total figures it is difficult to unequivocally decide between the candidate structures other than to exclude the 11*R*, 22α*R*, 22β*S*, 27*R* stereoisomer which satisfied approximately 10% less of the monitors than the other structures. An almost identical picture is obtained from inspection of the short distance monitors (to be expected since these are by far the most numerous of the three types). Similarly, the medium-range interactions did not provide particularly useful information to aid in distinguishing between the structures, leading to the conclusion that both short- and medium-range interactions were best classed as 'local' correlations which would be expected to provide similar NMR signals regardless of the exact nature of the stereocentres under study.

Thus, it was concluded that the key information would be most likely to be whether or not an individual structure was capable of satisfying the longer-range distance monitors since these provide information on the global structure of the peptide and are therefore likely to be sensitive to changes in the structure such as the chirality inversions in the bridging *bis*-thioether moiety. Given that the most likely candidate based on the global percentage of satisfied monitors (11*S*, 22α*R*, 22β*S*, 27*R*) failed to satisfy either of the long-range distance monitors, this was removed from consideration. Of the remaining structures, three were seen to satisfy one long-range monitor but only the 11*R*, 22α*S*, 22β*S*, 27*S* stereoisomer was able to satisfy both and for this reason we concluded that this was the most likely structure based on the available data.

It should be noted that in these cases the monitored distances showed very high standard deviations, as is to be expected from long-range flexible interactions such as these, and that distance satisfaction is based on the observation of minimum distances that agree with the NMR data under the assumption that simulation on the time-scale of the experiments (if this was possible) would yield sufficient numbers of such short distances to produce the observed experimental signal (Supplementary Tables 1, 4-5, Fig. 29-31 and 34-36).

5. Antiproliferative/Cytotoxicity Assays

The antiproliferative activity of **7** was tested against a panel of cancer cell lines, including Lung normal (ATCC CCL-171), Skin cancer (ATCC CRL-11147), Breast cancer (ATCC HTB-22) and Colon cancer (ATCC HTB-38) (Supplementary Table 6). Cell lines (2000 cells/well) were added, respectively, to 96-well plates (Nunc, Thermo Fisher Scientific, US) in Dulbecco's Modified Eagle Medium (DMEM) containing 10% foetal bovine serum (FBS) and gentamicin (10 µg/mL) using Biomek 2000 laboratory automation workstation controlled by Biomek software v 5.0. Cells were incubated for 24 h at 37 °C and maintained in a humidified atmosphere of 5% CO_2 and low passage. At the end of 24 h, the test compound (0.1, 1, 2.5, 5, 10, 12.5, 25, and 50 µM) was added to the wells and incubated for 72 h. Cell viability was determined by a colorimetric MTS assay using a tetrazolium dye, 3-(4,5-dimethylthiazol-2-yl)-5-(3-carboxymethoxyphenyl)-2-(4-sulfophenyl)-2H-tetrazolium, inner salt, and an electron coupling reagent, phenazine methosulfate (PMS). Cell Titer 96®Aqueous One Solution Reagent (Promega, Madison, WI, USA) (10 µL) was added to each well and then incubated for 1 h at 37 °C. The absorbance was recorded on a plate reader at 490 nm. The number of living cells was determined by measuring their ability to reduce the tetrazolium salt to the coloured formazan product at 490 nm. Staurosporine (Sigma) was used as standard reference. IC_{50} values were calculated by nonlinear regression curve fitting using GraphPad Prism 9.0 software.

6. Antibacterial assays

Nine bacterial strains (*Enterococcus faecalis* (ATCC 29212); Methicillin resistant *Staphylococcus aureus* subsp. *aureus* (MRSA) (ATCC 33591); *Staphylococcus aureus* subsp. *aureus* (ATCC 25923); *Streptococcus agalactiae* group B (ATCC 12386); *Escherichia coli* (ATCC 25922), *Pseudomonas aeruginosa* (ATCC 27853) and three clinical isolates (*Enterococcus faecium* K60-39, *Enterococcus faecium* K59-68 and *Staphylococcus haemolyticus*) were used in this study (Supplementary Table 6). Clinical isolates were provided as courtesy of Professor Kristin Hegstad (University Hospital of North Norway). Minimal inhibitory concentration (MIC) assays were adapted according to the recommendations of the Clinical and Laboratory Standard Institute (CLSIM07). MICs were tested using 96-well microdilution plates in Mueller–Hinton broth (Becton Dickinson, UK) at 37 °C; dilutions of the compound were made in triplicate in 96-well culture dishes using Biomek 2000 laboratory automation workstation controlled by Biomek software v 5.0. Strains were taken from an exponentially growing culture and diluted to 5 × 10^5 CFU/mL. The bacteria were cultured in the presence of tested compounds for about 20 h and bacterial growth was monitored visually following the CLSI guidelines. MIC_{50} values were calculated by nonlinear regression curve fitting using GraphPad Prism 9.0 software.

7. Heterologous expression of the *kin* BGC

A plasmid pCAP03-*kin*LR was constructed to capture the kintamdin gene cluster from *Streptomyces sp*. RK44 genomic DNA. The 1034 bp 5' and 1210 bp 3' homology arms were inserted into the *Spe*I site and the *Kpn*I site of pCAP03-*acc(3)IV* using In-Fusion® HD Cloning Kit (Takara Bio, Inc), respectively. The resulting capture vector was linearized with *Xho*I and *Nde*I to release the targeting hooks and co-transformed with *Asc*I-digested genomic DNA into yeast spheroplasts. Preparation of yeast spheroplast cells and transformation, isolation of candidate colonies, colony PCR screening and DNA extraction from PCR positive yeast clones were conducted according to previously reported protocol[2]. Desired transformants were identified by PCR with three pairs of primers (Metrans_L_Hdfor/Xbrev, PM16MP_L_Hdfor/Xbrev and Prep_L_Hdfor/Xbrev) listed in Supplementary Table 11.

The plasmids from three positive clones were recovered and transformed into *E. coli* DH10B for amplification and further restriction analysis of the purified construct (pCAP03-*kin*). Apramycin resistance gene amplified from pIJ773 was then inserted into the *Nhe*I site of verified pCAP03-*kin* to yield pCAP03-*kin*2. *E. coli* ET12567/pUZ8002 was transformed with pCAP03-*kin*2, and transformants were then used to transfer pCAP03-*kin*2 into *Streptomyces coelicolor* M1152 by intergeneric conjugation. Nalidixic acid (50 μg/mL) and apramycin-resistant (50 μg/mL) exconjugants containing integrated pCAP03-*kin*2 were verified by PCR with the three pairs of primers mentioned above.

8. PCR targeting of the *orf*s in the *kin* BGC

To perform the in-frame deletion of the *orfs* contained in pCAP03-*kin*2, the λ-RED mediated PCR-targeting technique was applied following reference[3]. PCR targeting primer couples (Supplementary Table 11) were designed to amplify the *aadA* resistance cassette from *Eco*RI/*Hin*dIII digested pIJ778. The resulting cassettes were then transferred into *E. coli* BW25113/pIJ790/pCAP03-*kin*2 to delete *orf(-2)*, *orf(-1)*, *kinC* to *kinF*, *kinH* to *kinJ*, *kinO* and *orf1*, respectively. After transformation into *E. coli* DH10B for amplification and verification, the obtained pCAP03-*kin*2 derived plasmids were digested with *Nsi*I and re-ligated to remove the *aadA* cassette. These constructs were then introduced into *S. coelicolor* M1152 by conjugation and selecting for apramycin resistance.

9. Purification of the recombinant KinI and KinO in *E. coli*

The genes *kinO* and *kinI* were amplified from the plasmid pCAP03-*kin* using KOD hot start DNA polymerase (Novagen) and the primers listed in Supplementary Table 11. The resulting PCR products were inserted into pET-28a(+) using In-Fusion® HD Cloning Kit (Takara Bio, Inc) to yield pET-28a-*kinO* and pET-28a-*kinI*, respectively. For protein purification, the two constructs above were individually transformed into *E. coli* BL21(DE3). Single colonies from each transformation were grown overnight in 5 mL LB media containing kanamycin (50 μg/mL). The overnight culture was transferred to 200 mL fresh LB medium supplemented with kanamycin (50 μg/mL) and cultivated at 37 °C until the cell density reached an OD_{600} of 0.6. IPTG was added to a final concentration of 0.1 mM to induce protein expression. After induction, cells were grown for 16-20 h at 16 °C and then harvested by centrifugation at 4 °C. The cell pellets were resuspended in buffer A (50 mM Tris-HCl, 0.3 M NaCl, 10 mM imidazole, pH 8.0), followed by lysis with an ultrasonic processor. To remove cell debris, the lysate was centrifuged (5000 g, 4 °C for 15 min). The supernatant was filtered through a 0.45 μm filter prior to purification with HIS-Select® Nickel Affinity Gel (Millipore). The resin was washed with 10 mL of buffer A, followed by two times of washing with buffer B (50 mM Tris-HCl, 0.3 M NaCl, 20 mM imidazole, pH 8.0). The recombinant protein was eluted with 5 mL of buffer C containing 200 mM imidazole and concentrated to 1.0 mL using an Amicon Ultra-15 Centrifugal Filter Unit (Millipore). The protein solution was then desalted by PD-10 Columns (GE Healthcare) according to manufacturer's instruction. The purified protein was store at -80 °C in storage buffer (100 mM Tris-HCl, pH 8.0, 150 mM NaCl, 10% (w/v) glycerol, 1 mM DTT).

10. Biochemical assay for methyltransferase KinO

N-His_6–tagged methyltransferase KinO (~10 μM) was incubated with nonmethylated kintaridin at 28 °C for 1.5 h in a 50 μL volume of reaction buffer (50 mM Tris HCl, 2 mM TCEP, 3 mM S-adenosyl methionine, 10 mM $MgCl_2$, pH 8.0). The reaction mixture was quenched with two volumes of methanol and then subject to Q-TOF analysis.

11. Cultivation of *Streptomyces kurssanovii*

Streptomyces kurssanovii was grown on GYM plates for 5–7 days. A small piece of the GYM plate was used to inoculate 5 mL of GYM medium in a 50 mL Eppendorf conical tube. The seed culture was incubated at 28°C for 48 h at 160 rpm. The fermentation step was initiated by inoculating the seed culture (1 mL) into conical flasks (250 mL) containing different *Streptomyces* media (50 mL) (ISP2-7 and GYM) with. After 7-day cultivation (28°C, 160 rpm), Diaion® HP-20 resin (5 g/L) was added into the cultures. After filtration of the cultures through glass wool, the HP-20 resins were extracted with methanol. The resultant crude extracts were subjected to concentration under reduced pressure prior Q-TOF MS analyses using Bruker DataAnalysis software.

Supplementary tables

Supplementary Table 1. Fragmentation ions of **7** observed in HR-MS analysis.

Ion	z	Observed m/z	Calculated m/z	Mass error (ppm)
b3	1+	308.1970	308.1969	-0.3
b4	1+	391.2340	391.2340	-0.1
b5	1+	490.3024	490.3024	0.1
b6	1+	573.3393	573.3395	0.3
b7	1+	642.3608	642.3610	0.2
b8	1+	711.3798	711.3824	3.7
b11	1+	1056.5176	1056.5183	0.6
b12	1+	1155.5871	1155.5867	-0.4
b13	1+	1226.6246	1226.6238	-0.6
b13	2+	613.8159	613.8155	-0.6
b14	1+	1339.7089	1339.7079	-0.8
b14	2+	670.3570	670.3576	0.9
b15	1+	1438.7780	1438.7763	-1.2
b15	2+	719.8912	719.8918	0.7
b16	1+	1509.8152	1509.8134	-1.2
b16	2+	755.4104	755.4103	0.0
b17	1+	1580.8521	1580.8505	-1.0
b17	2+	790.9286	790.9289	0.4
b18	1+	1651.8889	1651.8876	-0.8
b18	2+	826.4475	826.4475	-0.1
b19	1+	1750.9592	1750.9560	-1.8
b19	2+	875.9814	875.9817	0.3
b20	1+	1833.9915	1833.9932	0.9
b21	1+	1905.0320	1905.0303	-0.9
b21	2+	953.0186	953.0188	0.2
y16	1+	1452.7304	1452.7304	0.0
y15	1+	1353.6619	1353.6620	0.1
y14	1+	1282.6257	1282.6249	-0.6
y13	1+	1169.5415	1169.5408	-0.6
y12	1+	1070.4727	1070.4724	-0.3
y11	1+	999.4353	999.4353	-0.1
y10	1+	928.3977	928.3982	0.5
y9	1+	857.3611	857.3611	0.0
y8	1+	758.2926	758.2926	-0.1
y7	1+	675.2556	675.2555	-0.1
y6	1+	604.2184	604.2184	0.1

Supplementary Table 2. Chemical shifts of NMR analysis of **7**.

**Residue**	**Position**	**dC[a]**	**dH mult (*J* in Hz)[a]**
NMe_2-Ile-1	1	166.9, C	
	2	72.8, CH	3.88*
	3	34.8, CH	2.17*
	4	27.2, CH_2	1.71, br m; 1.37, br m
	5	13.5, CH_3	1.05, d, 6.8
	6	11.8, CH_3	1.07*
	N$(CH_3)_2$	42.5, 2 x CH_3	2.92, s
Dhb-2	1	166.2, C	
	2	131.7, C	
	3	128.5, CH	6.44, q, 7.2
	4	13.3, CH_3	1.88, d, 7.2
	NH		10.16 br s
Dhb-3	1	166.8, C	
	2	131.1, C	
	3	132.8, CH	6.67, q, 7.2
	4	13.4, CH_3	1.88, d, 7.2
	NH		9.80, br s
Dhb-4	1	175.1, C	
	2	129.5, C	
	3	131.0, CH	6.63, q, 7.2
	4	13.4, CH_3	1.81, d, 7.2
	NH		9.16, br s
Val-5	1	174.6, C	
	2	65.5, CH	3.73, d, 9.8
	3	30.4, CH	2.38, br m
	4	21.1, CH_3	1.12, d, 6.4
	5	19.7, CH_3	0.91, d, 6.4
	NH	7.97	br s
Dhb-6	1	168.2, C	
	2	131.0, C	
	3	131.5, CH	6.55, q, 7.2
	4	13.4, CH3	1.82, d, 7.2
	NH		9.22, br s
Aaa-7	1	172.1, C	
	2	99.6, CH	5.44, d, 7.3
	3	135.8, CH	7.20, d, 7.3
	NH		10.21, b, s
Dha-8	1	169.2, C	
	2	139.2, C	
	3	109.6, CH_2	5.52, br s; 5.33, br s
	NH		9.68, br s
Glu-9	1	176.7, C	
	2	57.8, CH	4.12*
	2	26.5, CH_2	2.15*

	3	31.3, CH_2	2.57, t, 6.9
	5	176.1, C	
	OH		8.38, br s
	NH		9.06, br d, 4.4
Ile-10	1	174.7, C	
	2	63.3, CH	3.87*
	3	36.4, CH	2.21*
	4	27.8, CH_2	1.75*; 1.34*
	5	13.6, CH_3	1.03*
	6	10.8, CH_3	0.98*
	NH		7.81, br d, 6.4
Ala(S_1)-11	1	172.9, C	
	2	56.6, CH	4.45, br m
	3	33.8, CH_2	3.34*; 2.88*
	NH		7.92, br s
Val-12	1	175.5, C	
	1	66.0, CH	3.45, dd, 10.6, 4.6
	2	30.4, CH	2.16*
	3	21.1, CH_3	1.03, d, 6.6
	4	19.6, CH_3	0.99, d, 6.4
	NH		7.91, br s
Ala-13	1	173.3, C	
	1	50.7, CH	3.85*
	2	15.3, CH_3	1.41, d, 7.3
	NH		8.85, br s
Leu-14	1	174.6, C	
	2	57.1, CH	4.10*
	3	41.1, CH_2	1.97*; 1.51*
	4	25.5, CH	1.96*
	5	24.0, CH_3	0.90, d, 6.5
	6	21.4, CH_3	0.90, d, 6.5
	NH		8.23, br s
Val-15	1	173.9, C	
	2	65.4, CH	3.69*
	3	30.5, CH	2.24*
	4	21.2, CH_3	1.10, d, 7.8
	5	19.7, CH_3	1.07, d, 7.8
	NH		8.01, br d, 5.8
Ala-16	1	175.5, C	
	2	53.8, CH	3.93*
	3	16.8, CH_3	1.48, d, 7.32
	NH	n.a	
Ala-17	1	174.2, C	
	2	50.8, CH	4.06*
	3	14.7, CH_3	1.45, d, 6.9
	NH		8.44, br d, 7.24
Ala-18	1	175.6, C	

	2	50.8, CH	3.97*
	3	14.8, CH_3	1.46*
	NH		7.85 br s
Val-19	1	C	
	2	65.5, CH	3.75*
	3	30.5, CH	2.24*
	4	21.2, CH_3	1.09, d, 7.8
	5	19.7, CH_3	1.06, d, 7.8
	NH	n.a	8.55, br s
Dhb-20	1	169.0, C	
	2	132.5, C	
	3	129.4, CH	6.46, q, 7.2
	4	12.8, CH_3	1.79, d, 7.5
	NH		9.60, br s
Ala-21	1	178.5.0, C	
	2	53.9, CH	4.21, q, 7.5
	3	16.7, CH_3	1.56, d, 7.3
	NH		8.47, br s
Abu(S_2)-22	1	173.6, C	
	2	57.9, CH	3.64*
	3	43.6, CH	3.62*
	4	19.8, CH_3	1.26,d, 6.2
	NH		8.42, br d, 8.7
Trp-23	1	178.5, C	
	2	59.6, CH	4.38, br m
	3	28.0, CH_2	3.28, br m; 3.25, br m
	4	110.2, CH	
	5	128.5, CH	
	6	119.0, CH	7.53, dd, 7.80, 7.5
	7	119.97, CH	6.99, dd, 7.9, 7.5
	8	122.5, CH	7.06, dd, 7.8, 7.5
	9	112.4, CH	7.29, d, 8.6
	10	138.2, C	
	11	124.8, CH	7.60, s
	NH		9.0, br s
	NH-indole		10.3, br d, 9.7
Asp-24	1	174.7, C	
	2	54.2, CH	4.50, dd, 10.7, 3.5
	3	36.2, CH_2	3.06, dd, 16.6, 10.7; 2.80, br d, 16.6
	4	174.5, C	
	OH		n.a
	NH		n.a
Ser-25	1	175.4, C	
	2	57.0, CH	4.60, br m
	3	63.5, CH_2	3.97, bm; 3.79, br m
	NH		8.03, br d, 9.1
	OH		8.37, br s

Gly-26	1	172.1, C	
	2	46.0, CH_2	3.95*
	NH		n.a
Aminoethane-1,2-dithiol(S1, S2)-27	1	60.0, CH	3.89, br m
	2	33.4, CH_2	3.10, br m; 2.89, br m
	NH		7.96, br s

[a] overlapping with other isochronous ^{13}C, ^{1}H signals

* No multiplicity is given for ^{1}H-NMR signals for which coupling constants (*J*s) could not be measured directly in ^{1}H or determined from HSQC

n.a No data is given for ^{1}H NMR for which no chemical shift was visible from either CD_3OD or CD_3OH

Supplementary Table 3. The retention time comparison of derivatized standard amino acids and amino acid residues from kintamdin **7** after acidic hydrolysis.

RT/min	D-AAs	L-AAs	2.5kDa	Configuration
Trp	13.8	12.8	12.80	L
Asp	10.5	9.63	9.92	L
Glu	11.8	10.9	10.70	L
Ile	14.69	12.57	12.50	L
Leu	15.4	14.99	15.00	L
Val	13.86	11.75	11.74	L
Ser	10.15	9.78	9.92	L
Ala	12.00	10.8	10.81	L: D=2:3

Supplementary Table 4. Computational calculation of distances of key correlations in NOE among eight diastereoisomers in **7**.

Simulations were performed using the GFN2-xTB method at 298 K and included solvent (methanol) effects using a generalized Born continuum treatment. The systems studied were pre-equilibrated for 200 ps prior to beginning data collection. All calculations were performed with the xTB software version 6.3.0 (https://github.com/grimme-lab/xtb).

All distances (and nOe strengths/ranges) given in Å.

11*R*,22α*R*,22β*S*,27*R*: GFN2-xTB molecular dynamics distance monitors.

nOe	Range	Min.	Max.	Mean	S.D.
Short					
Ile-1 (NMe) – Ile-1 (Hα)	1.8-3.0	1.72	2.54	2.15	0.12
Ile-1 (Hγ) – Dhb-2 (NH)	1.8-3.0	1.55	3.39	2.36	0.29
Dhb-2 (NH) – Dhb-2 (Hγ)	1.8-3.0	1.72	3.88	2.60	0.57
Dhb-2 (Hβ) – Dhb-3 (NH)	1.8-3.0	1.62	3.27	2.26	0.28
Dhb-3 (NH) – Dhb-3 (Hγ)	1.8-3.0	1.53	2.82	2.11	0.18
Dhb-3 (Hβ) – Dhb-4 (NH)	1.8-3.0	3.44	5.36	4.57	0.35
Dhb-4 (NH) – Dhb-4 (Hγ)	1.8-3.0	1.54	3.05	2.20	0.21
Val-5 (Hα) – Dhb-6 (NH)	1.8-3.0	3.56	4.91	4.50	0.16
Dhb-6 (NH) – Dhb-6 (Me)	1.8-3.0	1.88	3.56	2.62	0.30
Dha-8 (NH) – Dha-8 (Hβ)	1.8-3.0	1.81	3.36	2.37	0.18
Dha-8 (Hβ) – Glu-9 (Hβ)	1.8-3.0	4.05	6.18	5.41	0.28
Dha-8 (Hβ) – Glu-9 (NH)	1.8-3.0	3.28	4.82	4.31	0.23
Glu-9 (NH) – Glu-9 (Hα)	1.8-3.0	2.62	3.12	2.91	0.07
Glu-9 (Hα) – Ile-10 (NH)	1.8-3.0	2.37	3.25	2.87	0.12
Glu-9 (Hβ) – Ile-10 (NH)	1.8-3.0	3.07	4.09	3.61	0.15
Ile-10 (NH) – Ile-10 (Hα)	1.8-3.0	2.26	2.97	2.63	0.11
Ile-10 (NH) – Ile-10 (Hβ)	1.8-3.0	1.85	3.4	2.43	0.19
Ile-10 (NH) – Ile-10 (Hγ)	1.8-3.0	3.09	3.92	3.56	0.11
Ala(S_1)-11 (NH) – Ala(S_1)-11 (Hα)	1.8-3.0	2.50	3.05	2.86	0.08
Ala(S_1)-11 (NH) – Ala(S_1)-11 (Hβ)	1.8-3.0	2.21	2.88	2.59	0.10
Ala(S_1)-11 (Hα) – Val-12 (NH)	1.8-3.0	3.10	3.84	3.55	0.11
Val-12 (NH) – Val-12 (Hα)	1.8-3.0	2.64	3.08	2.91	0.06
Val-12 (NH) – Val-12 (Hβ)	1.8-3.0	1.70	3.22	2.39	0.20
Leu-14 (NH) – Leu-14 (Hα)	1.8-3.0	2.16	3.05	2.74	0.13
Leu-14 (NH) – Leu-14 (Hβ)	1.8-3.0	2.19	2.94	2.55	0.11
Leu-14 (Hα) – Val-15 (NH)	1.8-3.0	3.21	3.87	3.54	0.10
Ala-17 (NH) – Ala-17 (Hα)	1.8-3.0	1.95	2.66	2.34	0.11
Val-19 (NH) – Val-19 (Hα)	1.8-3.0	2.74	3.11	2.95	0.05
Val-19 (Hβ) – Dhb-20 (NH)	1.8-3.0	1.79	3.51	2.44	0.28
Dhb-20 (NH) – Dhb-20 (Hγ)	1.8-3.0	4.02	5.50	5.03	0.26
Ala-21 (Hα) – Abu(S_2)-22 (NH)	1.8-3.0	3.19	3.81	3.50	0.10
Abu(S_2)-22 (NH) – Abu(S_2)-22 (Hα)	1.8-3.0	1.92	2.59	2.23	0.10
Abu(S_2)-22 (NH) – Abu(S_2)-22 (Hβ)	1.8-3.0	3.11	4.07	3.72	0.13
Abu(S_2)-22 (Hα) – Abu(S_2)-22 (Hβ)	1.8-3.0	2.86	3.20	3.03	0.05
Abu(S_2)-22 (Hα) – Trp-23 (NH)	1.8-3.0	2.46	3.74	3.31	0.20
Trp-23 (NH) – Trp-23 (Hα)	1.8-3.0	2.57	3.09	2.92	0.07
AED-27 (NH) – AED-27 (Hα)	1.8-3.0	1.87	2.52	2.21	0.10
Medium					
Ala(S_1)-11 (NH) – AED-27 (Hα)	1.8-4.0	2.50	3.05	2.86	0.08
Ala(S_1)-11 (NH) – AED-27 (Hβ)	1.8-4.0	3.90	5.05	4.46	0.17
Ala(S_1)-11 (Hβ) – AED-27 (NH)	1.8-4.0	3.21	4.46	3.75	0.18
Ala-21 (Hα) – Asp-24 (Hβ)	1.8-4.0	2.32	5.42	3.49	0.54
Long					
Aaa-7 (CH) – Abu(S_2)-22 (Me)	1.8-5.0	13.40	16.74	15.23	0.48
Leu-14 (Hβ) – AED-27 (Hα)	1.8-5.0	6.60	9.79	8.18	0.58

11*R*,22α*S*,22β*S*,27*R*: GFN2-xTB molecular dynamics distance monitors.

nOe	Range	Min.	Max.	Mean	S.D.
Short					
Ile-1 (NMe) – Ile-1 (Hα)	1.8-3.0	1.66	2.77	2.08	0.15
Ile-1 (Hγ) – Dhb-2 (NH)	1.8-3.0	1.74	3.73	2.52	0.32
Dhb-2 (NH) – Dhb-2 (Hγ)	1.8-3.0	1.62	3.80	2.75	0.52
Dhb-2 (Hβ) – Dhb-3 (NH)	1.8-3.0	1.72	4.64	4.05	0.52
Dhb-3 (NH) – Dhb-3 (Hγ)	1.8-3.0	1.63	3.76	2.22	0.30
Dhb-3 (Hβ) – Dhb-4 (NH)	1.8-3.0	1.63	4.78	3.68	0.77
Dhb-4 (NH) – Dhb-4 (Hγ)	1.8-3.0	1.75	3.70	2.35	0.37
Val-5 (Hα) – Dhb-6 (NH)	1.8-3.0	1.67	4.25	2.36	0.33
Dhb-6 (NH) – Dhb-6 (Me)	1.8-3.0	1.64	2.71	2.20	0.15
Dha-8 (NH) – Dha-8 (Hβ)	1.8-3.0	1.79	2.91	2.34	0.16
Dha-8 (Hβ) – Glu-9 (Hβ)	1.8-3.0	4.48	6.10	5.58	0.21
Dha-8 (Hβ) – Glu-9 (NH)	1.8-3.0	2.69	4.65	4.08	0.27
Glu-9 (NH) – Glu-9 (Hα)	1.8-3.0	2.30	3.06	2.76	0.11
Glu-9 (Hα) – Ile-10 (NH)	1.8-3.0	2.71	3.33	3.01	0.09
Glu-9 (Hβ) – Ile-10 (NH)	1.8-3.0	2.47	3.68	3.05	0.20
Ile-10 (NH) – Ile-10 (Hα)	1.8-3.0	2.12	2.87	2.54	0.11
Ile-10 (NH) – Ile-10 (Hβ)	1.8-3.0	2.00	3.59	2.71	0.22
Ile-10 (NH) – Ile-10 (Hγ)	1.8-3.0	1.78	2.74	2.29	0.14
Ala(S_1)-11 (NH) – Ala(S_1)-11 (Hα)	1.8-3.0	2.52	3.05	2.87	0.07
Ala(S_1)-11 (NH) – Ala(S_1)-11 (Hβ)	1.8-3.0	2.17	2.90	2.55	0.11
Ala(S_1)-11 (NH) – AED-27 (Hβ)	1.8-3.0	3.74	5.07	4.46	0.19
Val-12 (NH) – Val-12 (Hα)	1.8-3.0	2.75	3.12	2.93	0.05
Val-12 (NH) – Val-12 (Hβ)	1.8-3.0	1.84	3.39	2.54	0.22
Leu-14 (NH) – Leu-14 (Hα)	1.8-3.0	2.18	3.07	2.80	0.15
Leu-14 (NH) – Leu-14 (Hβ)	1.8-3.0	1.71	2.90	2.34	0.28
Leu-14 (Hα) – Val-15 (NH)	1.8-3.0	3.02	3.81	3.53	0.10
Ala-17 (NH) – Ala-17 (Hα)	1.8-3.0	2.61	3.08	2.93	0.06
Val-19 (NH) – Val-19 (Hα)	1.8-3.0	2.72	3.09	2.94	0.05
Val-19 (Hβ) – Dhb-20 (NH)	1.8-3.0	2.02	4.51	3.48	0.68
Dhb-20 (NH) – Dhb-20 (Hγ)	1.8-3.0	4.23	5.54	5.00	0.28
Ala-21 (Hα) – Abu(S_2)-22 (NH)	1.8-3.0	3.25	3.88	3.55	0.11
Abu(S_2)-22 (NH) – Abu(S_2)-22 (Hα)	1.8-3.0	2.52	3.10	2.90	0.07
Abu(S_2)-22 (NH) – Abu(S_2)-22 (Hβ)	1.8-3.0	3.30	4.15	3.74	0.14
Abu(S_2)-22 (Hα) – Abu(S_2)-22 (Hβ)	1.8-3.0	2.04	2.73	2.42	0.11
Abu(S_2)-22 (Hα) – Trp-23 (NH)	1.8-3.0	2.89	3.78	3.45	0.12
Trp-23 (NH) – Trp-23 (Hα)	1.8-3.0	2.58	3.08	2.91	0.06
AED-27 (NH) – AED-27 (Hα)	1.8-3.0	1.92	2.67	2.24	0.10
Medium					
Ala(S_1)-11 (NH) – AED-27 (Hα)	1.8-4.0	2.52	3.05	2.87	0.07
Ala(S_1)-11 (Hα) – Val-12 (NH)	1.8-4.0	2.33	3.83	3.30	0.25
Ala(S_1)-11 (Hβ) – AED-27 (NH)	1.8-4.0	2.82	4.35	3.61	0.21
Ala-21 (Hα) – Asp-24 (Hβ)	1.8-4.0	2.20	6.67	4.18	1.29

Long					
Aaa-7 (CH) – Abu(S_2)-22 (Me)	1.8-5.0	14.34	16.54	15.48	0.31
Leu-14 (Hβ) – AED-27 (Hα)	1.8-5.0	4.90	10.58	8.01	1.06

11*R*,22α*R*,22β*S*,27*S*: GFN2-xTB molecular dynamics distance monitors.

nOe	Range	Min.	Max.	Mean	S.D.
Short					
Ile-1 (NMe) – Ile-1 (Hα)	1.8-3.0	1.67	2.49	2.04	0.12
Ile-1 (Hγ) – Dhb-2 (NH)	1.8-3.0	1.66	3.54	2.33	0.29
Dhb-2 (NH) – Dhb-2 (Hγ)	1.8-3.0	1.66	2.94	2.17	0.19
Dhb-2 (Hβ) – Dhb-3 (NH)	1.8-3.0	3.53	5.41	4.33	0.37
Dhb-3 (NH) – Dhb-3 (Hγ)	1.8-3.0	1.70	3.66	2.58	0.43
Dhb-3 (Hβ) – Dhb-4 (NH)	1.8-3.0	3.31	4.69	4.28	0.15
Dhb-4 (NH) – Dhb-4 (Hγ)	1.8-3.0	1.70	3.75	2.95	0.58
Val-5 (Hα) – Dhb-6 (NH)	1.8-3.0	3.43	4.8	4.12	0.18
Dhb-6 (NH) – Dhb-6 (Hγ)	1.8-3.0	1.85	3.67	2.51	0.35
Dha-8 (NH) – Dha-8 (Hβ)	1.8-3.0	1.87	3.02	2.44	0.17
Dha-8 (Hβ) – Glu-9 (NH)	1.8-3.0	2.64	4.65	3.86	0.31
Dha-8 (Hβ) – Glu-9 (Hβ)	1.8-3.0	3.85	5.99	5.03	0.35
Glu-9 (NH) – Glu-9 (Hα)	1.8-3.0	2.56	3.11	2.93	0.06
Glu-9 (Hα) – Ile-10 (NH)	1.8-3.0	2.55	3.70	3.24	0.19
Glu-9 (Hβ) – Ile-10 (NH)	1.8-3.0	3.05	4.20	3.74	0.19
Ile-10 (NH) – Ile-10 (Hα)	1.8-3.0	1.97	3.11	2.70	0.31
Ile-10 (NH) – Ile-10 (Hβ)	1.8-3.0	1.75	3.90	3.09	0.44
Ile-10 (NH) – Ile-10 (Hγ)	1.8-3.0	3.20	4.25	3.81	0.18
Ala(S_1)-11 (NH) – Ala(S_1)-11 (Hα)	1.8-3.0	1.94	3.12	2.55	0.31
Ala(S_1)-11 (NH) – Ala(S_1)-11 (Hβ)	1.8-3.0	1.77	2.97	2.54	0.32
Ala(S_1)-11 (NH) – AED-27 (Hβ)	1.8-3.0	3.52	5.86	4.81	0.31
Val-12 (NH) – Val-12 (Hα)	1.8-3.0	2.62	3.08	2.91	0.06
Val-12 (NH) – Val-12 (Hβ)	1.8-3.0	1.86	4.03	2.90	0.63
Leu-14 (NH) – Leu-14 (Hα)	1.8-3.0	1.92	3.05	2.29	0.16
Leu-14 (NH) – Leu-14 (Hβ)	1.8-3.0	1.73	2.92	2.56	0.20
Leu-14 (Hα) – Val-15 (NH)	1.8-3.0	1.86	3.31	2.48	0.22
Ala-17 (NH) – Ala-17 (Hα)	1.8-3.0	2.66	3.09	2.91	0.06
Val-19 (NH) – Val-19 (Hα)	1.8-3.0	2.75	3.09	2.94	0.05
Val-19 (Hβ) – Dhb-20 (NH)	1.8-3.0	1.85	4.76	3.00	0.76
Dhb-20 (NH) – Dhb-20 (Hγ)	1.8-3.0	4.35	5.51	4.99	0.25
Ala-21 (Hα) – Abu(S_2)-22 (NH)	1.8-3.0	3.17	3.77	3.47	0.09
Abu(S_2)-22 (NH) – Abu(S_2)-22 (Hα)	1.8-3.0	1.95	2.64	2.32	0.10
Abu(S_2)-22 (NH) – Abu(S_2)-22 (Hβ)	1.8-3.0	2.23	3.13	2.61	0.14
Abu(S_2)-22 (Hα) – Abu(S_2)-22 (Hβ)	1.8-3.0	2.23	3.13	2.61	0.14
Abu(S_2)-22 (Hα) – Trp-23 (NH)	1.8-3.0	1.82	3.60	2.59	0.19
Trp-23 (NH) – Trp-23 (Hα)	1.8-3.0	2.61	3.12	2.93	0.06
AED-27 (NH) – AED-27 (Hα)	1.8-3.0	2.77	3.09	2.94	0.05
Medium					
Ala(S_1)-11 (NH) – AED-27 (Hα)	1.8-4.0	1.94	3.12	2.55	0.31
Ala(S_1)-11 (Hα) – Val-12 (NH)	1.8-4.0	3.10	3.86	3.53	0.11
Ala(S_1)-11 (Hβ) – AED-27 (NH)	1.8-4.0	2.64	4.20	3.61	0.23
Ala-21 (Hα) – Asp-24 (Hβ)	1.8-4.0	3.51	5.84	4.62	0.34
Long					
Aaa-7 (CH) – Abu(S_2)-22 (Me)	1.8-5.0	2.88	18.14	13.98	3.82
Leu-14 (Hβ) – AED-27 (Hα)	1.8-5.0	6.05	9.56	7.69	0.57

11*R*,22α*S*,22β*S*,27*S*: GFN2-xTB molecular dynamics distance monitors.

nOe	Range	Min.	Max.	Mean	S.D.
Short					
Ile-1 (NMe) – Ile-1 (Hα)	1.8-3.0	1.63	2.41	2.02	0.10
Ile-1 (Hγ) – Dhb-2 (NH)	1.8-3.0	1.73	3.55	2.34	0.29
Dhb-2 (NH) – Dhb-2 (Hγ)	1.8-3.0	1.71	3.47	2.37	0.28
Dhb-2 (Hβ) – Dhb-3 (NH)	1.8-3.0	3.12	4.72	4.12	0.23
Dhb-3 (NH) – Dhb-3 (Hγ)	1.8-3.0	1.95	3.82	2.96	0.39
Dhb-3 (Hβ) – Dhb-4 (NH)	1.8-3.0	1.61	4.52	3.34	0.76
Dhb-4 (NH) – Dhb-4 (Hγ)	1.8-3.0	1.76	3.75	2.47	0.44
Val-5 (Hα) – Dhb-6 (NH)	1.8-3.0	3.36	4.78	4.32	0.22
Dhb-6 (NH) – Dhb-6 (Me)	1.8-3.0	1.70	2.87	2.20	0.17
Dha-8 (NH) – Dha-8 (Hβ)	1.8-3.0	1.84	2.97	2.35	0.16
Dha-8 (Hβ) – Glu-9 (Hβ)	1.8-3.0	3.80	5.95	5.24	0.31
Dha-8 (Hβ) – Glu-9 (NH)	1.8-3.0	2.74	4.59	3.90	0.33
Glu-9 (NH) – Glu-9 (Hα)	1.8-3.0	2.42	3.06	2.80	0.10
Glu-9 (Hα) – Ile-10 (NH)	1.8-3.0	3.12	3.88	3.52	0.11
Glu-9 (Hβ) – Ile-10 (NH)	1.8-3.0	2.19	3.46	2.89	0.19
Ile-10 (NH) – Ile-10 (Hα)	1.8-3.0	2.52	3.04	2.85	0.08
Ile-10 (NH) – Ile-10 (Hβ)	1.8-3.0	1.75	3.13	2.40	0.20
Ile-10 (NH) – Ile-10 (Hγ)	1.8-3.0	3.13	3.94	3.53	0.11
Ala(S_1)-11 (NH) – Ala(S_1)-11 (Hα)	1.8-3.0	2.35	3.05	2.70	0.10
Ala(S_1)-11 (NH) – Ala(S_1)-11 (Hβ)	1.8-3.0	1.60	2.42	1.98	0.10
Ala(S_1)-11 (NH) – AED-27 (Hβ)	1.8-3.0	2.30	5.20	4.18	0.47
Val-12 (NH) – Val-12 (Hα)	1.8-3.0	2.46	3.09	2.86	0.08
Val-12 (NH) – Val-12 (Hβ)	1.8-3.0	3.27	4.00	3.63	0.10
Leu-14 (NH) – Leu-14 (Hα)	1.8-3.0	1.82	2.46	2.18	0.10
Leu-14 (NH) – Leu-14 (Hβ)	1.8-3.0	2.12	2.99	2.65	0.12
Leu-14 (Hα) – Val-15 (NH)	1.8-3.0	2.32	3.89	3.54	0.11
Ala-17 (NH) – Ala-17 (Hα)	1.8-3.0	2.23	3.11	2.90	0.08
Val-19 (NH) – Val-19 (Hα)	1.8-3.0	2.48	3.12	2.91	0.08
Val-19 (Hβ) – Dhb-20 (NH)	1.8-3.0	1.63	3.90	2.63	0.39
Dhb-20 (NH) – Dhb-20 (Hγ)	1.8-3.0	4.03	5.05	4.54	0.15
Ala-21 (Hα) – Abu(S_2)-22 (NH)	1.8-3.0	1.77	2.97	2.23	0.17
Abu(S_2)-22 (NH) – Abu(S_2)-22 (Hα)	1.8-3.0	1.77	2.97	2.23	0.17
Abu(S_2)-22 (NH) – Abu(S_2)-22 (Hβ)	1.8-3.0	3.05	3.93	3.57	0.12
Abu(S_2)-22 (Hα) – Abu(S_2)-22 (Hβ)	1.8-3.0	2.15	3.07	2.56	0.14
Abu(S_2)-22 (Hα) – Trp-23 (NH)	1.8-3.0	1.80	3.72	2.81	0.34
Trp-23 (NH) – Trp-23 (Hα)	1.8-3.0	2.24	3.39	2.79	0.20
AED-27 (NH) – AED-27 (Hα)	1.8-3.0	2.75	3.11	2.96	0.05
Medium					
Ala(S_1)-11 (NH) – AED-27 (Hα)	1.8-4.0	2.35	3.05	2.70	0.10
Ala(S_1)-11 (Hα) – Val-12 (NH)	1.8-4.0	3.19	3.90	3.57	0.10
Ala(S_1)-11 (Hβ) – AED-27 (NH)	1.8-4.0	3.03	4.41	3.68	0.22

Ala-21 (Hα) – Asp-24 (Hβ)	1.8-4.0	5.20	8.23	6.68	0.45
Long					
Aaa-7 (CH) – Abu(S_2)-22 (Me)	1.8-5.0	3.97	12.85	9.27	1.64
Leu-14 (Hβ) – AED-27 (Hα)	1.8-5.0	4.00	7.95	6.30	0.51

11*S*,22α*R*,22β*S*,27*R*: GFN2-xTB molecular dynamics distance monitors.

nOe	Range	Min.	Max.	Mean	S.D.
Short					
Ile-1 (NMe) – Ile-1 (Hα)	1.8-3.0	1.71	2.58	2.07	0.13
Ile-1 (Hγ) – Dhb-2 (NH)	1.8-3.0	1.74	3.67	2.46	0.25
Dhb-2 (NH) – Dhb-2 (Hγ)	1.8-3.0	1.87	3.86	2.79	0.45
Dhb-2 (Hβ) – Dhb-3 (NH)	1.8-3.0	1.57	3.40	2.17	0.25
Dhb-3 (NH) – Dhb-3 (Hγ)	1.8-3.0	1.63	2.81	2.23	0.18
Dhb-3 (Hβ) – Dhb-4 (NH)	1.8-3.0	1.75	4.34	2.62	0.42
Dhb-4 (NH) – Dhb-4 (Hγ)	1.8-3.0	1.59	3.56	2.24	0.24
Val-5 (Hα) – Dhb-6 (NH)	1.8-3.0	2.30	4.84	4.19	0.42
Dhb-6 (NH) – Dhb-6 (Me)	1.8-3.0	1.74	2.87	2.30	0.17
Dha-8 (NH) – Dha-8 (Hβ)	1.8-3.0	1.86	3.09	2.40	0.18
Dha-8 (Hβ) – Glu-9 (Hβ)	1.8-3.0	4.75	6.11	5.61	0.20
Dha-8 (Hβ) – Glu-9 (NH)	1.8-3.0	3.20	4.66	4.13	0.26
Glu-9 (NH) – Glu-9 (Hα)	1.8-3.0	2.24	3.09	2.79	0.15
Glu-9 (Hα) – Ile-10 (NH)	1.8-3.0	2.10	3.90	3.19	0.43
Glu-9 (Hβ) – Ile-10 (NH)	1.8-3.0	2.27	3.66	3.02	0.37
Ile-10 (NH) – Ile-10 (Hα)	1.8-3.0	2.47	3.08	2.88	0.10
Ile-10 (NH) – Ile-10 (Hβ)	1.8-3.0	1.84	3.50	2.69	0.24
Ile-10 (NH) – Ile-10 (Hγ)	1.8-3.0	1.76	3.67	2.60	0.42
Ala(S_1)-11 (NH) – Ala(S_1)-11 (Hα)	1.8-3.0	1.90	2.58	2.24	0.11
Ala(S_1)-11 (NH) – Ala(S_1)-11 (Hβ)	1.8-3.0	2.22	3.01	2.70	0.13
Ala(S_1)-11 (NH) – AED-27 (Hβ)	1.8-3.0	1.68	3.95	2.58	0.35
Val-12 (NH) – Val-12 (Hα)	1.8-3.0	2.60	3.12	2.92	0.06
Val-12 (NH) – Val-12 (Hβ)	1.8-3.0	1.82	4.01	3.04	0.63
Leu-14 (NH) – Leu-14 (Hα)	1.8-3.0	1.88	2.60	2.24	0.10
Leu-14 (NH) – Leu-14 (Hβ)	1.8-3.0	1.94	2.99	2.61	0.14
Leu-14 (Hα) – Val-15 (NH)	1.8-3.0	1.95	3.38	2.66	0.22
Ala-17 (NH) – Ala-17 (Hα)	1.8-3.0	2.46	3.07	2.91	0.07
Val-19 (NH) – Val-19 (Hα)	1.8-3.0	2.67	3.10	2.93	0.06
Val-19 (Hβ) – Dhb-20 (NH)	1.8-3.0	1.87	4.47	2.64	0.37
Dhb-20 (NH) – Dhb-20 (Hγ)	1.8-3.0	4.28	5.53	4.92	0.27
Ala-21 (Hα) – Abu(S_2)-22 (NH)	1.8-3.0	2.74	3.83	3.39	0.14
Abu(S_2)-22 (NH) – Abu(S_2)-22 (Hα)	1.8-3.0	1.95	2.55	2.24	0.09
Abu(S_2)-22 (NH) – Abu(S_2)-22 (Hβ)	1.8-3.0	3.30	4.17	3.82	0.13
Abu(S_2)-22 (Hα) – Abu(S_2)-22 (Hβ)	1.8-3.0	2.69	3.17	2.98	0.07
Abu(S_2)-22 (Hα) – Trp-23 (NH)	1.8-3.0	2.33	3.50	2.97	0.17
Trp-23 (NH) – Trp-23 (Hα)	1.8-3.0	2.51	3.05	2.84	0.08
AED-27 (NH) – AED-27 (Hα)	1.8-3.0	2.76	3.08	2.94	0.05
Medium					
Ala(S_1)-11 (NH) – AED-27 (Hα)	1.8-4.0	1.90	2.58	2.24	0.11
Ala(S_1)-11 (Hα) – Val-12 (NH)	1.8-4.0	1.87	3.1	2.51	0.19
Ala(S_1)-11 (Hβ) – AED-27 (NH)	1.8-4.0	3.46	4.48	4.05	0.15
Ala-21 (Hα) – Asp-24 (Hβ)	1.8-4.0	3.26	5.61	4.44	0.34

Long					
Aaa-7 (CH) – Abu(S_2)-22 (Me)	1.8-5.0	10.69	16.18	14.19	0.88
Leu-14 (Hβ) – AED-27 (Hα)	1.8-5.0	5.74	9.18	7.32	0.49

11*S*,22α*S*,22β*S*,27*R*: GFN2-xTB molecular dynamics distance monitors.

nOe	Range	Min.	Max.	Mean	S.D.
Short					
Ile-1 (NMe) – Ile-1 (Hα)	1.8-3.0	1.63	2.50	2.02	0.14
Ile-1 (Hγ) – Dhb-2 (NH)	1.8-3.0	1.78	4.43	3.44	0.46
Dhb-2 (NH) – Dhb-2 (Hγ)	1.8-3.0	1.60	3.78	2.63	0.58
Dhb-2 (Hβ) – Dhb-3 (NH)	1.8-3.0	1.90	4.73	3.92	0.64
Dhb-3 (NH) – Dhb-3 (Hγ)	1.8-3.0	1.74	3.71	3.06	0.38
Dhb-3 (Hβ) – Dhb-4 (NH)	1.8-3.0	3.19	4.59	4.21	0.18
Dhb-4 (NH) – Dhb-4 (Hγ)	1.8-3.0	1.71	2.98	2.27	0.19
Val-5 (Hα) – Dhb-6 (NH)	1.8-3.0	2.64	4.81	4.28	0.25
Dhb-6 (NH) – Dhb-6 (Me)	1.8-3.0	1.75	3.99	3.25	0.38
Dha-8 (NH) – Dha-8 (Hβ)	1.8-3.0	1.91	3.20	2.42	0.17
Dha-8 (Hβ) – Glu-9 (Hβ)	1.8-3.0	3.51	6.01	5.06	0.47
Dha-8 (Hβ) – Glu-9 (NH)	1.8-3.0	2.13	4.72	3.66	0.46
Glu-9 (NH) – Glu-9 (Hα)	1.8-3.0	2.51	3.06	2.83	0.09
Glu-9 (Hα) – Ile-10 (NH)	1.8-3.0	1.25	2.29	1.72	0.16
Glu-9 (Hβ) – Ile-10 (NH)	1.8-3.0	2.40	3.87	3.16	0.24
Ile-10 (NH) – Ile-10 (Hα)	1.8-3.0	1.95	2.56	2.25	0.10
Ile-10 (NH) – Ile-10 (Hβ)	1.8-3.0	1.79	3.91	3.22	0.13
Ile-10 (NH) – Ile-10 (Hγ)	1.8-3.0	2.84	4.27	3.74	0.17
Ala(S_1)-11 (NH) – Ala(S_1)-11 (Hα)	1.8-3.0	1.90	2.62	2.26	0.10
Ala(S_1)-11 (NH) – Ala(S_1)-11 (Hβ)	1.8-3.0	2.16	2.95	2.66	0.11
Ala(S_1)-11 (NH) – AED-27 (Hβ)	1.8-3.0	4.72	6.71	5.66	0.33
Val-12 (NH) – Val-12 (Hα)	1.8-3.0	2.66	3.11	2.92	0.06
Val-12 (NH) – Val-12 (Hβ)	1.8-3.0	1.85	3.09	2.41	0.20
Leu-14 (NH) – Leu-14 (Hα)	1.8-3.0	1.92	2.57	2.22	0.10
Leu-14 (NH) – Leu-14 (Hβ)	1.8-3.0	2.32	2.95	2.69	0.10
Leu-14 (Hα) – Val-15 (NH)	1.8-3.0	2.11	3.46	2.75	0.20
Ala-17 (NH) – Ala-17 (Hα)	1.8-3.0	2.69	3.10	2.93	0.05
Val-19 (NH) – Val-19 (Hα)	1.8-3.0	2.58	3.08	2.90	0.07
Val-19 (Hβ) – Dhb-20 (NH)	1.8-3.0	2.00	4.62	3.97	0.28
Dhb-20 (NH) – Dhb-20 (Hγ)	1.8-3.0	4.19	5.58	5.05	0.28
Ala-21 (Hα) – Abu(S_2)-22 (NH)	1.8-3.0	2.10	3.83	3.31	0.35
Abu(S_2)-22 (NH) – Abu(S_2)-22 (Hα)	1.8-3.0	2.59	3.11	2.94	0.06
Abu(S_2)-22 (NH) – Abu(S_2)-22 (Hβ)	1.8-3.0	3.37	4.18	3.79	0.13
Abu(S_2)-22 (Hα) – Abu(S_2)-22 (Hβ)	1.8-3.0	2.05	2.80	2.43	0.11
Abu(S_2)-22 (Hα) – Trp-23 (NH)	1.8-3.0	2.79	3.91	3.43	0.16
Trp-23 (NH) – Trp-23 (Hα)	1.8-3.0	2.40	3.07	2.86	0.09
AED-27 (NH) – AED-27 (Hα)	1.8-3.0	1.99	2.58	2.24	0.09
Medium					
Ala(S_1)-11 (NH) – AED-27 (Hα)	1.8-4.0	1.90	2.62	2.26	0.10
Ala(S_1)-11 (Hα) – Val-12 (NH)	1.8-4.0	1.95	3.00	2.43	0.17
Ala(S_1)-11 (Hβ) – AED-27 (NH)	1.8-4.0	2.02	3.87	2.88	0.31
Ala-21 (Hα) – Asp-24 (Hβ)	1.8-4.0	5.13	7.30	6.16	0.30

Long					
Aaa-7 (CH) – Abu(S_2)-22 (Me)	1.8-5.0	12.12	18.80	15.33	1.41
Leu-14 (Hβ) – AED-27 (Hα)	1.8-5.0	3.47	7.59	5.25	0.73

11*S*,22α*R*,22β*S*,27*S*: GFN2-xTB molecular dynamics distance monitors.

nOe	Range	Min.	Max.	Mean	S.D.
Short					
Ile-1 (NMe) – Ile-1 (Hα)	1.8-3.0	1.72	2.72	2.12	0.14
Ile-1 (Hγ) – Dhb-2 (NH)	1.8-3.0	3.00	4.61	3.85	0.26
Dhb-2 (NH) – Dhb-2 (Hγ)	1.8-3.0	1.69	3.03	2.30	0.21
Dhb-2 (Hβ) – Dhb-3 (NH)	1.8-3.0	1.80	4.59	3.08	0.71
Dhb-3 (NH) – Dhb-3 (Hγ)	1.8-3.0	1.68	3.06	2.18	0.18
Dhb-3 (Hβ) – Dhb-4 (NH)	1.8-3.0	1.93	4.67	3.98	0.46
Dhb-4 (NH) – Dhb-4 (Hγ)	1.8-3.0	1.74	3.52	2.28	0.27
Val-5 (Hα) – Dhb-6 (NH)	1.8-3.0	1.60	4.77	2.54	0.51
Dhb-6 (NH) – Dhb-6 (Me)	1.8-3.0	1.68	3.68	2.42	0.37
Dha-8 (NH) – Dha-8 (Hβ)	1.8-3.0	3.20	3.96	3.65	0.11
Dha-8 (Hβ) – Glu-9 (Hβ)	1.8-3.0	5.06	6.10	5.68	0.15
Dha-8 (Hβ) – Glu-9 (NH)	1.8-3.0	3.51	4.82	4.39	0.16
Glu-9 (NH) – Glu-9 (Hα)	1.8-3.0	2.29	3.02	2.75	0.11
Glu-9 (Hα) – Ile-10 (NH)	1.8-3.0	1.74	2.67	2.15	0.14
Glu-9 (Hβ) – Ile-10 (NH)	1.8-3.0	1.99	3.52	2.75	0.23
Ile-10 (NH) – Ile-10 (Hα)	1.8-3.0	1.82	2.69	2.26	0.10
Ile-10 (NH) – Ile-10 (Hβ)	1.8-3.0	3.30	4.07	3.73	0.12
Ile-10 (NH) – Ile-10 (Hγ)	1.8-3.0	2.41	3.71	3.23	0.17
Ala(S_1)-11 (NH) – Ala(S_1)-11 (Hα)	1.8-3.0	1.96	2.54	2.26	0.10
Ala(S_1)-11 (NH) – Ala(S_1)-11 (Hβ)	1.8-3.0	2.25	2.92	2.64	0.11
Ala(S_1)-11 (NH) – AED-27 (Hβ)	1.8-3.0	5.05	6.50	5.82	0.18
Val-12 (NH) – Val-12 (Hα)	1.8-3.0	2.76	3.10	2.94	0.05
Val-12 (NH) – Val-12 (Hβ)	1.8-3.0	1.86	3.54	2.60	0.30
Leu-14 (NH) – Leu-14 (Hα)	1.8-3.0	2.53	3.06	2.86	0.07
Leu-14 (NH) – Leu-14 (Hβ)	1.8-3.0	1.97	2.87	2.52	0.13
Leu-14 (Hα) – Val-15 (NH)	1.8-3.0	1.83	3.05	2.30	0.16
Ala-17 (NH) – Ala-17 (Hα)	1.8-3.0	2.53	3.15	2.91	0.07
Val-19 (NH) – Val-19 (Hα)	1.8-3.0	2.76	3.10	2.94	0.05
Val-19 (Hβ) – Dhb-20 (NH)	1.8-3.0	1.72	4.31	2.53	0.35
Dhb-20 (NH) – Dhb-20 (Hγ)	1.8-3.0	4.20	5.53	4.97	0.24
Ala-21 (Hα) – Abu(S_2)-22 (NH)	1.8-3.0	2.94	3.96	3.57	0.12
Abu(S_2)-22 (NH) – Abu(S_2)-22 (Hα)	1.8-3.0	1.92	2.68	2.30	0.11
Abu(S_2)-22 (NH) – Abu(S_2)-22 (Hβ)	1.8-3.0	3.48	4.26	3.97	0.11
Abu(S_2)-22 (Hα) – Abu(S_2)-22 (Hβ)	1.8-3.0	2.16	2.92	2.56	0.12
Abu(S_2)-22 (Hα) – Trp-23 (NH)	1.8-3.0	2.14	3.26	2.77	0.17
Trp-23 (NH) – Trp-23 (Hα)	1.8-3.0	2.70	3.10	2.94	0.05
AED-27 (NH) – AED-27 (Hα)	1.8-3.0	2.65	3.10	2.92	0.06
Medium					
Ala(S_1)-11 (NH) – AED-27 (Hα)	1.8-4.0	1.96	2.54	2.26	0.10
Ala(S_1)-11 (Hα) – Val-12 (NH)	1.8-4.0	2.00	3.12	2.54	0.18
Ala(S_1)-11 (Hβ) – AED-27 (NH)	1.8-4.0	3.09	4.24	3.73	0.18
Ala-21 (Hα) – Asp-24 (Hβ)	1.8-4.0	4.39	7.62	5.42	0.54

Long					
Aaa-7 (CH) – Abu(S_2)-22 (Me)	1.8-5.0	14.75	18.31	17.04	0.57
Leu-14 (Hβ) – AED-27 (Hα)	1.8-5.0	7.88	10.68	9.07	0.45

11*S*,22α*S*,22β*S*,27*S*: GFN2-xTB molecular dynamics distance monitors.

nOe	Range	Min.	Max.	Mean	S.D.
Short					
Ile-1 (NMe) – Ile-1 (Hα)	1.8-3.0	1.66	2.41	2.04	0.11
Ile-1 (Hγ) – Dhb-2 (NH)	1.8-3.0	1.76	4.06	2.40	0.27
Dhb-2 (NH) – Dhb-2 (Hγ)	1.8-3.0	1.72	3.46	2.40	0.25
Dhb-2 (Hβ) – Dhb-3 (NH)	1.8-3.0	1.61	3.51	2.34	0.27
Dhb-3 (NH) – Dhb-3 (Hγ)	1.8-3.0	1.73	2.91	2.25	0.18
Dhb-3 (Hβ) – Dhb-4 (NH)	1.8-3.0	3.38	4.58	4.08	0.18
Dhb-4 (NH) – Dhb-4 (Hγ)	1.8-3.0	2.84	3.80	3.43	0.12
Val-5 (Hα) – Dhb-6 (NH)	1.8-3.0	1.66	3.53	2.42	0.27
Dhb-6 (NH) – Dhb-6 (Me)	1.8-3.0	1.97	3.56	2.72	0.28
Dha-8 (NH) – Dha-8 (Hβ)	1.8-3.0	1.89	3.05	2.36	0.17
Dha-8 (Hβ) – Glu-9 (Hβ)	1.8-3.0	4.67	6.05	5.55	0.21
Dha-8 (Hβ) – Glu-9 (NH)	1.8-3.0	3.39	4.75	4.23	0.20
Glu-9 (NH) – Glu-9 (Hα)	1.8-3.0	2.34	3.05	2.82	0.10
Glu-9 (Hα) – Ile-10 (NH)	1.8-3.0	1.69	2.70	2.16	0.15
Glu-9 (Hβ) – Ile-10 (NH)	1.8-3.0	1.87	3.45	2.68	0.22
Ile-10 (NH) – Ile-10 (Hα)	1.8-3.0	1.87	2.57	2.26	0.09
Ile-10 (NH) – Ile-10 (Hβ)	1.8-3.0	3.23	4.11	3.71	0.12
Ile-10 (NH) – Ile-10 (Hγ)	1.8-3.0	2.53	3.72	3.24	0.16
Ala(S_1)-11 (NH) – Ala(S_1)-11 (Hα)	1.8-3.0	1.93	2.62	2.29	0.10
Ala(S_1)-11 (NH) – Ala(S_1)-11 (Hβ)	1.8-3.0	2.21	3.00	2.64	0.11
Ala(S_1)-11 (NH) – AED-27 (Hβ)	1.8-3.0	5.02	6.39	5.74	0.21
Val-12 (NH) – Val-12 (Hα)	1.8-3.0	2.73	3.11	2.94	0.05
Val-12 (NH) – Val-12 (Hβ)	1.8-3.0	1.83	3.16	2.47	0.20
Leu-14 (NH) – Leu-14 (Hα)	1.8-3.0	2.54	3.07	2.83	0.08
Leu-14 (NH) – Leu-14 (Hβ)	1.8-3.0	2.08	2.89	2.59	0.12
Leu-14 (Hα) – Val-15 (NH)	1.8-3.0	1.78	2.88	2.26	0.15
Ala-17 (NH) – Ala-17 (Hα)	1.8-3.0	2.55	3.09	2.91	0.07
Val-19 (NH) – Val-19 (Hα)	1.8-3.0	2.71	3.10	2.95	0.05
Val-19 (Hβ) – Dhb-20 (NH)	1.8-3.0	1.68	4.45	2.65	0.36
Dhb-20 (NH) – Dhb-20 (Hγ)	1.8-3.0	4.24	5.47	4.91	0.24
Ala-21 (Hα) – Abu(S_2)-22 (NH)	1.8-3.0	2.38	3.85	3.39	0.22
Abu(S_2)-22 (NH) – Abu(S_2)-22 (Hα)	1.8-3.0	2.26	3.06	2.81	0.11
Abu(S_2)-22 (NH) – Abu(S_2)-22 (Hβ)	1.8-3.0	3.16	3.88	3.56	0.11
Abu(S_2)-22 (Hα) – Abu(S_2)-22 (Hβ)	1.8-3.0	2.23	2.90	2.58	0.12
Abu(S_2)-22 (Hα) – Trp-23 (NH)	1.8-3.0	2.27	3.71	3.17	0.18
Trp-23 (NH) – Trp-23 (Hα)	1.8-3.0	2.69	3.08	2.93	0.05
AED-27 (NH) – AED-27 (Hα)	1.8-3.0	2.70	3.09	2.94	0.05
Medium					
Ala(S_1)-11 (NH) – AED-27 (Hα)	1.8-4.0	1.93	2.62	2.29	0.10
Ala(S_1)-11 (Hα) – Val-12 (NH)	1.8-4.0	1.97	3.06	2.49	0.17
Ala(S_1)-11 (Hβ) – AED-27 (NH)	1.8-4.0	2.26	3.97	3.28	0.24
Ala-21 (Hα) – Asp-24 (Hβ)	1.8-4.0	3.89	7.20	5.62	0.53

Long					
Aaa-7 (CH) – Abu(S_2)-22 (Me)	1.8-5.0	14.04	18.07	16.37	0.72
Leu-14 (Hβ) – AED-27 (Hα)	1.8-5.0	7.56	10.55	8.87	0.53

Supplementary Table 5. Summary of distance monitor satisfaction in the MD simulations of the kintamdin peptide stereoisomers.

	Distance monitors satisfied			
Stereoisomer	Short	Medium	Long	Total
11*R*,22α*R*,22β*S*,27*R*	26 (70%)	4 (100%)	0 (0%)	30 (70%)
11*R*,22α*S*,22β*S*,27*R*	31 (84%)	4 (100%)	1 (50%)	35 (81%)
11*R*,22α*R*,22β*S*,27*S*	29 (78%)	3 (75%)	1 (50%)	33 (77%)
11*R*,22α*S*,22β*S*,27*S*	31 (84%)	3 (75%)	2 (100%)	35 (81%)
11*S*,22α*R*,22β*S*,27*R*	33 (89%)	4 (100%)	0 (0%)	37 (86%)
11*S*,22α*S*,22β*S*,27*R*	32 (86%)	3 (75%)	1 (50%)	35 (81%)
11*S*,22α*R*,22β*S*,27*S*	30 (81%)	3 (75%)	0 (0%)	33 (77%)
11*S*,22α*S*,22β*S*,27*S*	30 (81%)	4 (100%)	0 (0%)	34 (79%)

Supplementary Table 6. Bioassay activity evaluation of kintamdin **7**.

Strains/Cell lines	MIC/IC_{50}(μM)	Gentamicin (Control)	Staurosporine (Control)
Enterococcus faecium K60-39 [b]	19.8	>134	-
Enterococcus faecium K59-68 [b]	>20	>134	-
Staphylococcus haemolyticus [b]	>20	40.2	-
Enterococcus faecalis (ATCC 29212)[a]	14.8	1.0	-
Methicillin resistant *Staphylococcus aureus subsp. aureus* (MRSA) (ATCC 33591) [a]	>20	2.0	-
Staphylococcus aureus subsp. aureus (ATCC 25923) [a]	>20	0.3	-
Streptococcus agalactiae (ATCC 12386) [a]	>20	1.0	-
Escherichia coli (ATCC 25922) [a]	>20	0.3	-
Pseudomonas aeruginosa (ATCC 27853) [a]	>20	1.0	-
Skin cancer cell (ATCC CRL-11147)	2.4	-	9.0
Breast cancer cell (ATCC HTB-22)	0.6	-	0.3
Colon cancer cell (ATCC HTB-38)	12.0	-	5.4
Lung normal cell (ATCC CCL-171)	>20	-	>100

[a] standard strain; [b] clinic isolate (Ref 9)

Supplementary Table 7. Deduced Functions of *orfs* in the kintamdin biosynthetic gene cluster (accession number: MW923391).

Gene	Size[a]	Protein Homolog[b] and Origin	Identities/ Positives, %	Proposed function
orf(-2)[c]	308	YaeI (WP_123975326) from *Streptomyces sp.* Ag109_O5-1	95/97	Metallophosphoesterase
orf(-1)[c]	226	WP_133023397.1 from *Streptomyces sp.* BK329	92/93	Integral membrane regulator
kinA	57	WP_071383936 from *Streptomyces sp.* MUSC 1	50/66	Precursor peptide
kinB	114	WP_108060352 from *Spartobacteria bacterium* LR76	34/50	Hypothetic protein/DUF1153 domain containing protein
kinC	324	WP_015659226 from *Streptomyces davaonensis*	56/69	T3SS effector HopA1 family protein /hypothetic protein
kinD	377	WP_189473175.1 from *Streptomyces kurssanovii*	57/70	Class V lanthionine synthetase subunit LxmK/phosphotransferase
kinE	432	PqqL (WP_060892880.1) from *Streptomyces europaeiscabiei*	59/72	Insulinase family protein/ peptidase M16_C
kinF	458	PqqL (WP_060892881.1) from *Streptomyces europaeiscabiei*	44/63	Insulinase family protein/ peptidase M16_C
kinG	142	WP_153461218.1 from *Streptomyces kaniharaensis*	53/66	Transposase
kinH	311	WP_015659222.1 from *Streptomyces davaonensis*	43/53	Hypothetic protein
kinI	207	WP_060891085.1 from *Streptomyces europaeiscabiei*	66/78	Flavoprotein/phosphopantothenoylcysteine decarboxylase
kinJ	325	WP_060891084.1 from *Streptomyces europaeiscabiei*	69/82	LLM class Flavin dependent oxidoreductase
kinK	280	YadH (WP_060891083) from *Streptomyces europaeiscabiei*	57/75	ABC transporter permease
kinL	272	CcmA (WP_106435783.1) from *Streptomyces davaonensis*	73/81	ABC transporter ATP binding protein
kinM	550	EFL25110.1 from *Streptomyces himastatinicus* ATCC 53653	46/60	ABC transporter, ATP binding protein

kinN	226	CcmA (WP_063729889) from *Streptomyces sp.* RTd22	67/78	ABC transporter ATP binding protein
kinO	253	PrmA (WP_007264900.1) from *Streptomyces sp.* C	69/82	Class I SAM dependent methyltransferase
orf1[c]	511	WP_141594955.1 from *Streptomyces ipomoeae*	95/97	LacI family DNA binding transcriptional regulator
orf2[c]	124	WP_079158480.1 from *Streptomyces*	97/99	Helix-turn-helix domain-containing protein
orf3[c]	186	WP_141594956.1 from *Streptomyces ipomoeae*	95/97	Helix-turn-helix transcriptional regulator

[a] Numbers are in amino acids.
[b] NCBI accession numbers are given in parentheses.
[c] *orfs* beyond the *kin* gene cluster.

Supplementary Table 8 sequence analysis of key ORFs, KinC, D, H and I with the corresponding enzymes in TVA pathway.

Kintamdin **7**			Thioviridamides (TVA) **2**		
ORF	aa	identity	ORF	aa	Proposed functions
KinC (133-312)*	324	32%	TvaD$_{S-87}$ (145-328) * Accession number: WP_030193573.1	335	dehydration
KinD (219-279)*	385	42%	TvaC$_{S-87}$ (182-243) * Accession number: WP_030193576.1	332	phosphorylation
KinH	311	0%	TvaE$_{S-87}$ Accession number: WP_107048384.1	314	cyclization
KinI	207	32%	TvaF$_{S-87}$ Accession number: WP_051794611.1	201	decarboxylation

* indicated that only the AA fragments in the bracket of the proteins of interest show low homologue (aa identity) with the corresponding enzymes in the TVA pathway.

Supplementary Table 9. Plasmids used in this study.

Plasmid	Description	Ref. or Source
pET-28a(+)	*pBR322 ori KanR T7lac f1 N-His6 C-His6*, for recombinant protein expression in *E. coli*	Novagen
pCAP03-*acc(3)IV*	*pUC ori ARSH4/CEN6 Kan/NeoR TRP1 φC31int-attP oriT (RP4) pAHD-URA3*, for TAR cloning	10
pCAP03-*kin*LR	pCAP03-*acc(3)IV*-derived plasmid, for capturing the kintamdin gene cluster	This work
pCAP03-*kin*	pCAP03 containing the kintamdin gene cluster, kan^r	This work
pCAP03-*kin*2	pCAP03-*kin* equipped with apramycin resistance gene	This work
pIJ773	pBluescript KS (+), *aac(3)IV*, *oriT* (RK2), FRT sites	8
pIJ778	pBluescript KS (+), *aadA*, *oriT* (RK2), FRT sites	8
pIJ790	λ-RED (*gam*, *bet*, *exo*), *cat*, *araC*, $rep101^{ts}$	8
pUZ8002	*tra*, *neo*, RP4 derivative	11
pET-28a-*kinO*	For overexpression of *kinO* in *E. coli*	This work
pET-28a-*kinI*	For overexpression of *kinI* in *E. coli*	This work
pETDuet-*kinC*	For overexpression of *kinC* in *E. coli*	Genscript
pCDFDuet-*kinA-kinD*	Co-expression of KinA and KinD in *E. coli*	Genscript
pCDFDuet-sumo-*kinA-kinD*	Co-expression of His_6-Sumo-KinA and KinD in *E. coli*	This work
pETDuet-*kinC-kinI*	Co-expression of His_6-Sumo-KinA and KinCDI in *E. coli*	This work
pRSFDuet-*kin*H	Co-expression of His_6-Sumo-KinA and KinCDHI or KinCDH in *E. coli*	This work

Supplementary Table 10. Strains used in this study.

Strain	Relevant genotype and descriptions	Ref. or Source
Saccharomyces		
S. cerevisiae VL6-48N	*MATα trp1-Δ1 ura3-Δ1 ade2-101 his3-Δ200 lys2 met14 cir°*, host for TAR cloning	10
Escherichia coli		
DH10B	*F⁻ mcrA Δ(mrr-hsdRMS-mcrBC) φ80lacZΔM15 ΔlacX74 recA1 endA1 araD139 Δ (ara-leu)7697 galU galK λ⁻ rpsL(StrR) nupG*, strain used for general cloning and plasmid maintenance	Thermo Scientific
BL21 (DE3)	*fhuA2 [lon] ompT gal (λ DE3) [dcm] ΔhsdS*, widely used T7 expression *E. coli* strain	New England Biolabs
HST08	*F⁻, endA1, supE44, thi-1, recA1, relA1, gyrA96, phoA, Φ80d lacZΔ M15, Δ (lacZYA - argF) U169, Δ (mrr - hsdRMS - mcrBC), ΔmcrA, λ⁻*, for general cloning and plasmid maintenance	Takara, Stellar™ Competent Cells
ET12567	*dam*, *dcm*, *hsdM*, *hsdS*, *hsdR*, *cat*, *tet*, methylation-deficient *E. coli* used for conjugal DNA transfer	12
BW25113	K-12 derivative: Δ*araBAD*, Δ*rhaBAD*	13
***Streptomyces sp.* RK44**		
WT	Wild type, kintamdin producing strain	Ghana isolate
***Streptomyces coelicolor* M1152**		
WT	M145Δ*act* Δ*red* Δ*cpk* Δ*cda rpoB*[C1298T], host for heterologous expression	14
RK8-46	heterologous expression strain containing pCAP03-*kin2*	This work
Δ*kinC*	In frame deletion mutant	This work
Δ*kinD*	In frame deletion mutant	This work
Δ*kinE*	In frame deletion mutant	This work
Δ*kinF*	In frame deletion mutant	This work
Δ*kinH*	In frame deletion mutant	This work

Δ*kinI*	In frame deletion mutant	This work
Δ*kinJ*	In frame deletion mutant	This work
Δ*kinO*	In frame deletion mutant	This work
Δ*orf(-2)*	In frame deletion mutant	This work
Δ*orf(-1)*	In frame deletion mutant	This work
Δ*orf1*	In frame deletion mutant	This work
Streptomyces kurssanovii		
NCIMB12788	kintamdin-like RiPP producer	NCIMB Ltd

Supplementary Table 11. Primers used in this study.

Primer	Sequence	Use
RKtar_Lfor	TCTACAAAGATCGACTAGTCCGACCGCCCGACCTTTTTA	Construction of pCAP03-*kin*LR
RKtar_Lrev	TAGTTGTTGTAGGACTAGTGGTGTACGCCGGGTGGGTTA	
RKtar2_Rfor	GGCCAGCAAAACTAAGGTACCTTCCTCGTCCTCGTGTTAC	
RKtar2_Rrev	ATTTTTCTAAATACAGGTACCCTTTTTGCATGCGGTGTTA	
Metrans_L_Hdfor	AAAAAGCTTCGAAGGCGAGGGCGGCGATGGACAGA	TAR colony screening
Metrans_L_Xbrev	AAATCTAGATGAACACGAGGAAATGAGAGCCCTGCTGGAA	
PM16MP_L_Hdfor	GGTAAGCTTAGACATGCACGCCGCCAGCCAAACATA	
PM16MP_L_Xbrev	CCATCTAGACATTCGTCAGCCCCCACTGTCCCTGGTA	
Prep_L_Hdfor	GCCAAAGCTTAACCCACCCGGCGTACACCAAAAT	
PreP_L_Xbrev	CGTTCTAGACACAGCGACCCCTCAACTTTCCAT	
Metrans_PTfor	aaaatgatcgctgcttgccctgcgggagtgaaccctatgATGCATATTCCGGGGATCCGTCGACC	Construction of Δ*kinO*
Metrans_PTrev	tcctgcaattccagcagggctctcatttcctcgtgttcaATGCATTGTAGGCTGGAGCTGCTTC	
PM16MP_PTfor	cgtccaattggtaccagggacagtgggggctgacgaatgATGCATATTCCGGGGATCCGTCGACC	Construction of Δ*kinF*
PM16MP_PTrev	ccttcgtgcatcccatcgcgcgtgatagtcgtgtcgctaATGCATTGTAGGCTGGAGCTGCTTC	
PPDC_PTfor	atctcgaagagatcttcgccgcgttggatcggcttcatgATGCATATTCCGGGGATCCGTCGACC	Construction of Δ*kinI*
PPDC_PTrev	tcaacgagctgtcgcatcctggaatcaccgctcctcaggATGCATTGTAGGCTGGAGCTGCTTC	
RKMPH_PTfor	gtctgacacgatggtcgtatgcgcgctcgatacggagtaATGCATATTCCGGGGATCCGTCGACC	Construction of Δ*orf(-2)*
RKMPH_PTrev	gcatatctggcattttggtgtacgccgggtgggttactcATGCATTGTAGGCTGGAGCTGCTTC	

RKIMR_PTfor	ggcacacccgtactgtggcccgggcacgttcccgtgacgATGCATATTCCGGGGATCCGTCGACC	Construction of Δ*orf(-1)*
RKIMR_PTrev	actttagcccactggtggccggagacgaaatccggtttaATGCATTGTAGGCTGGAGCTGCTTC	
RKLacI_PTfor	gccgcgtaacacgaggacgaggaaggggaggagtacttgATGCATATTCCGGGGATCCGTCGACC	Construction of Δ*orf1*
RKLacI_PTrev	cagttatcgccaggggcacagcagcattcaccgttggtgATGCATTGTAGGCTGGAGCTGCTTC	
RkpD_PTfor	tgtacacgtatcgacgctgggcttggagaagacgcgatgATGCATATTCCGGGGATCCGTCGACC	Construction of Δ*kinC*
RkpD_PTrev	agcggtctctcctcggtccggcaccggtcacgccaatccATGCATTGTAGGCTGGAGCTGCTTC	
RkpE_PTfor2	ctacggtcgccagacatgattgatagccaggaggtgctgATGCATATTCCGGGGATCCGTCGACC	Construction of Δ*kinD*
RkpE_PTrev2	cagcgtcgatacgtgtacatacgggtcgagtagcaggacATGCATTGTAGGCTGGAGCTGCTTC	
RkpI_PTfor	aatccggacctgaggagcggtgattccaggatgcgacagATGCATATTCCGGGGATCCGTCGACC	Construction of Δ*kinH*
RkpI_PTrev	cctgccggggccgaccaccggacatcacagcttctacagATGCATTGTAGGCTGGAGCTGCTTC	
RkpK_PTfor	tgaccgacagaggacacgatgacgtcaatggaacacagcATGCATATTCCGGGGATCCGTCGACC	Construction of Δ*kinJ*
RkpK_PTrev	tcagccatgaagccgatccaacgcggcgaagatctcttcATGCATTGTAGGCTGGAGCTGCTTC	
PM16C_PTfor	gcgttttgaaacttacccaactcctctagggtgagccacATGCATATTCCGGGGATCCGTCGACC	Construction of Δ*kinE*
PM16C_PTrev	aacctggtccggagcgtgccgggttccactcgttcattcATGCATTGTAGGCTGGAGCTGCTTC	
dMetra_ScrFor	CGCCGCCAGATGGAGAAGGGTTGATTT	Verification of Δ*kinO*
dMetra_ScrRev	GCGAATGCCCGGGTCAGATTTCAG	
dPM16M_ScrFor	GCCATTGCCGCACGGGGACTGAG	Verification of Δ*kinF*
dPM16M_ScrRev	GCAGGAGGGCGCGGCATGTTTTCAGT	
dPPDC_ScrFor	GCTGGTAGCCGGTCCCGAACTCCTCAA	Verification of Δ*kinI*
dPPDC_ScrRev	CAATCCGGCGGGAGTGATCCAGAGTGAG	
dLacI_ScrFor	CCGACACCCAACTTATGCGTAA	Verification of Δ*orf1*
dLacI_ScrRev	CGGCGACTCATCCCCTTCACA	

dRkpD_ScrFor	GCATTCGGCAGCAGTCGCTTTTCCTG	Verification of Δ*kinC*
dRkpD_ScrRev	CATGCACGCTTTCTGGGAAGGCTATT	
dRkpE_ScrFor	GACTCACCAGCCCGCTCGGATTCTCA	Verification of Δ*kinD*
dRkpE_ScrRev	TGTTTGGCTGGCGGCGTGCATGTCTACTG	
dRkpK_ScrFor	AAGTGAAAGCTCGCGGGGTGTATCA	Verification of Δ*kinJ*
dRkpK_ScrRev	ACAGAGGACACGATGACGTCAATGGAA	
dPM16C_ScrFor	TTCATGCAGCACCTCCTGGCTATCAATCA	Verification of Δ*kinE*
dPM16C_ScrRev	AGCGTGCCGGGTTCCACTCGTTCATT	
Prep_L_Hdfor	GCCAAAGCTTAACCCACCCGGCGTACACCAAAAT	Verification of Δ*orf(-1)*
PreP_L_Xbrev	CGTTCTAGACACAGCGACCCCTCAACTTTCCAT	
PM16MP_R_Xbfor	AAATCTAGATAGCGACACGACTATCACGCGCGATGGGAT	Verification of Δ*kinH*
PM16MP_R_Elrev	AAAGAATTCTGATTCCAGGATGCGACAGCTCGTTGAGG	
Metra_for2	GTCGCGGATCCGAATTCATGGACTCGACGCAAACCTATGA	Overexpression of *kinO*
Metra_rev	AGTGCGGCCGCAAGCTTTTCCAGCAGGGCTCTCATTTC	
PPDC_for	GTCGCGGATCCGAATTCATGGCTGAGCCGCCCGTCAGTG	Overexpression of *kinI*
PPDC_rev	AGTGCGGCCGCAAGCTTGAGCTGTCGCATCCTGGAATCAC	
CAPapr-for2	atcgggccctggccaGCTAGCTGGATACACCAAGGAAAGT	Construction of pCAP03-*kin*2
CAPapr-revNheI	tgcaggtcgactctaGCTAGCTTATGAGCTCAGCCAATCG	
Sumo_for	ATCACCACAGCCAGGATCgtATGTCGGACTCAGAAGTCAATCA	Insertion of sumo into pCDFDuet-*kinA*-*kinD*
Sumo_Arev	GCCGCGTTCATCGGATCCGAGCCCTGAAAATACAGGTTTTCACCAATCTGTTCTCTGTGAGCCT	
thr2-ala-f	CGATGCGCCGGCGATCGCGACCACCGTTACCAGC	Construction of *kin*A$_{T2A}$ variant
thr2-ala-r	GCTGGTAACGGTGGTCGCGATCGCCGGCGCATCG	
thr3-ala-f	GCGCCGGCGATCACCGCGACCGTTACCAGCAGC	Construction of *kin*A$_{T3A}$ variant
thr3-ala-r	GCTGCTGGTAACGGTCGCGGTGATCGCCGGCGC	
thr4-ala-f	CCGGCGATCACCACCGCGGTTACCAGCAGCG	

thr4-ala-r	CGCTGCTGGTAACCGCGGTGGTGATCGCCGG	Construction of *kin*A_{T4A} variant
thr6-ala-f	CGATCACCACCACCGTTGCGAGCAGCGAAATTTGC	Construction of *kin*A_{T6A} variant
thr6-ala-r	GCAAATTTCGCTGCTCGCAACGGTGGTGGTGATCG	
ser7-thr-f	CCACCACCGTTACCACCAGCGAAATTTGCGTG	Construction of *kin*A_{S7T} variant
ser7-thr-r	CACGCAAATTTCGCTGGTGGTAACGGTGGTGG	
ser8-ala-f	CCACCGTTACCAGCGCGGAAATTTGCGTGAGC	Construction of *kin*A_{S8A} variant
ser8-ala-r	GCTCACGCAAATTTCCGCGCTGGTAACGGTGG	
cys11-ala-f	CCAGCAGCGAAATTGCGGTGAGCCTGGTTAGC	Construction of *kin*A_{C11A} variant
cys11-ala-r	GCTAACCAGGCTCACCGCAATTTCGCTGCTGG	
PCDFmut-F1	CTTTAATAAGGAGATATACCATGGGCAGCAGCCAT CACCATCATCACCACAGCC	Construction of *kin*A_{TtoA} variant
PCDFmut-R2	CCATTCGCCCACGAAGCCACCGATATCACGCGCCG GGTCACCCAGACGCAGTTCC	
thr2346-ala-f2	GCCGCGGCAGTTGCGAGCAGCGAAATTTGCGTGA GCCTGGTTAGC	
thr2346-ala-r1	GCTCGCAACTGCCGCGGCGATCGCCGGCGCATCG CTCTCCGCCGCC	

Restriction sites are underlined.

Supplementary Table 12. KinA homologs identified by BlastP.

No.	Sequence ID	Size[a]	Organism	Identities/ Positives, %
1	WP_071383936.1	62	*Streptomyces monashensis*	50/66
2	WP_093682639.1	62	MULTISPECIES: *Streptomyces*	50/66
3	WP_210167998.1	77	*Streptomyces* sp. SID4946	50/66
4	WP_015659228.1	61	*Streptomyces davaonensis*	54/62
5	WP_060892876.1	62	*Streptomyces europaeiscabiei*	56/64
6	WP_215178515.1	66	*Streptomyces* sp. ISL-10	55/60
7	WP_180931126.1	65	*Streptomyces* sp. AJS327	57/73
8	WP_167769265.1	55	*Microbispora rosea*	54/68
9	WP_122818067.1	59	MULTISPECIES: *Nocardioides*	57/66
10	WP_189473171.1	66	*Streptomyces kurssanovii*	53/60
11	WP_215178513.1	63	*Streptomyces* sp. ISL-10	62/77
12	WP_183537733.1	55	*Microbispora rosea*	53/66
13	WP_060892875.1	66	*Streptomyces europaeiscabiei*	50/60
14	WP_007264905.1	49	MULTISPECIES: *Streptomyces*	59/77
15	MQS12983.1	63	*Streptomyces kaniharaensis*	51/65
16	WP_183537729.1	63	*Microbispora rosea*	48/62
17	WP_189473172.1	63	*Streptomyces kurssanovii*	57/75
18	WP_015659229.1	66	*Streptomyces davaonensis*	47/59
19	WP_180931127.1	67	*Streptomyces* sp. AJS327	50/64
20	WP_071383935.1	60	*Streptomyces* sp. *monashensis*	74/85
21	WP_093682638.1	60	MULTISPECIES: *Streptomyces*	50/61
22	WP_170837473.1	55	*Actinopolyspora xinjiangensis*	56/59
23	WP_170837471.1	55	*Actinopolyspora xinjiangensis*	51/62
24	WP_054237385.1	66	*Streptomyces* sp. OK228	42/57
25	WP_079089964.1	66	*Streptomyces* sp. OK228	57/70
26	WP_170837472.1	55	*Actinopolyspora xinjiangensis*	53/62
27	WP_210167997.1	78	*Streptomyces* sp. SID4946	50/61
28	WP_153461214.1	65	*Streptomyces kaniharaensis*	43/56
29	WP_155860843.1	59	*Corynebacterium timonense*	44/61
30	WP_211179043.1	79	*Streptomyces* sp. RLA2-12	57/70
31	WP_183537727.1	60	*Microbispora rosea*	44/59
32	WP_215084283.1	72	*Streptomyces* sp. ISL-11	47/67
33	WP_215084281.1	62	*Streptomyces* sp. ISL-11	47/61
34	WP_076436126.1	60	*Microbispora rosea*	44/57
35	WP_078077249.1	60	*Streptomyces niveus*	44/60
36	KPH96831.1	79	*Actinobacteria* bacterium OK006	57/70

[a] Numbers are in amino acids.

Supplementary figures

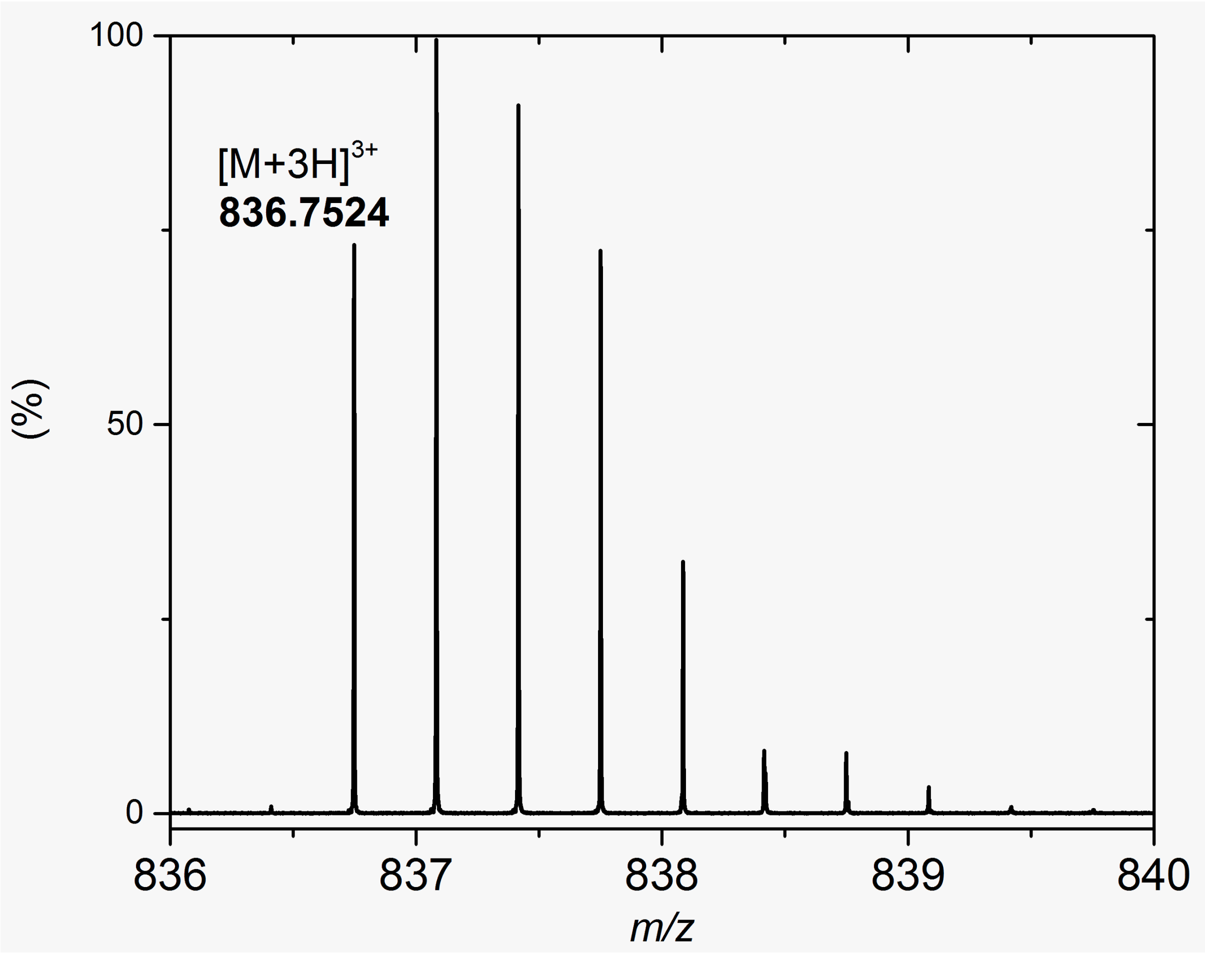

Supplementary Fig. 1. High Resolution ESI FT-ICR Mass Spectrometry analysis of **7.**

MS analysis revealed a dominant $[M+3H]^{3+}$ ion of *m/z* 836.7524, consistent with a neutral molecular mass of 2507.2349 Da.

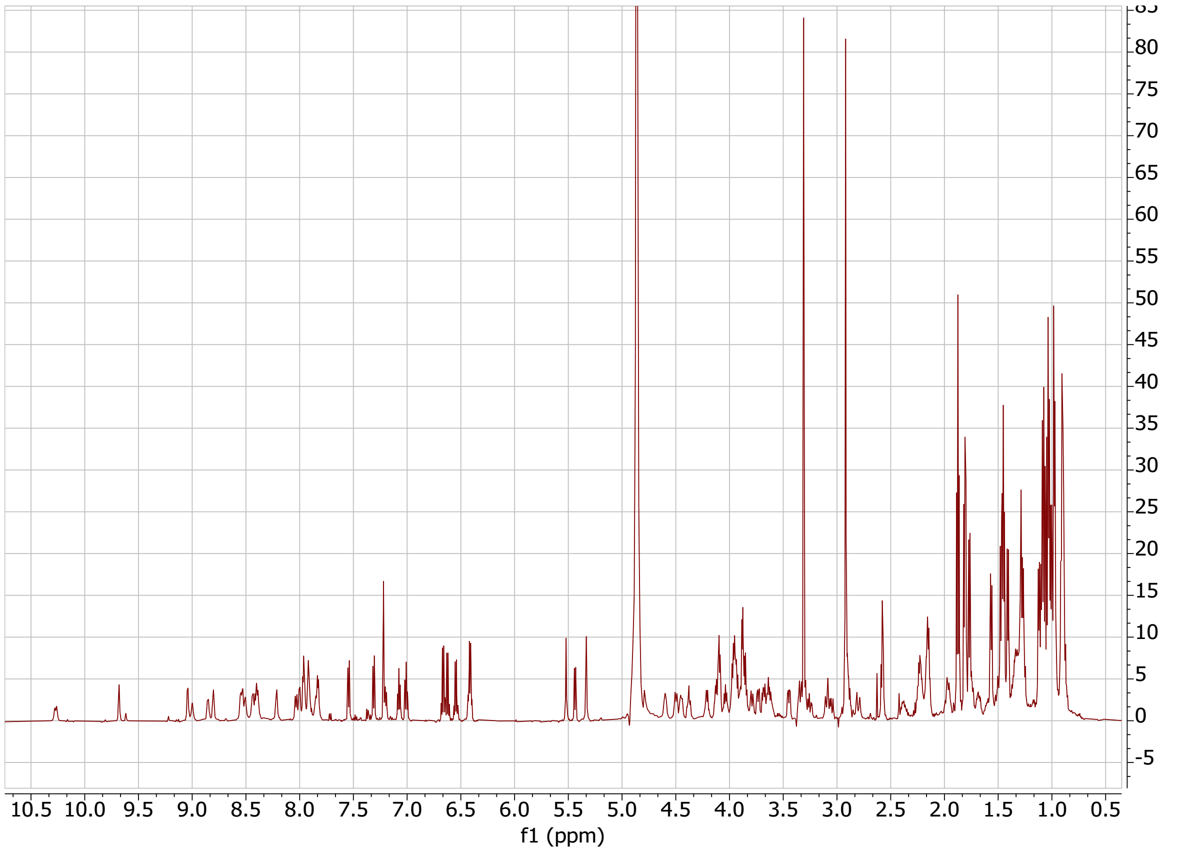

Supplementary Fig. 2. ^{1}H NMR of **7** in CD_3OD at 600 MHz.

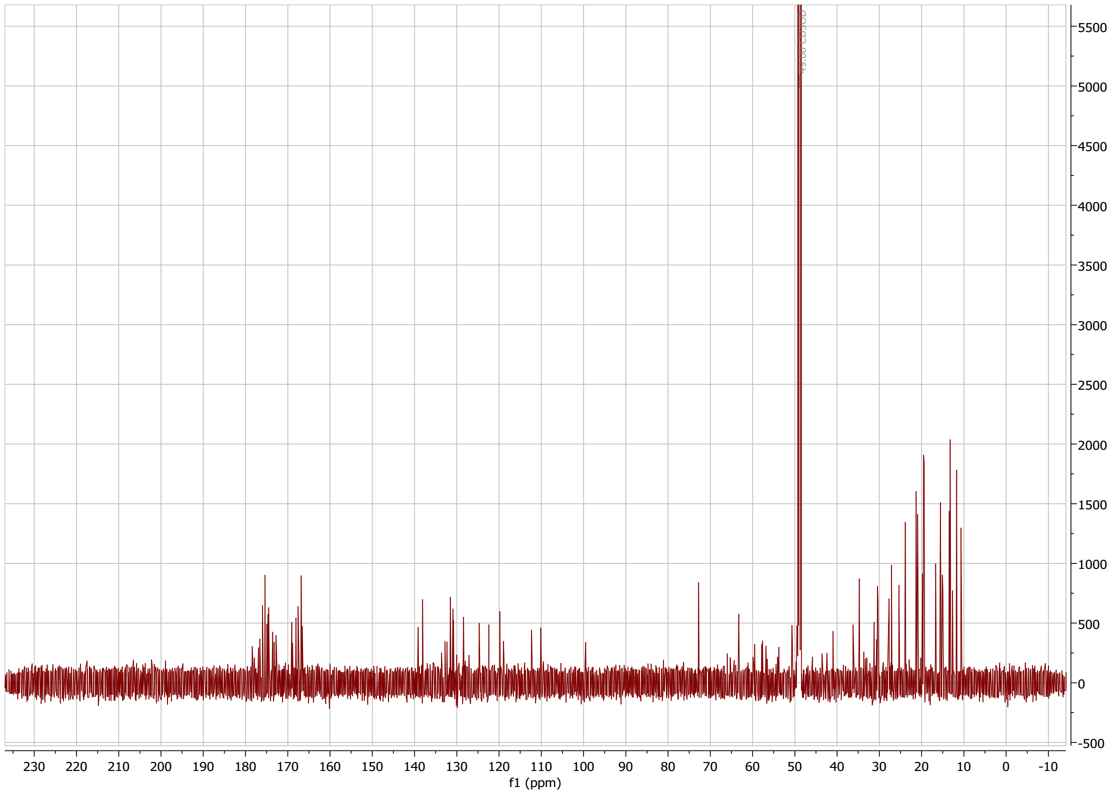

Supplementary Fig. 3. ^{13}C NMR spectrum for **7** in CD_3OD at 150 MHz.

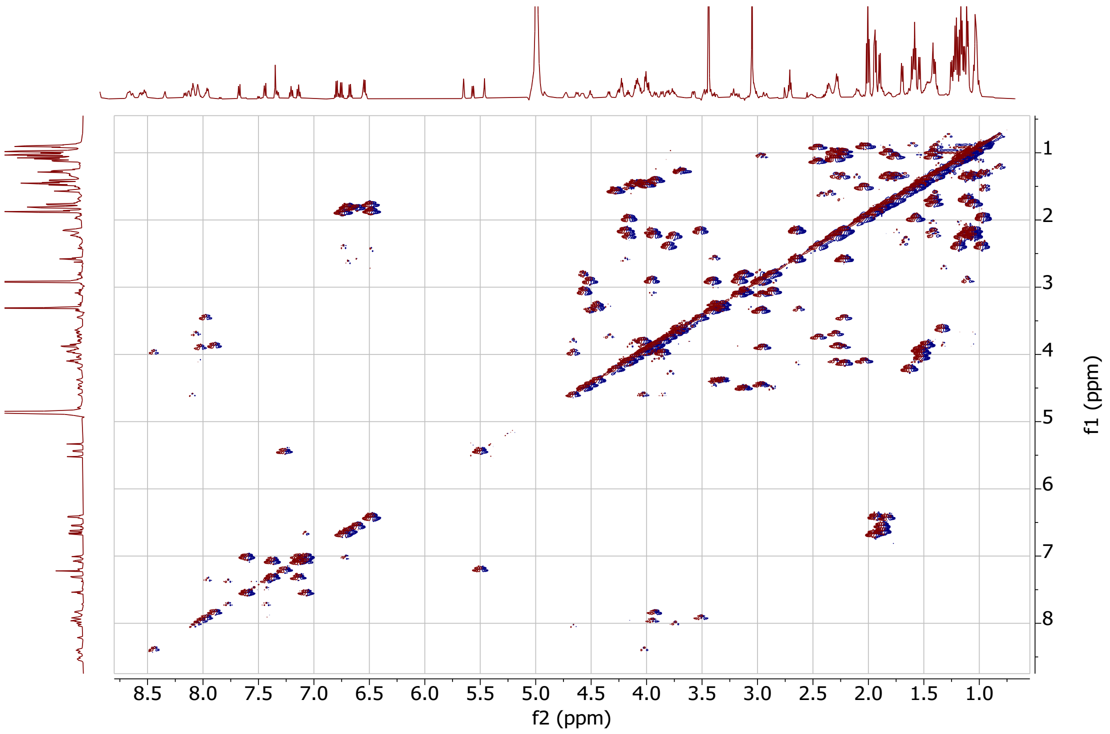

Supplementary Fig. 4. COSY spectrum of **7**.

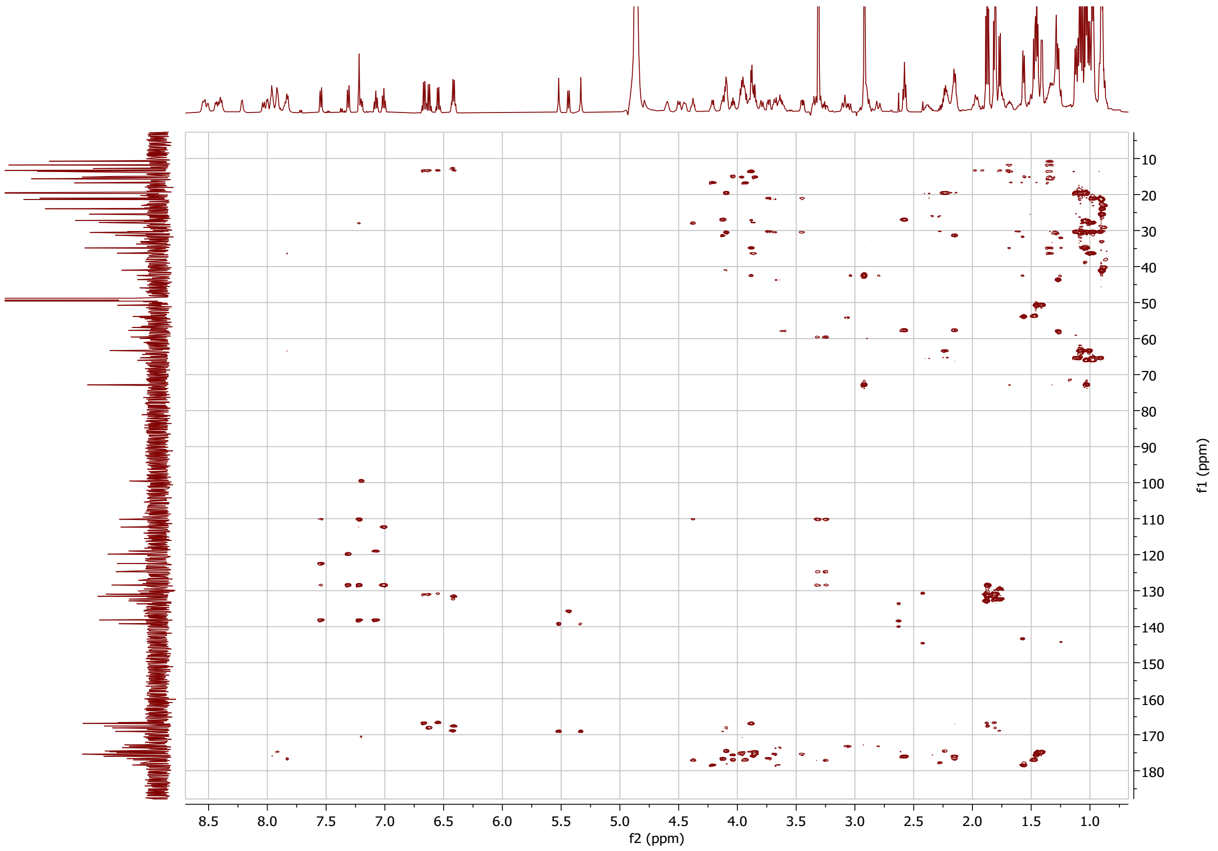

Supplementary Fig. 5. HMBC spectrum of **7**.

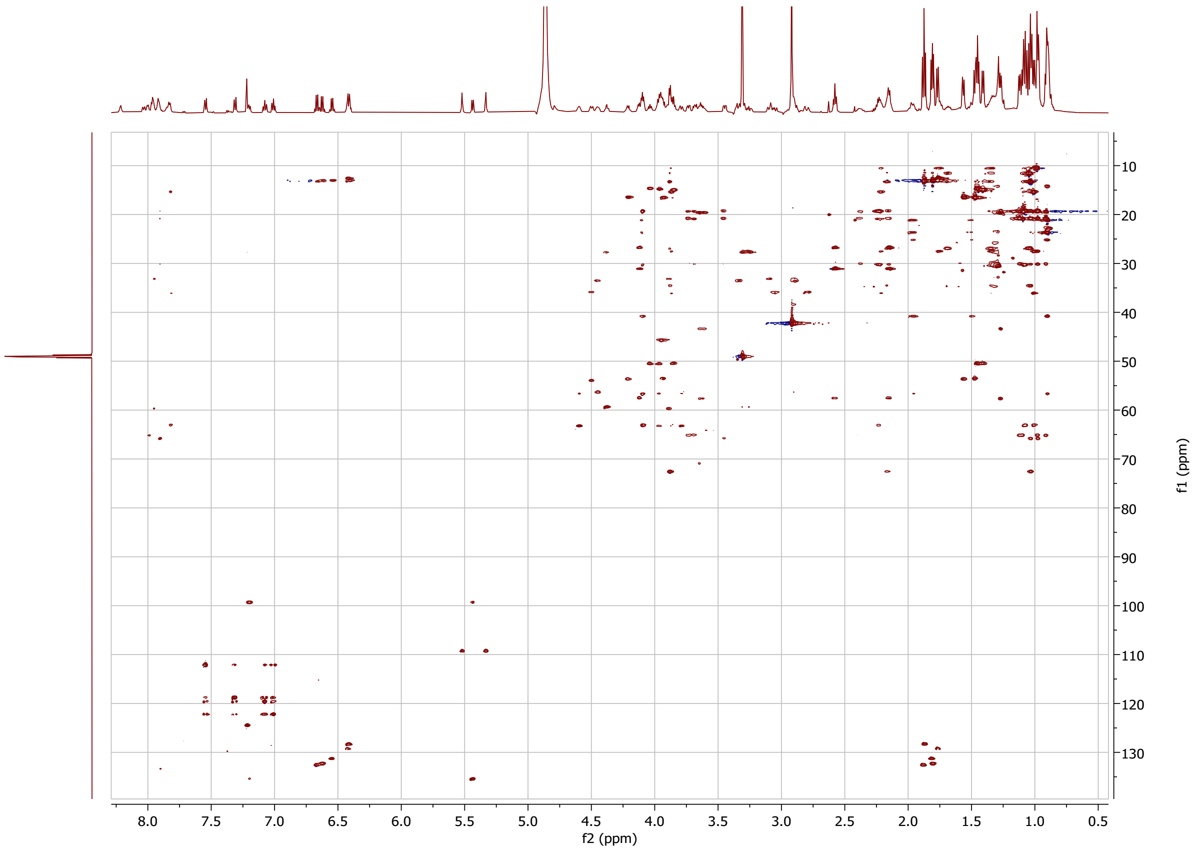

Supplementary Fig. 6. HSQC-TOCSY of **7**.

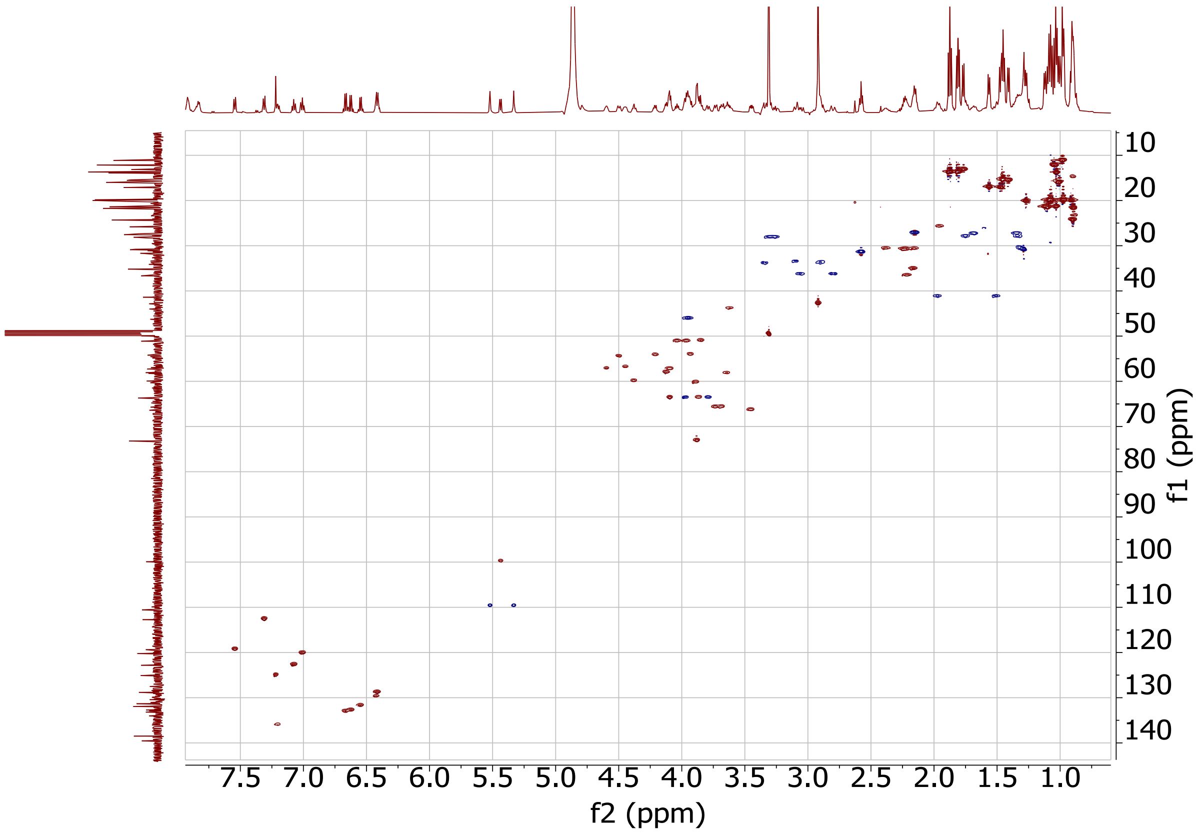

Supplementary Fig. 7. HSQC spectrum of **7**.

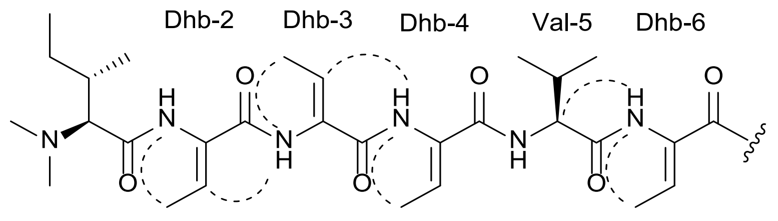

Supplementary Fig. 8. Substructure of **7** containing multiple (*Z*)-Dhb motif.

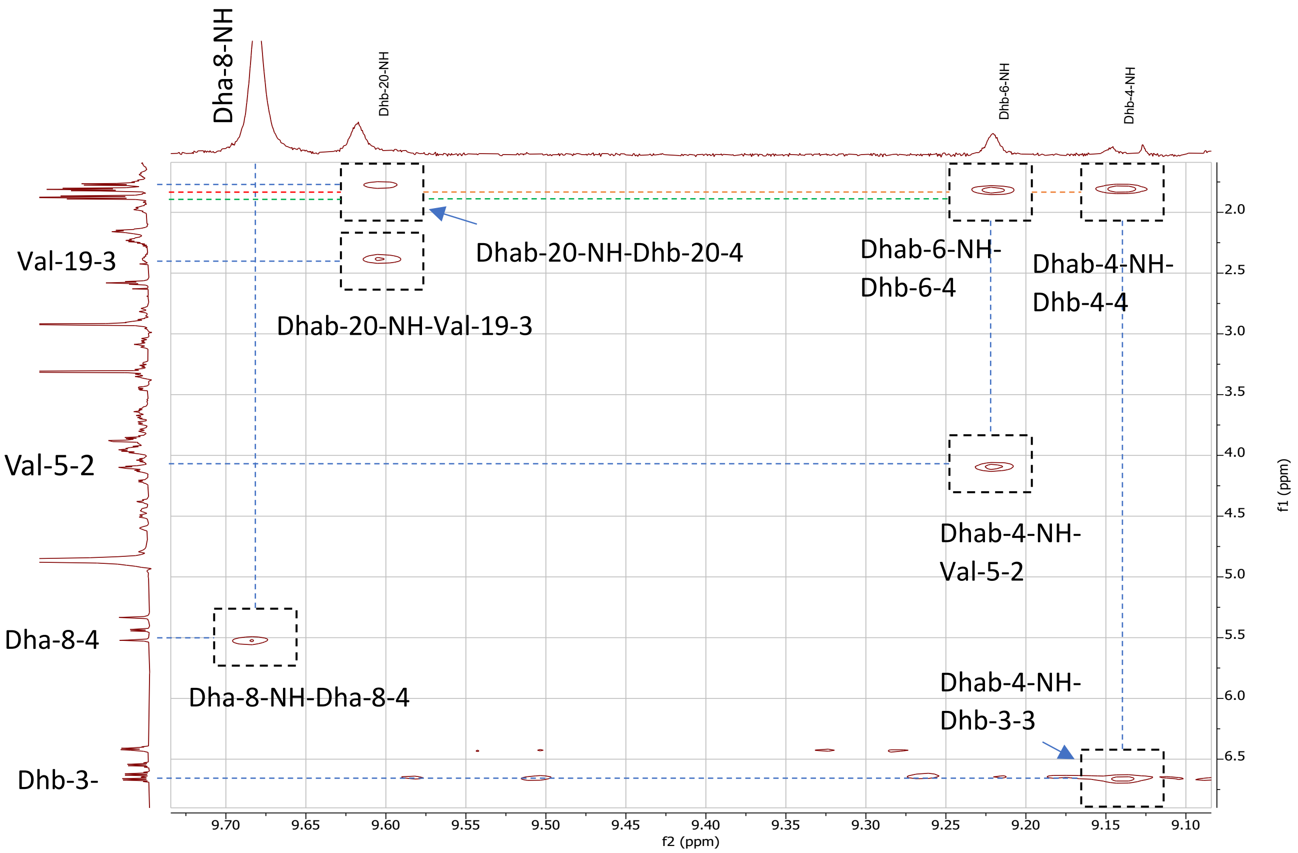

Supplementary Fig. 9. Key ROESY correlations indicating *Z* configurations of Dhbs 2, 3, 4, and 6.

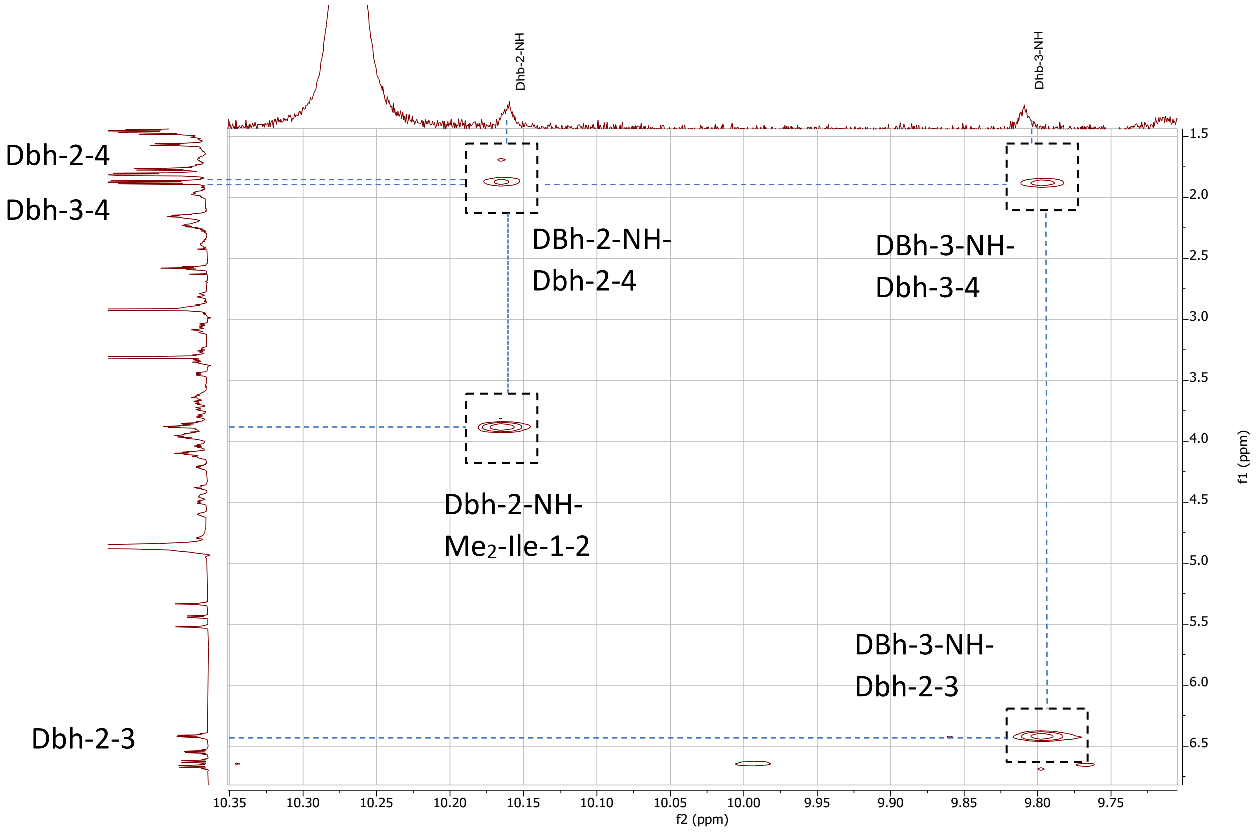

Supplementary Fig. 10. Key correlations in the ROESY spectra in support of Supplementary Figure 3.

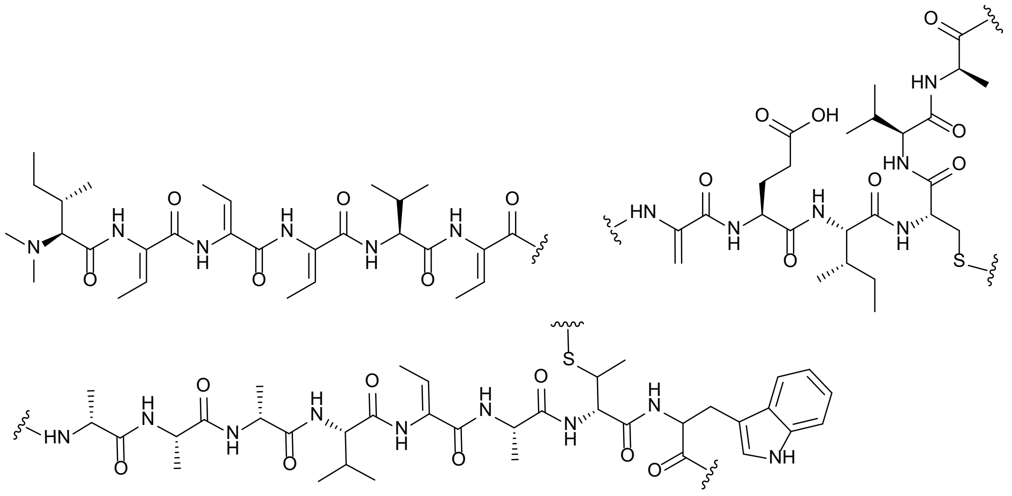

Supplementary Fig. 11. Three key substructures based on detail analysis of Hα-NH (i, i+1) ROESY correlations and NH to -CO, Hα to -CO HMBC cross peaks between adjacent amino acids allowed partial determination of peptide sequence.

The fragments were unambiguously assigned by NMR and these were: (NMe_2Ile-Dhb-Dhb-Dhb-Val-Dhb), Dha-Glu-Ile-Ala(S_1)-Val-Ala, Ala-Ala-Ala-Val-Dhb-Ala-Abu(S_2)-Trp.

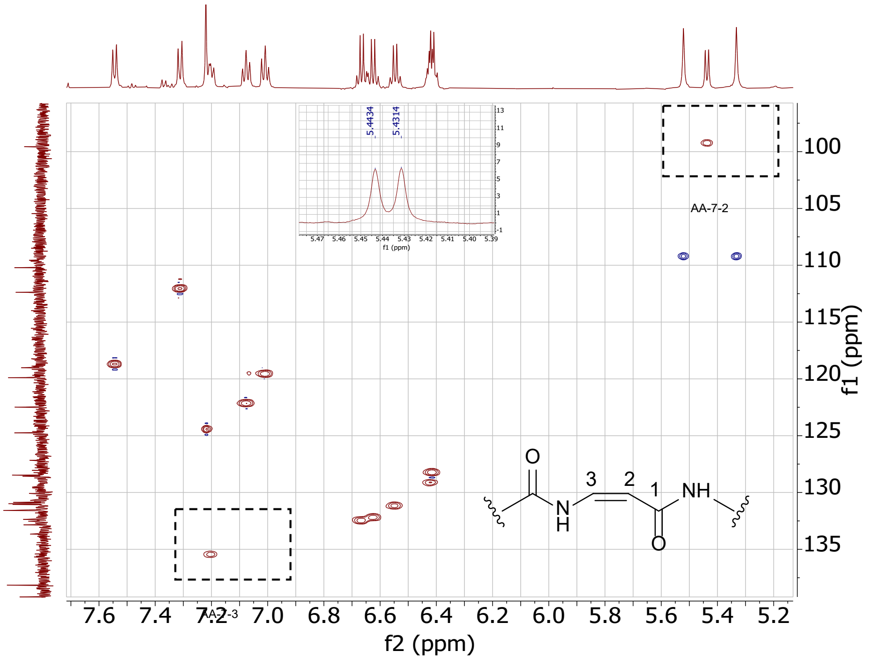

Supplementary Fig. 12. HSQC chemical shifts for Aaa-7. Inset, ^{1}H splitting of 8.4 Hz indicating *cis*-geometry for Aaa-7.

An unprecedented β-enamino acid, (*cis*)-3-amino-acrylic acid (Aaa) was found to be present in **7** based on characteristic NMR ^{1}H coupling constants and chemical shifts in the HSQC.

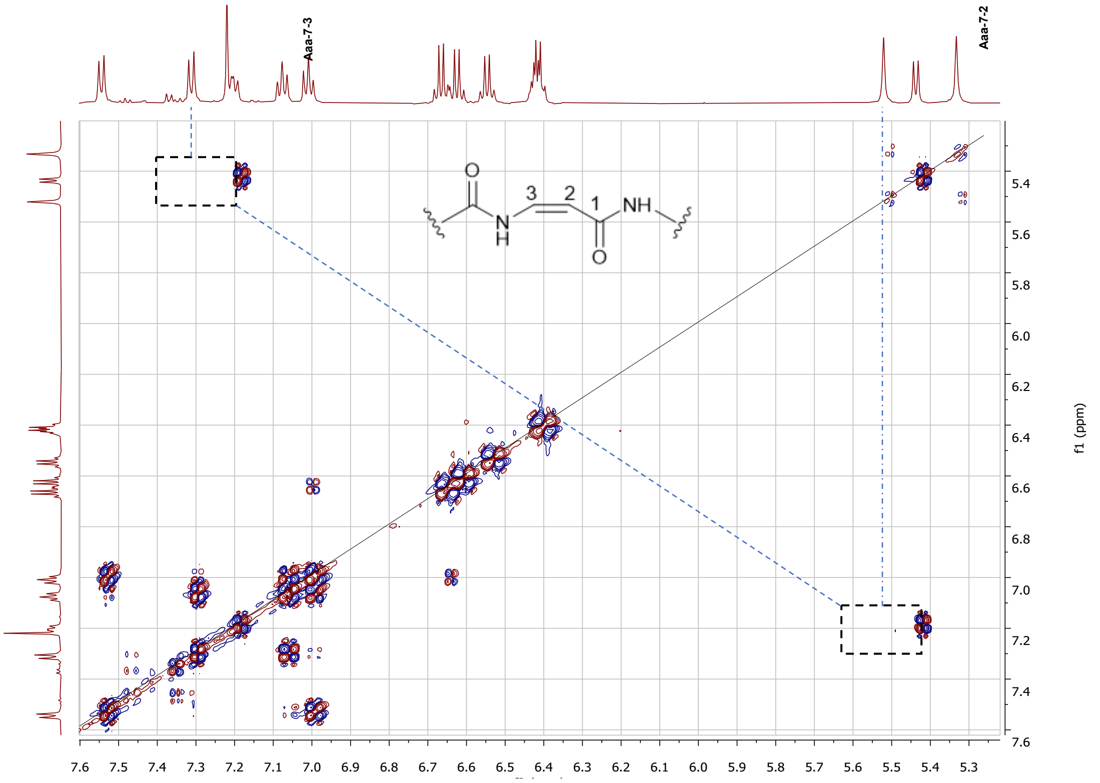

Supplementary Fig. 13. Key COSY correlation within Aaa-7 subunit.

An unprecedented unsaturated β-enamino acid, (*cis*)-3-amino-acrylic acid (Aaa) was found to be present in **7** based on cross peaks in COSY.

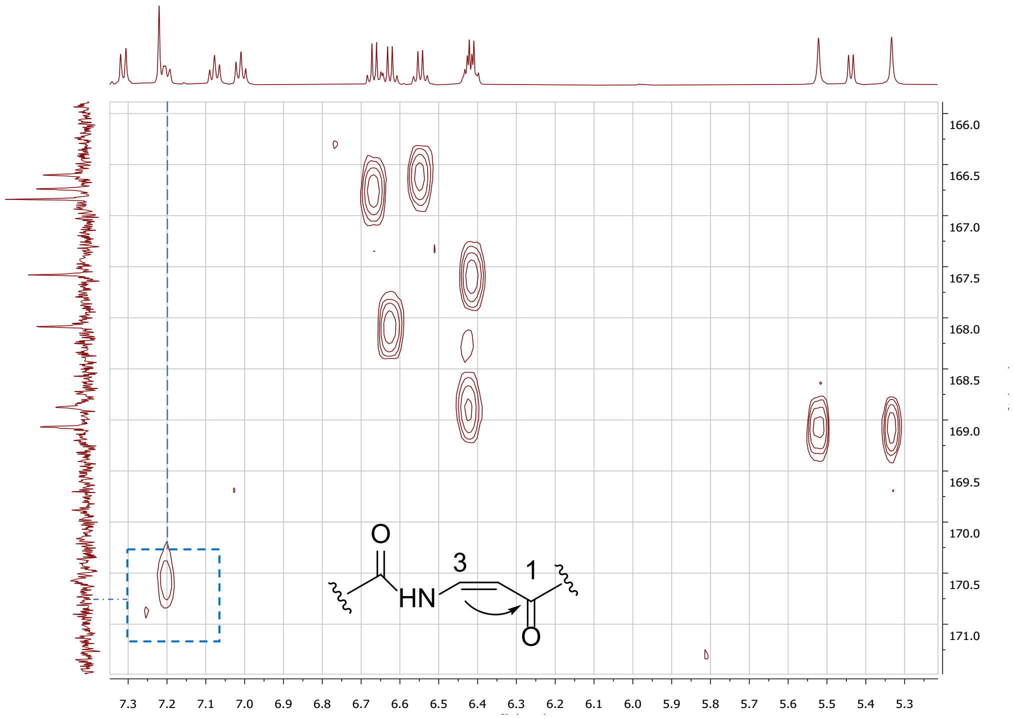

Supplementary Fig. 14. Key HMBC Correlation within Aaa-7.

An unprecedented unsaturated β-enamino acid, (*cis*)-3-amino-acrylic acid (Aaa) was found to be present in **7** based on HMBC.

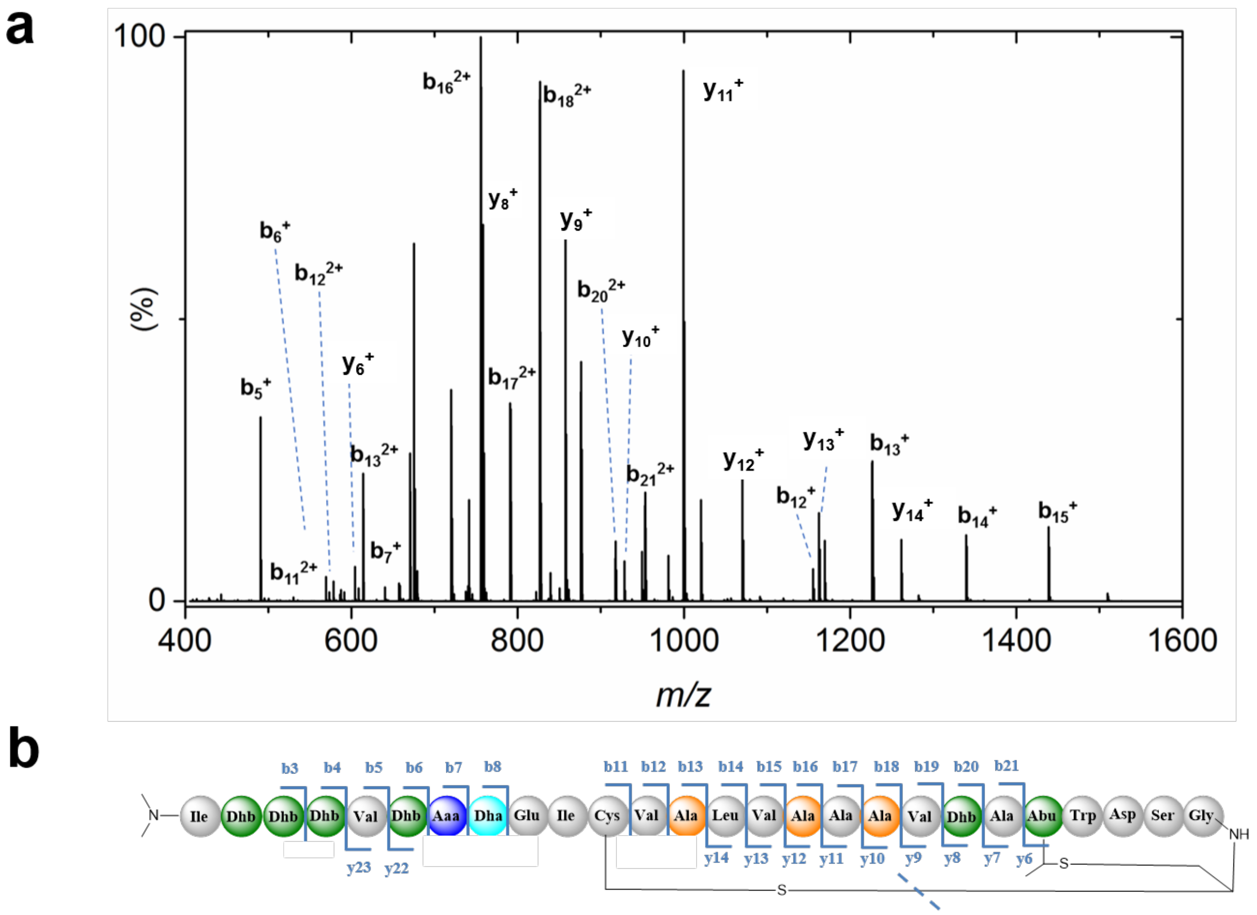

Supplementary Fig. 15. High Resolution *De Novo* Tandem MS analysis of kintamdin **7.**

The $[M+3H]^{3+}$ ion at *m/z* 836.7524 was analysed by MS^2 and pseudo MS^3 by FT-ICR MS. (**a**). A typical MS2 spectrum is shown with assigned fragments annotated. (**b**). The overall sequence coverage from combined fragmentation experiments is shown. Assigned fragments are also shown in Supplementary Table S1.

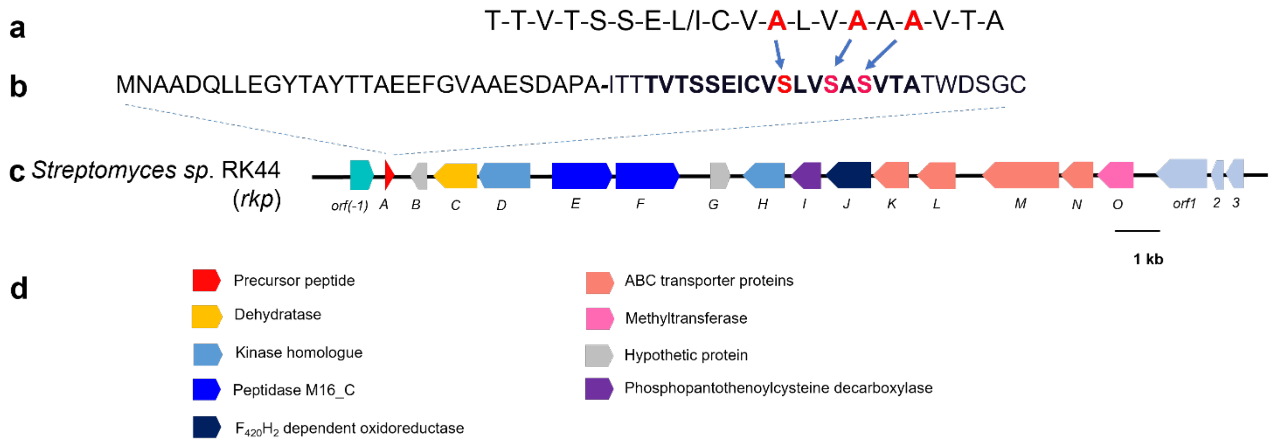

Supplementary Fig. 16. The putative BGC responsible for the kintamdin production in *Streptomyces* sp. RK44.

a. The sequence tag generated from NMR and tandem MS analysis. **b**. the sequence of the precursor peptide (KinA). Three of Ser amino acids in the genomic sequence (Ser-13, 16 and 18, highlighted in red) was present in the final structure as Ala residues highlighted in red. **c**. the arrangement of the *kin* BGC. **d**. The biosynthetic genes in the *kin* BGC with assigned functions.

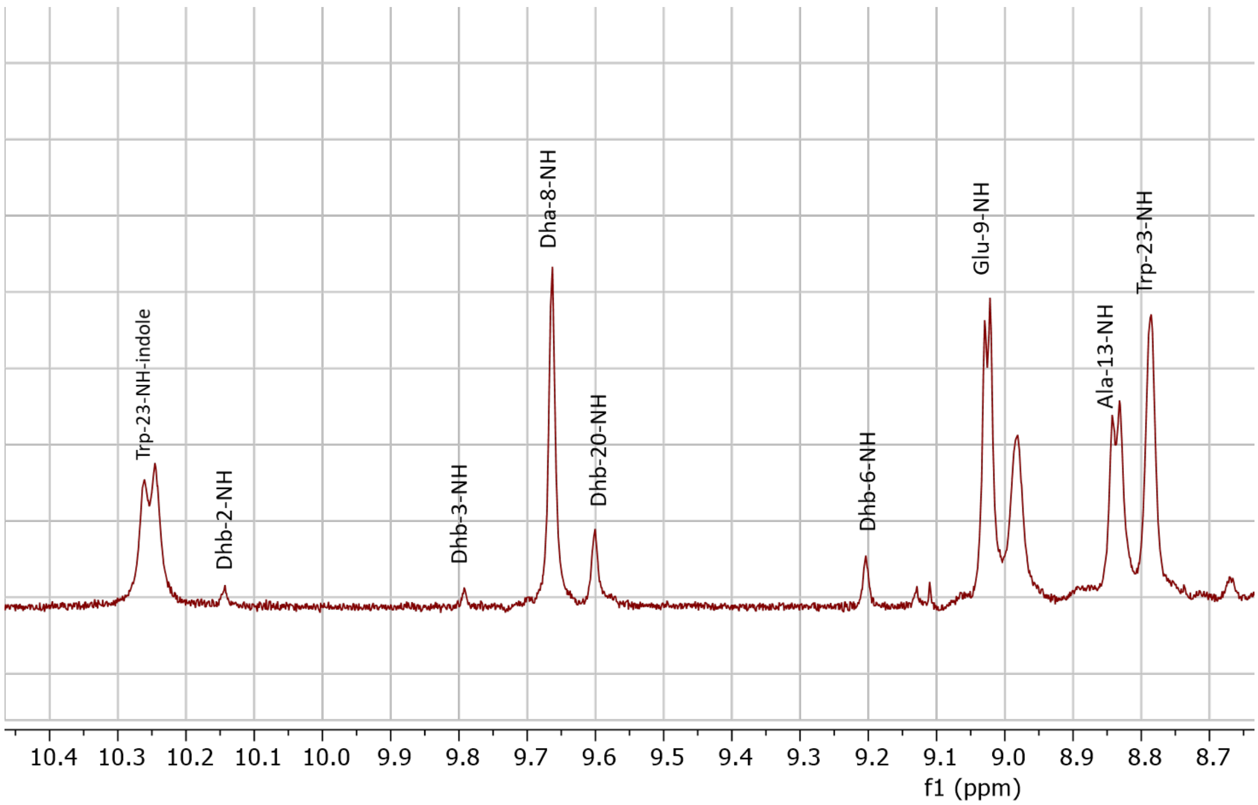

Supplementary Fig. 17. Expanded and annotated ^{1}H NMR spectrum of **7**.

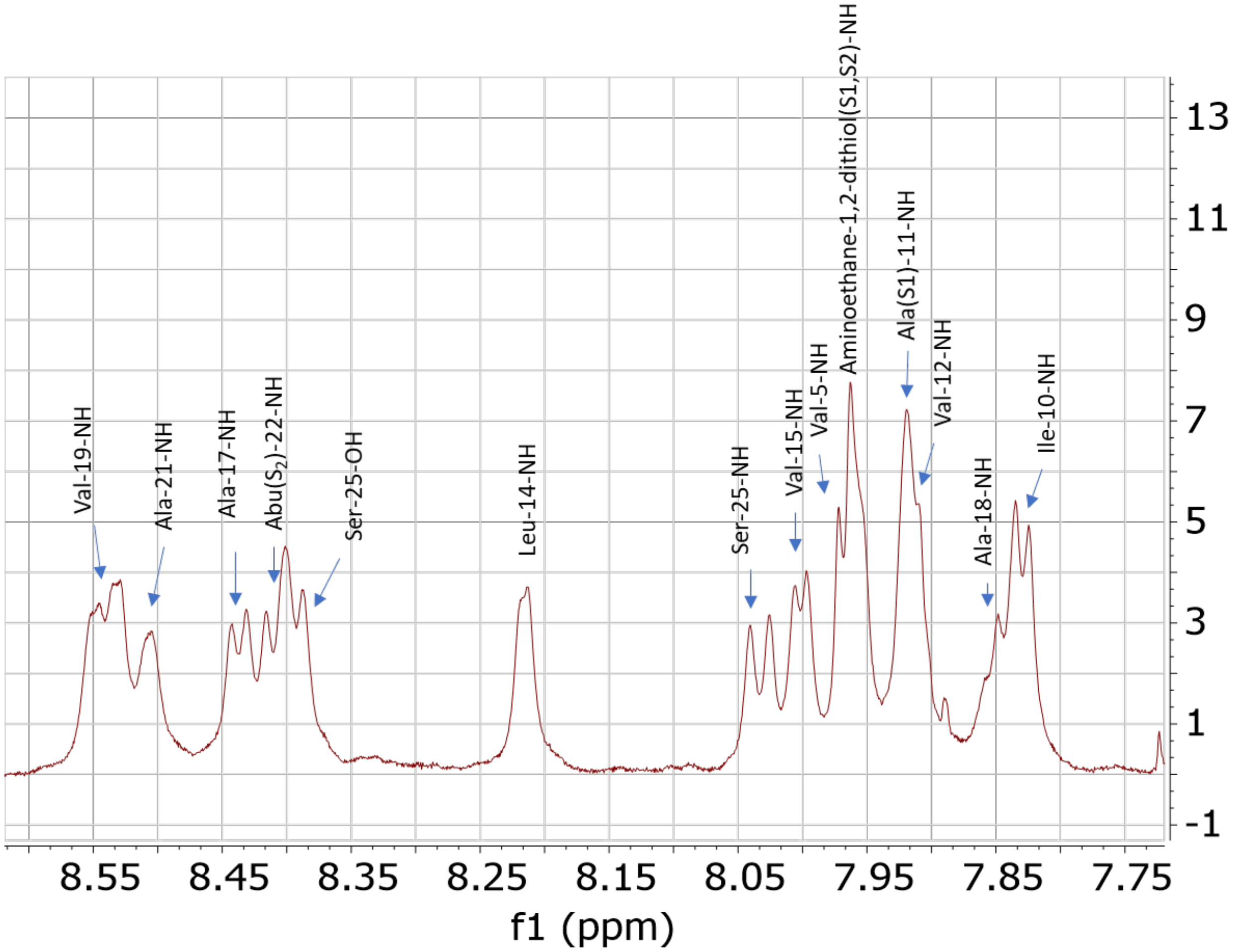

Supplementary Fig. 18. Expanded and annotated ^{1}H NMR spectrum of **7**.

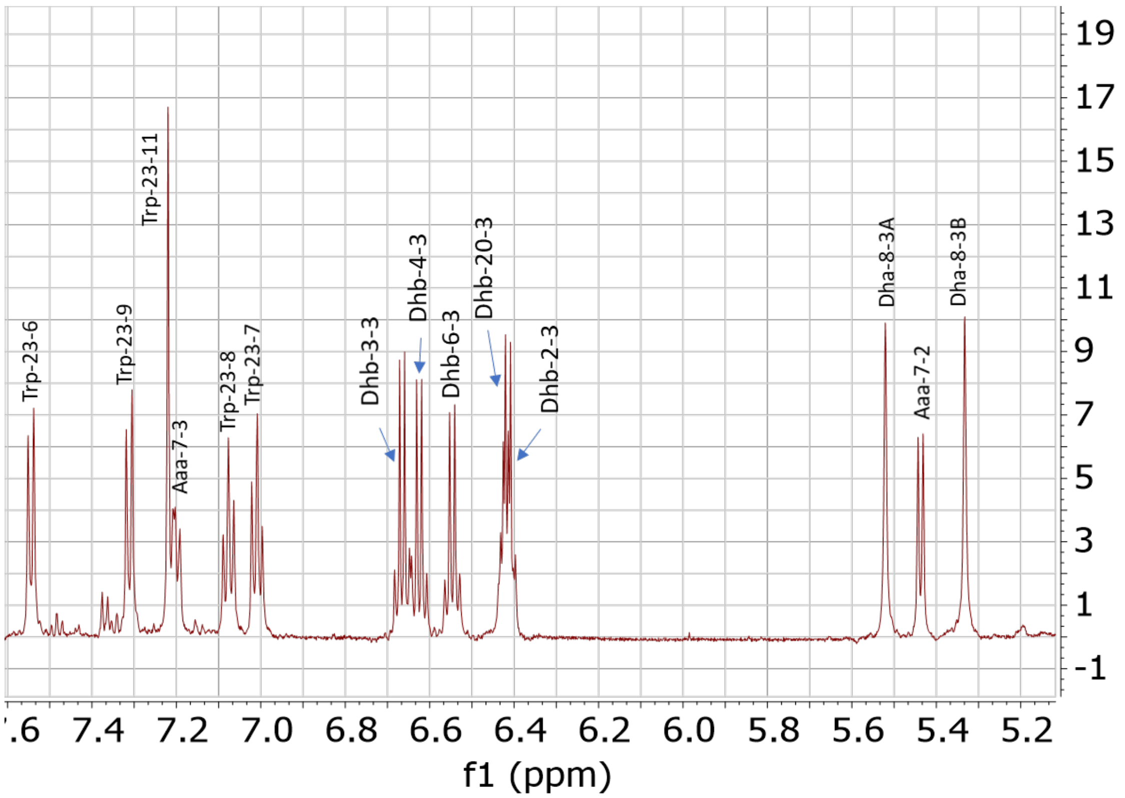

Supplementary Fig. 19. Expanded and annotated ^{1}H NMR spectrum of **7**.

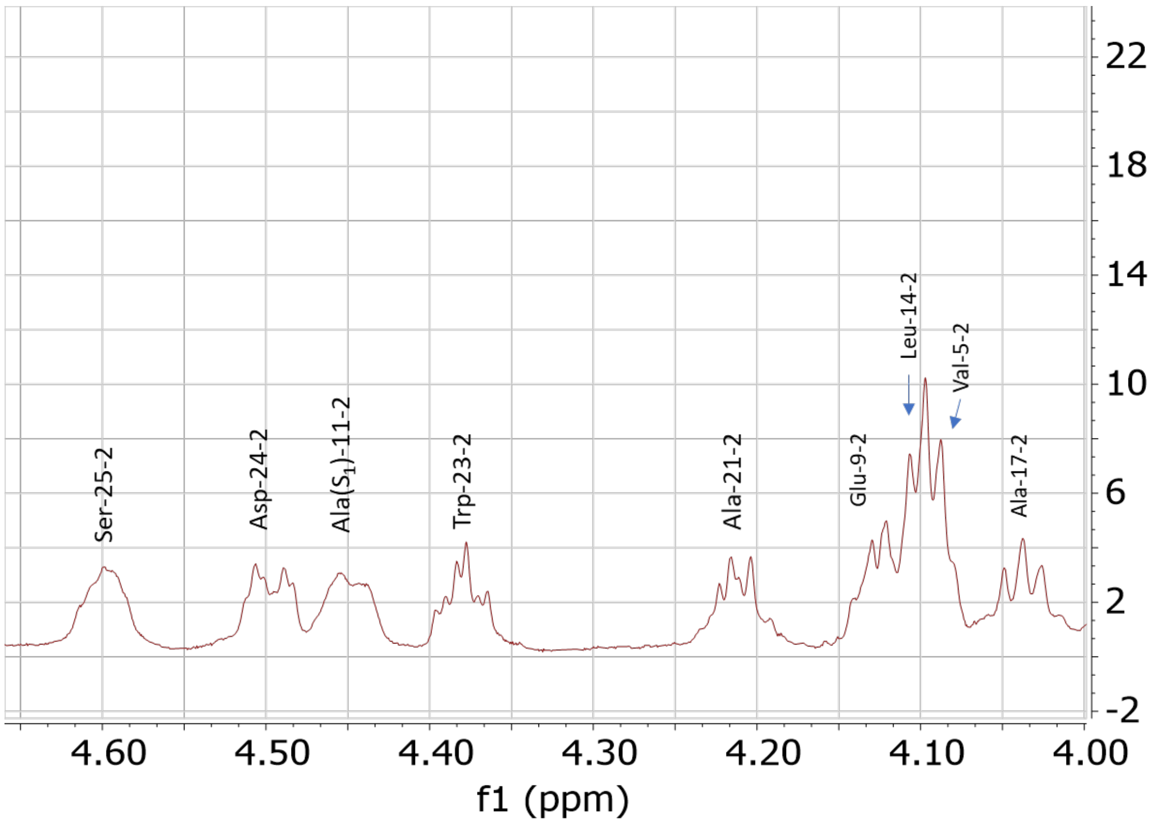

Supplementary Fig. 20. Expanded and annotated ^{1}H NMR spectrum of **7**.

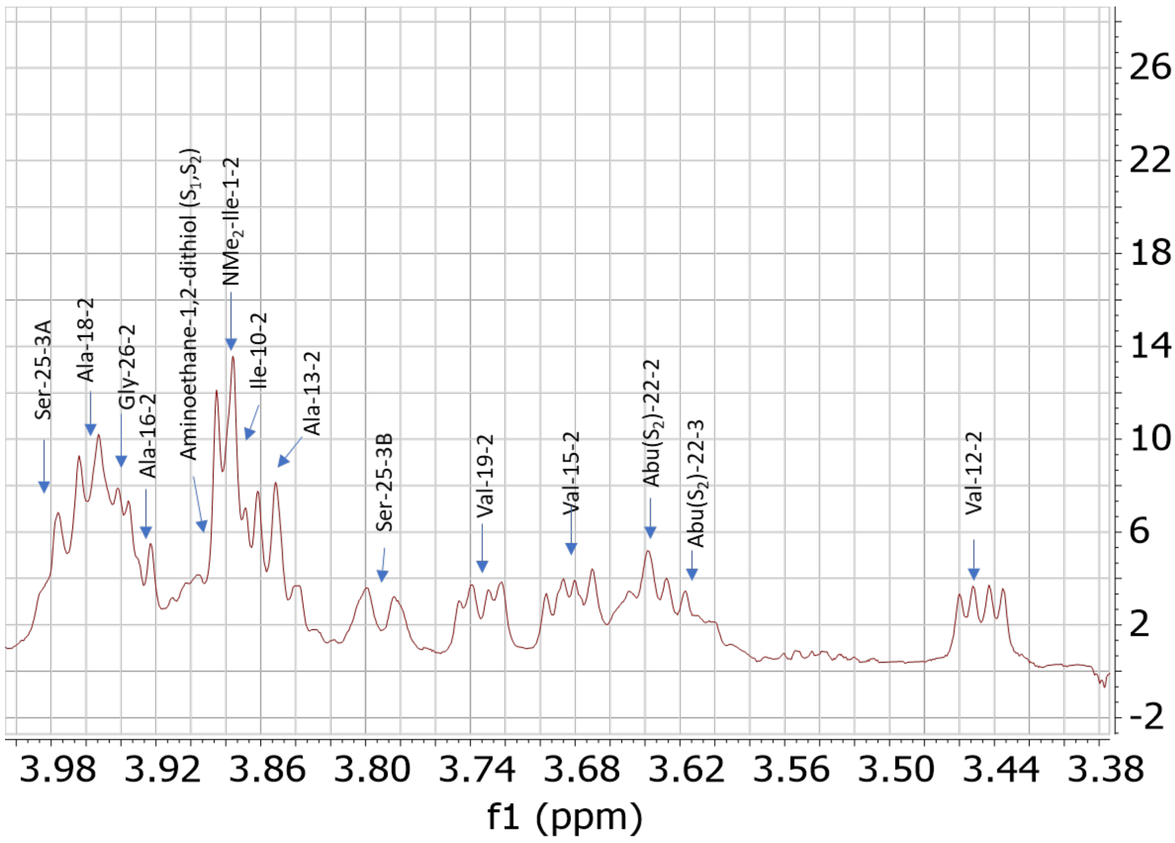

Supplementary Fig. 21. Expanded and annotated ^{1}H NMR spectrum of **7**.

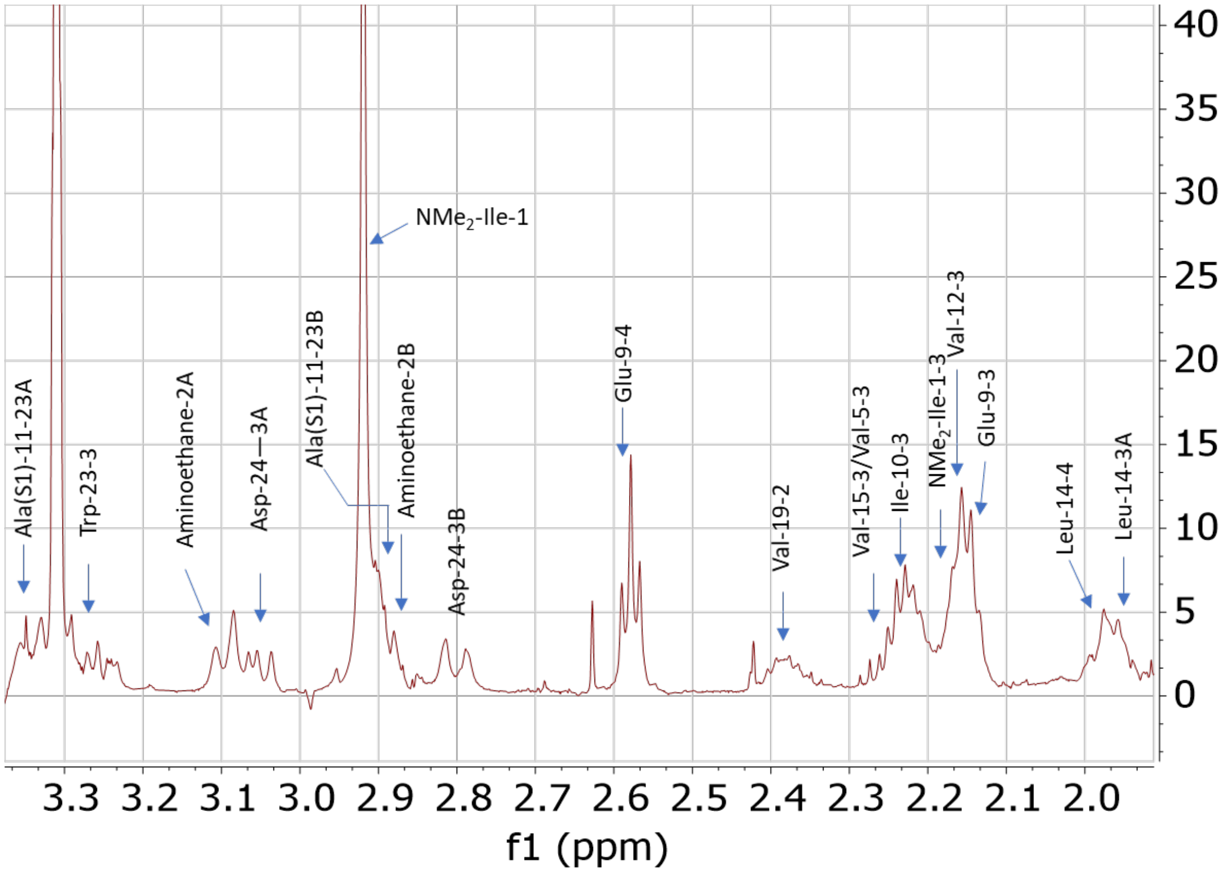

Supplementary Fig. 22. Expanded and annotated ^{1}H NMR spectrum of **7**.

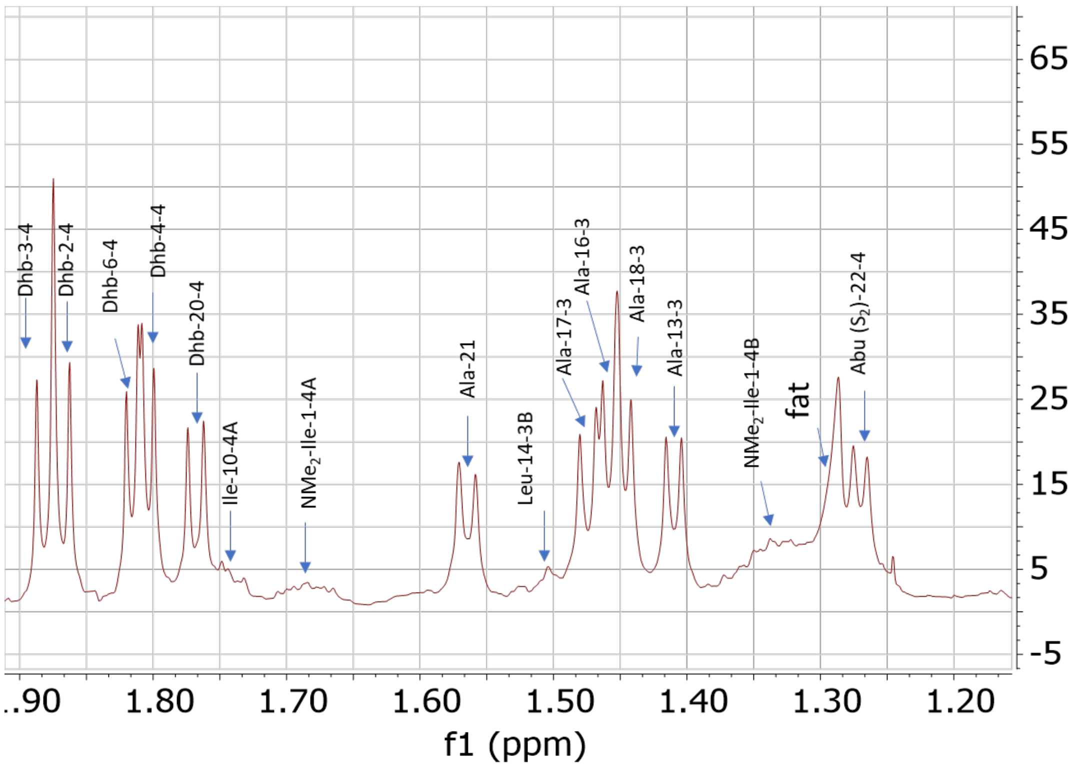

Supplementary Fig. 23. Expanded and annotated ^{1}H NMR spectrum of **7**.

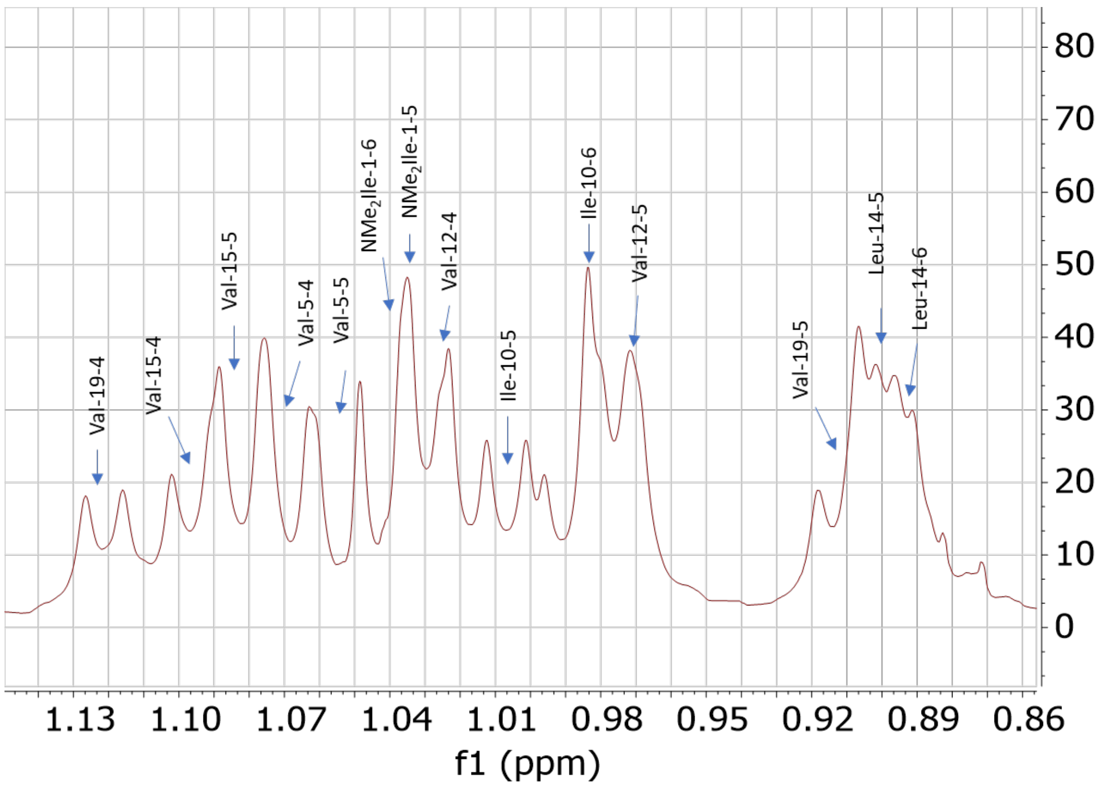

Supplementary Fig. 24. Expanded and annotated ^{1}H NMR spectrum of **7**.

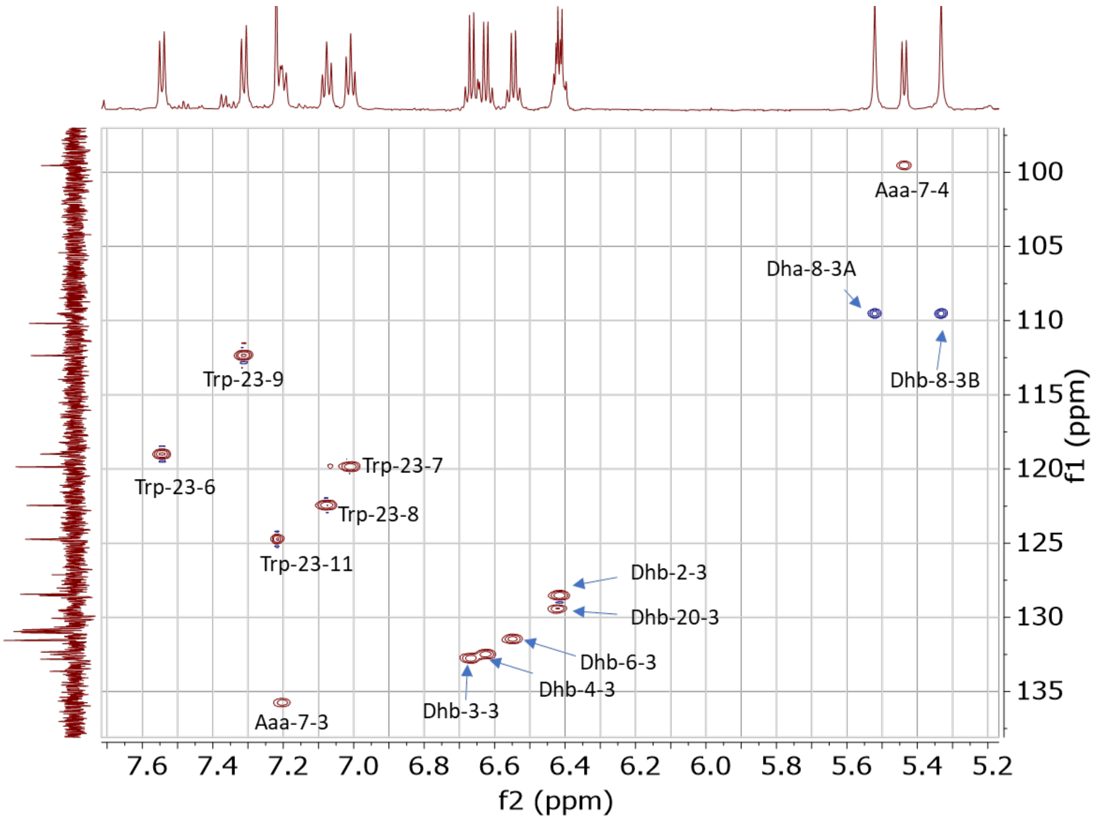

Supplementary Fig. 25. Expanded and annotated HSQC spectrum of **7**.

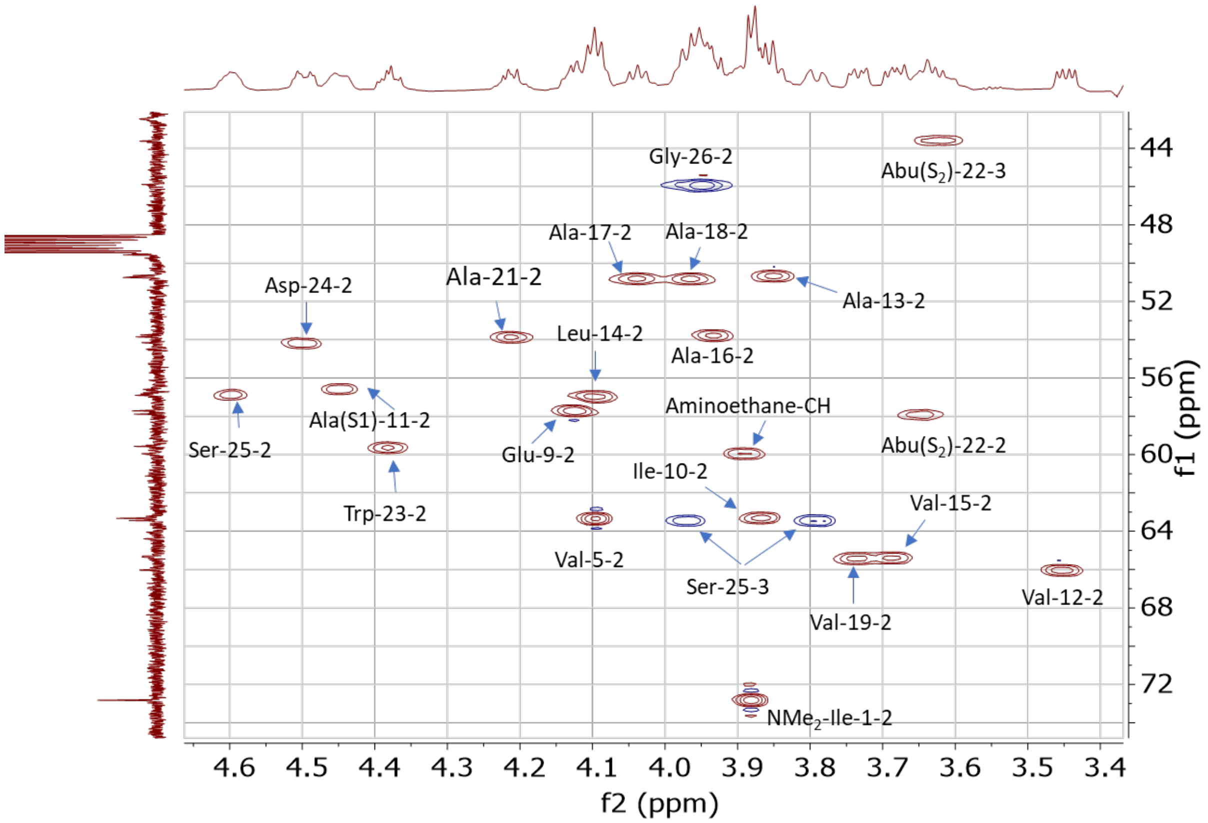

Supplementary Fig. 26. Expanded and annotated HSQC spectrum of **7**.

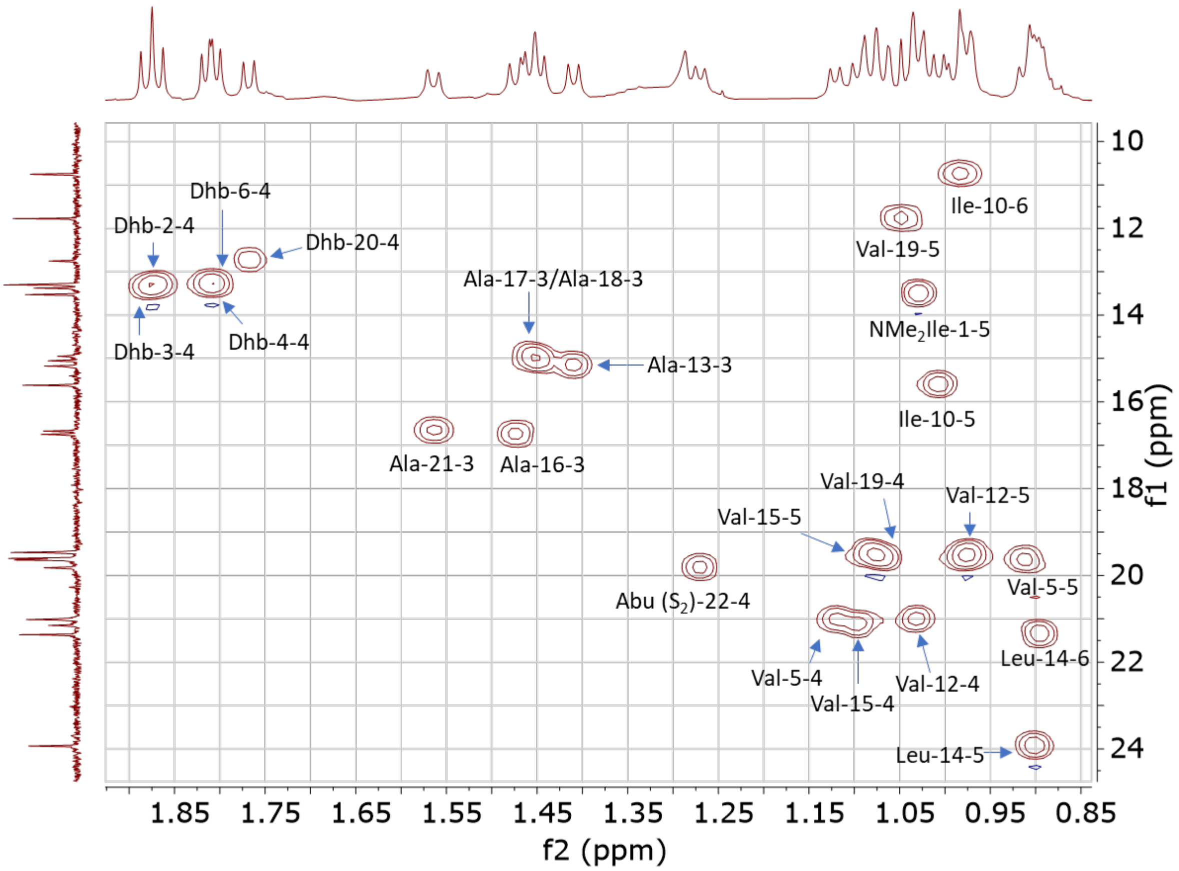

Supplementary Fig. 27. Annotated HSQC spectrum of **7**.

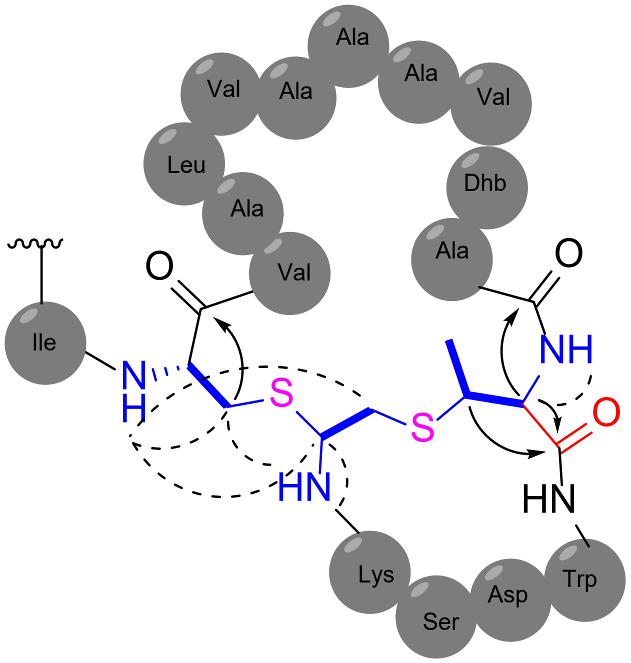

Supplementary Fig. 28. Key cross-peaks in the COSY, HMBC, and ROESY spectra of the *bis*-thioethane unit of **7**, indicating an unprecendented *bis*-thioether cross link has been found to be present in **7**.

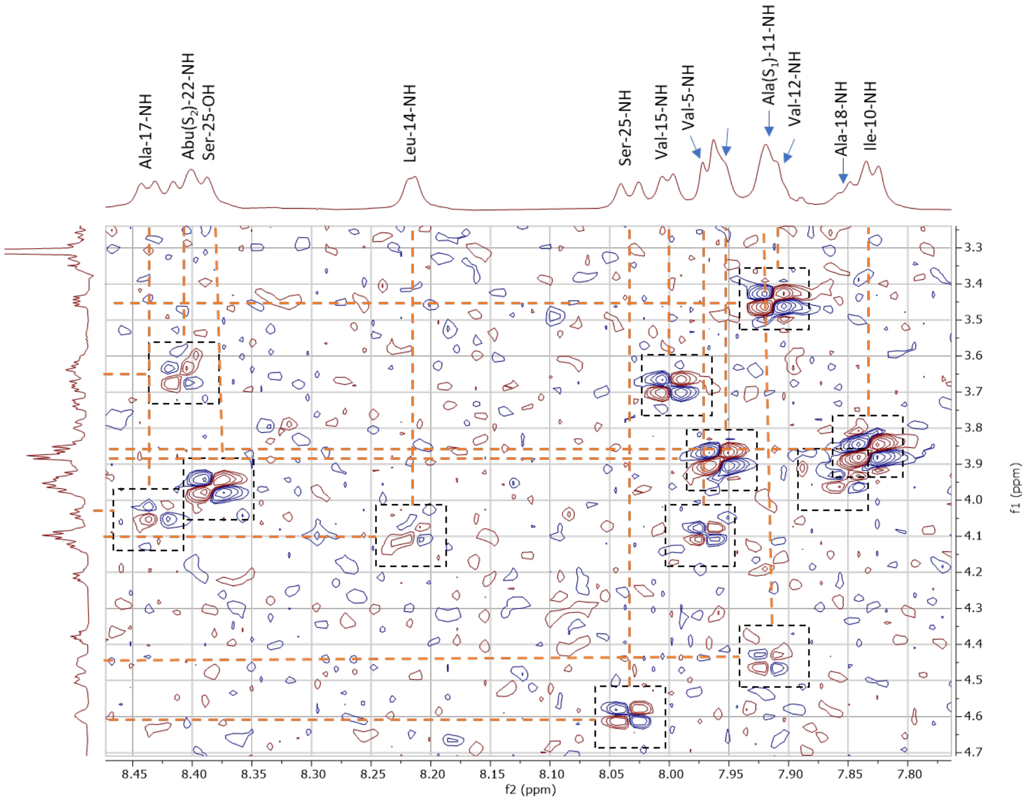

Supplementary Fig. 29. Assignment of NHs in key amino acid residues using COSY correlations between α to NH of amino acid residue.

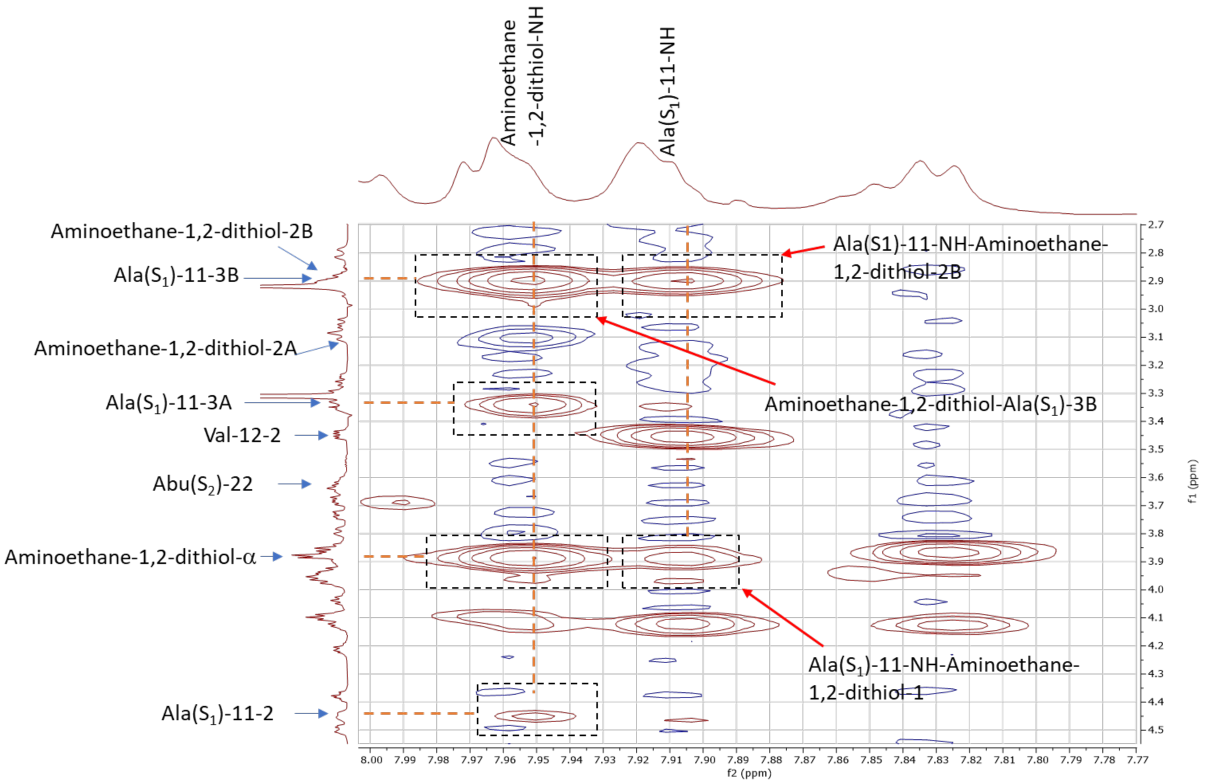

Supplementary Fig. 30. Key correlations in the ROESY spectrum showing link between Ala(S1)-11 and aminoethane-1,2-dithiol group.

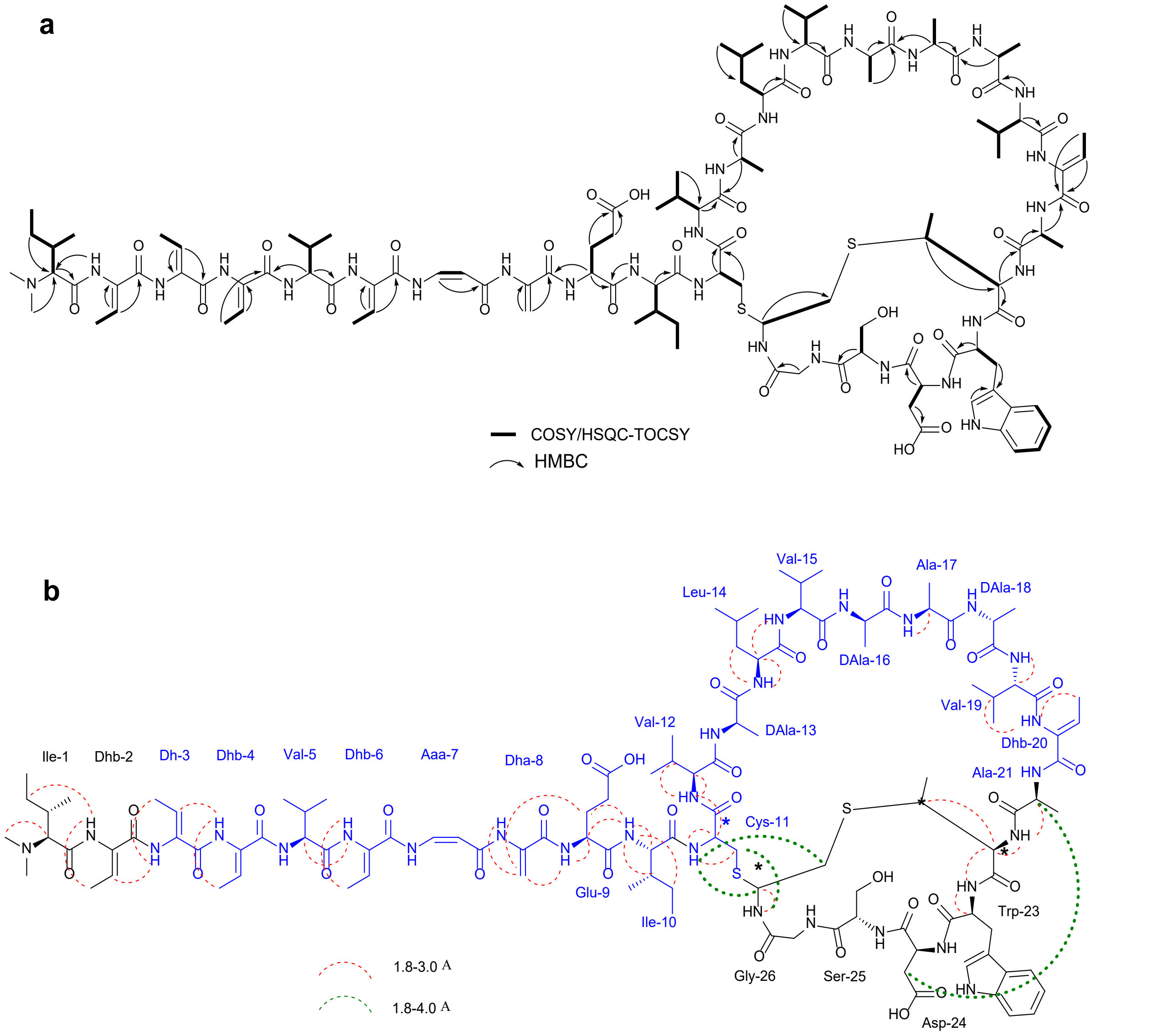

Supplementary Fig. 31. The planar structure of **7**.

a. COSY/HSQC-TOCSY, HMBC (H→C) observed correlations of **7**. **b**. Key local NOE correlations (short and medium range) of **7** indicated in red and blue dash lines which represent the estimated NOE distances obtained from analysis of the NOE spectrum.

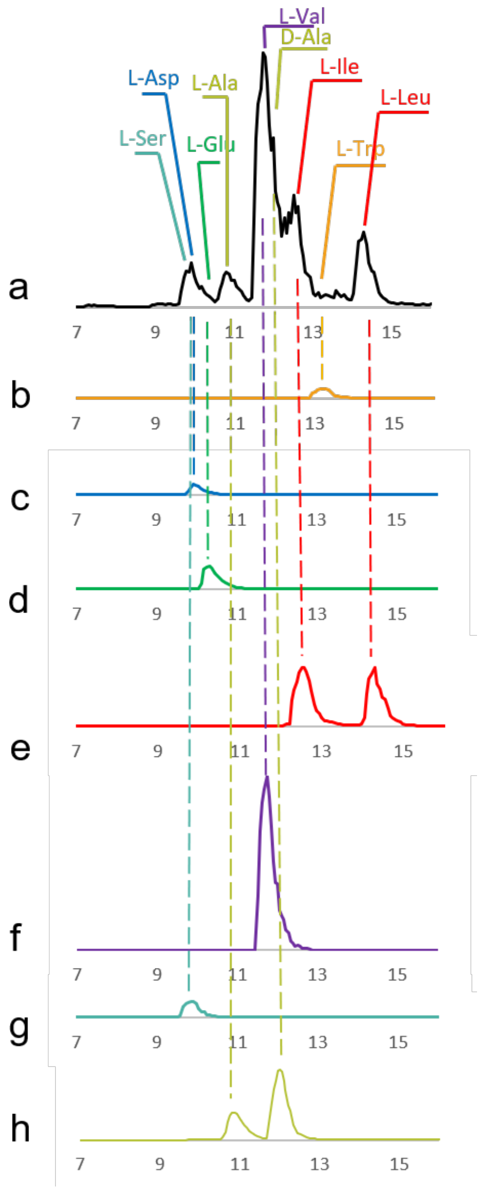

Supplementary Fig. 32. **a**. EIC chromatography of proteinogenic amino acids residues from kintamdin from advanced Marfey's derivatization.

b-h. EIC chromatography of individual derivatized proteinogenic amino acid residues, Trp (b), Asp (c), Glu (d), Ile and Leu (e), Val (f), Ser (g) and Ala (h), respectively.

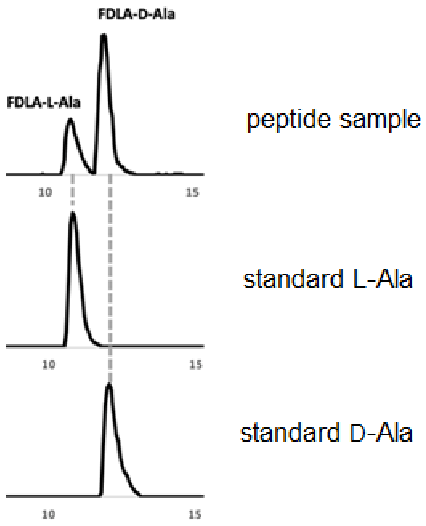

Supplementary Fig. 33. Extracted Ion Chromatogram (EIC) chromatography of derivatized Ala residues from **7** (top), standard L-Ala (middle) and standard D-Ala.

The ratio of derivatized L-Ala and D-Ala from **1** is estimated to be 1:3, indicating three D-Ala residues in **7**.

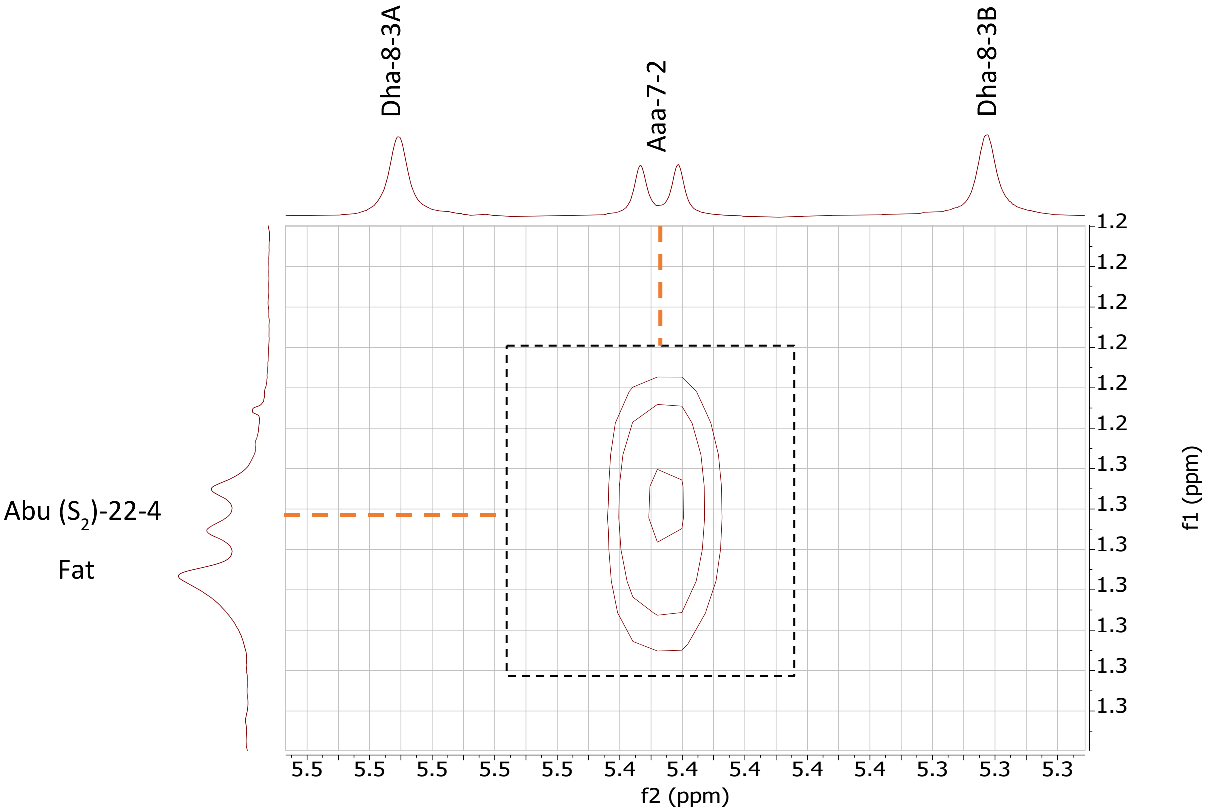

Supplementary Fig. 34. Observed ROE correlation between Aaa-7-2 (αH) and the methyl group of Abu(S_2)-22-4.

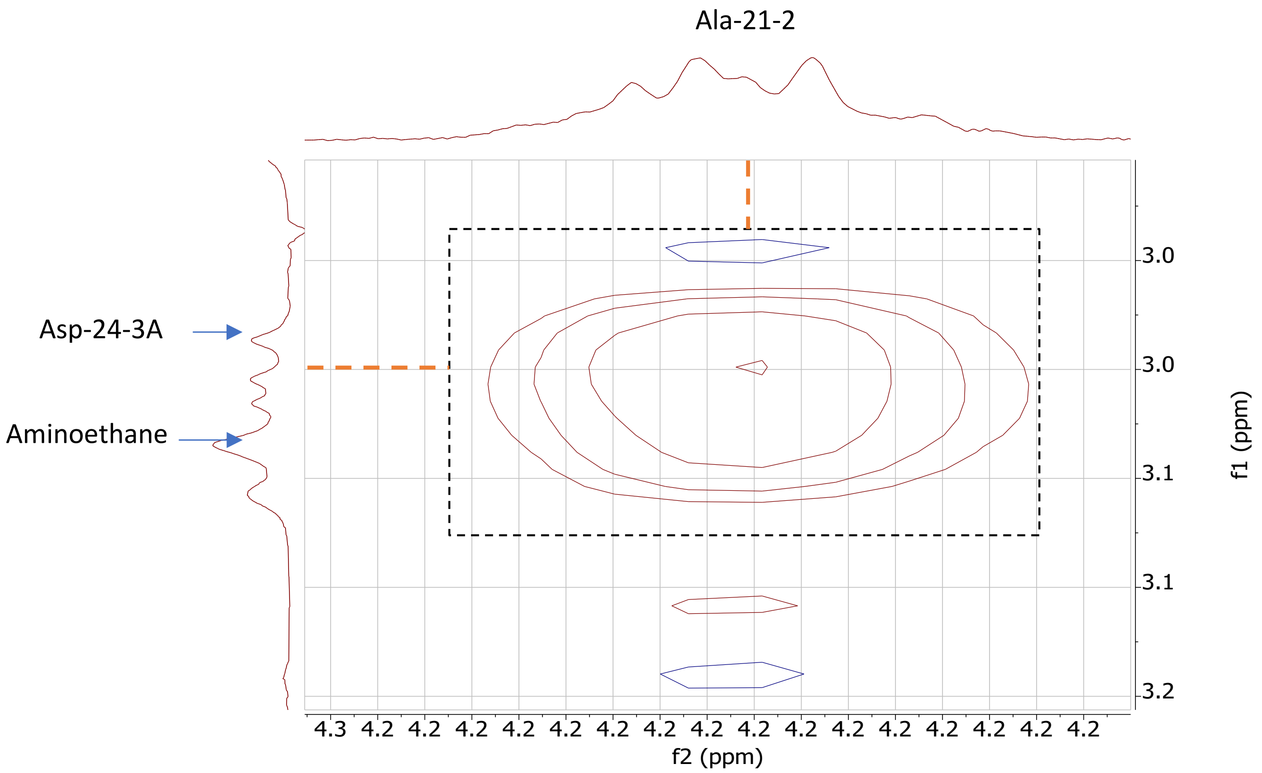

Supplementary Fig. 35. Observed ROE correlation between Ala-21-2 (αH) and Asp-24-3A (βH).

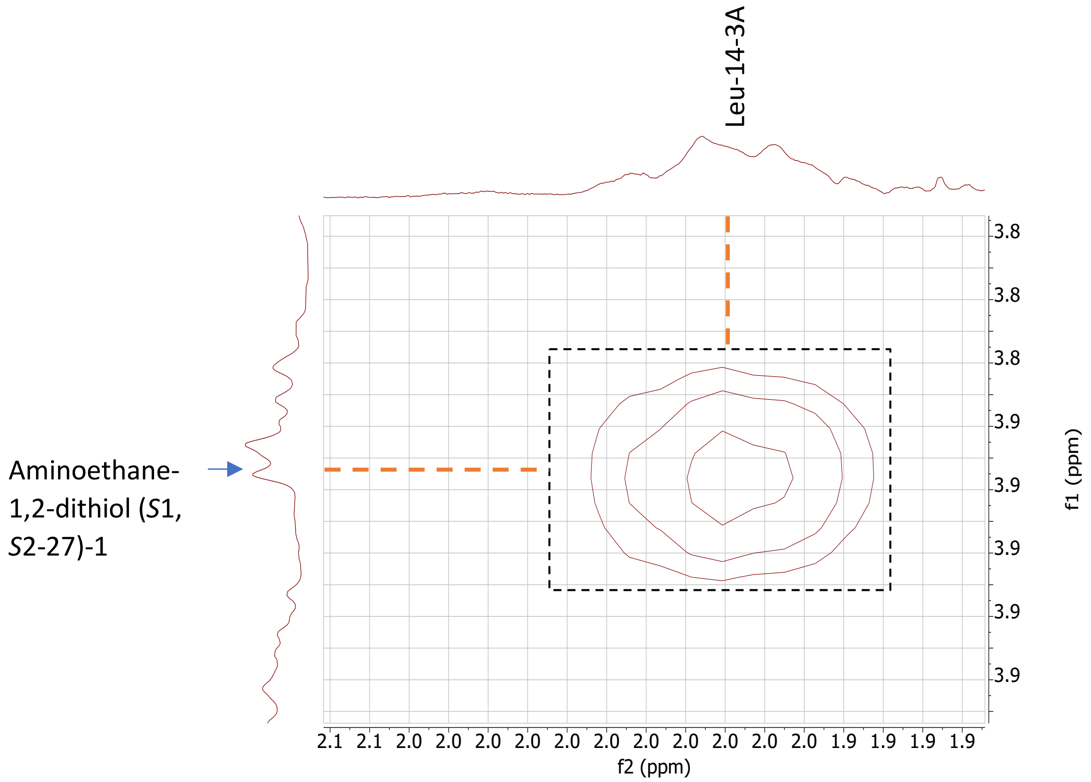

Supplementary Fig. 36. Observed ROE correlation between Aminoethane-1,2-dithiol(*S*1, *S*2)-27-1 (αH) and Leu-14-3A (βH).

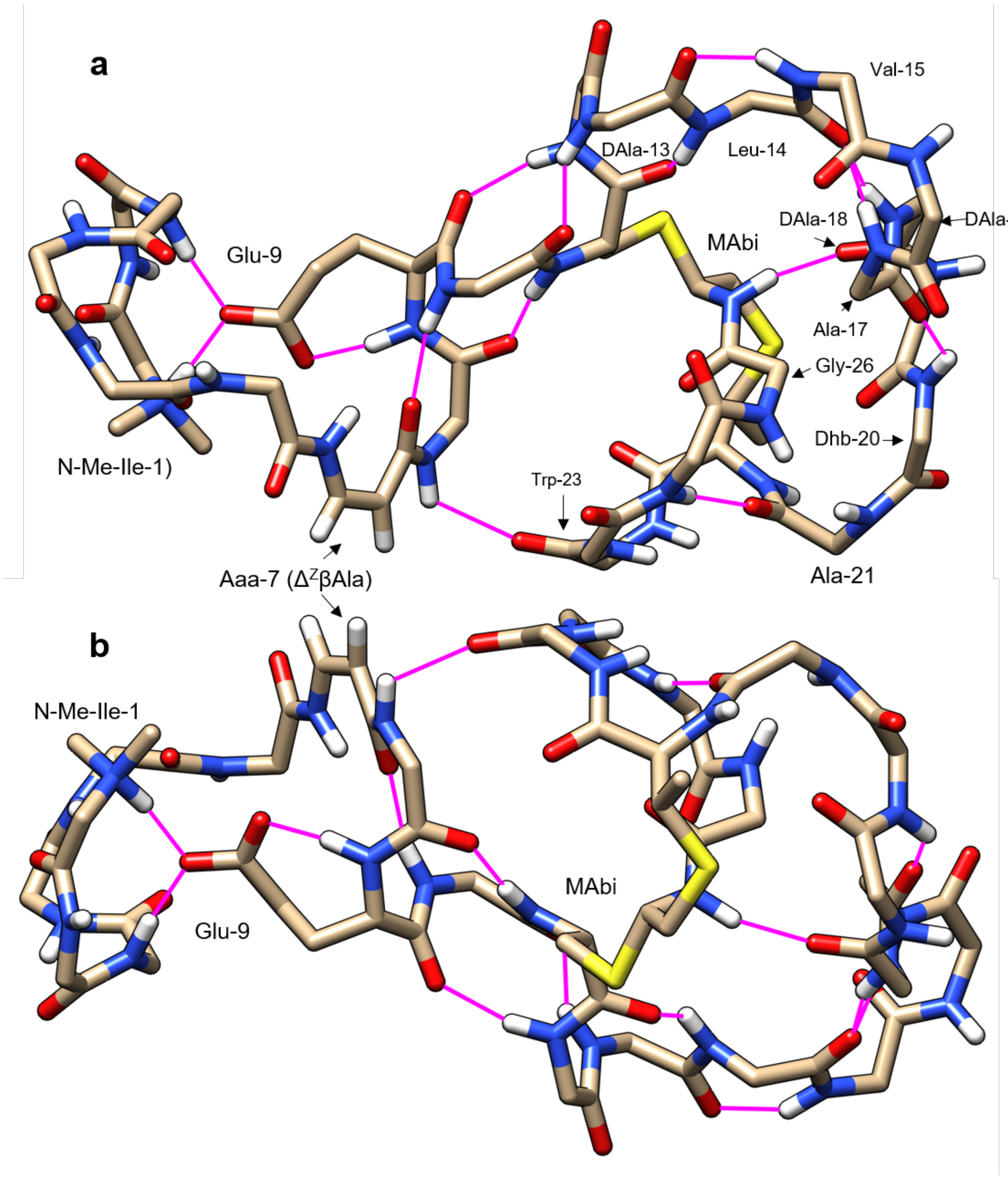

Supplementary Fig. 37. Snapshots of **7** from AIMD simulation.

Front (**a**) and rear (**b**) views showing the cage-like C-terminal region and coiling of the acyclic N-terminal region with H-bond interactions (pink lines). The MAbi residue was found to be largely buried inside the macrocyclic cage. The structure of the helix-like N-terminal region can be seen to depend on H-bonds between backbone amide hydrogens, the Glu-9 side chain carboxylate and the β-amino acid, Aaa-7. The planarity of Aaa-7 also displays an important role in determining the geometry of the acyclic chain and its interaction with the macrocyclic cage.

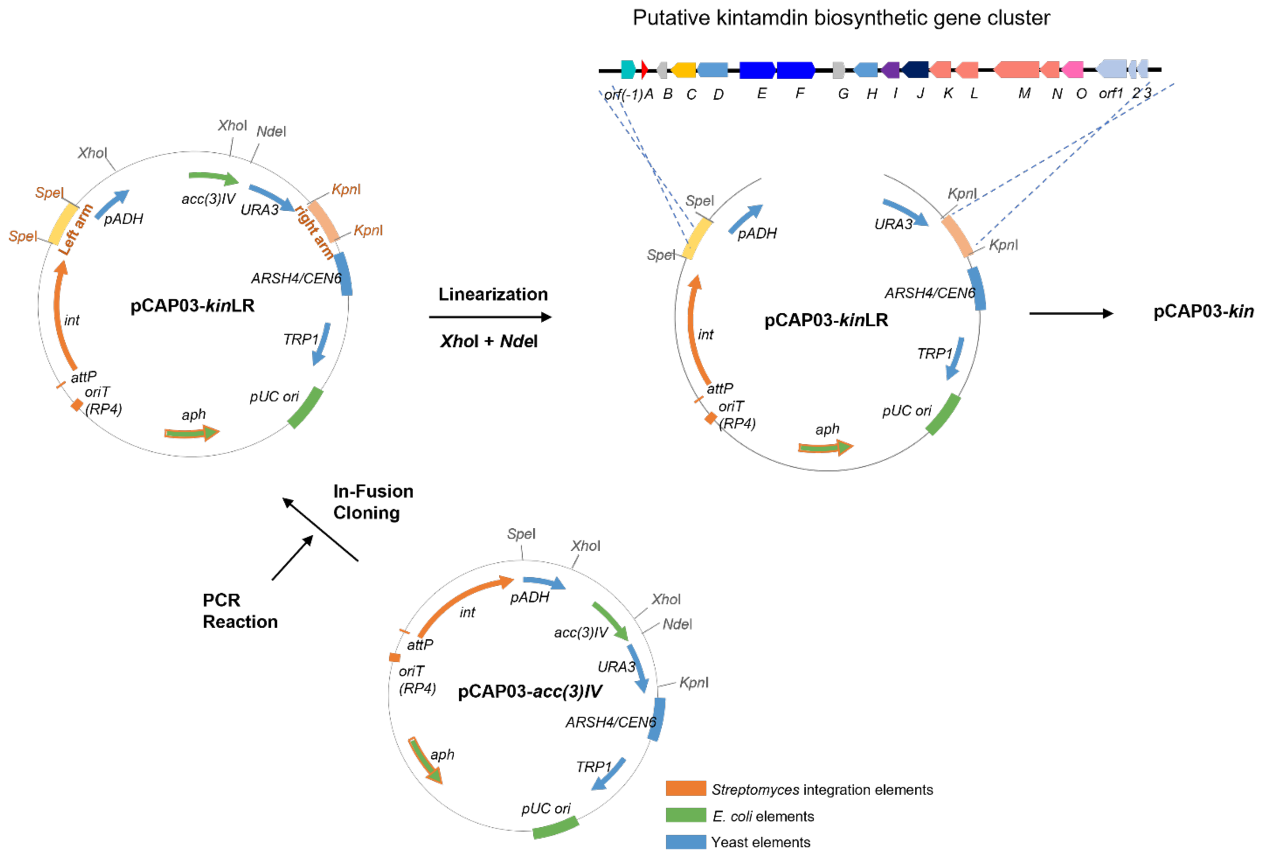

Supplementary Fig. 38. Scheme of a modified TAR cloning strategy by inserting two arms into the original pCAP03-*acc(3)IV* plasmid to generate pCAP03-*kin*LR construct.

To improve the capture efficiency, two 1kb homologous arms that flank the upstream and downstream regions of the BGC of interest were amplified and inserted into the *Spe*I site and the *Kpn*I site of pCAP03-*acc(3)IV*, respectively. In this construction method, the pADH-ura3 counter selection system was kept for counteracting plasmid recircularization in yeast due to nonhomologous end joining (NHEJ).

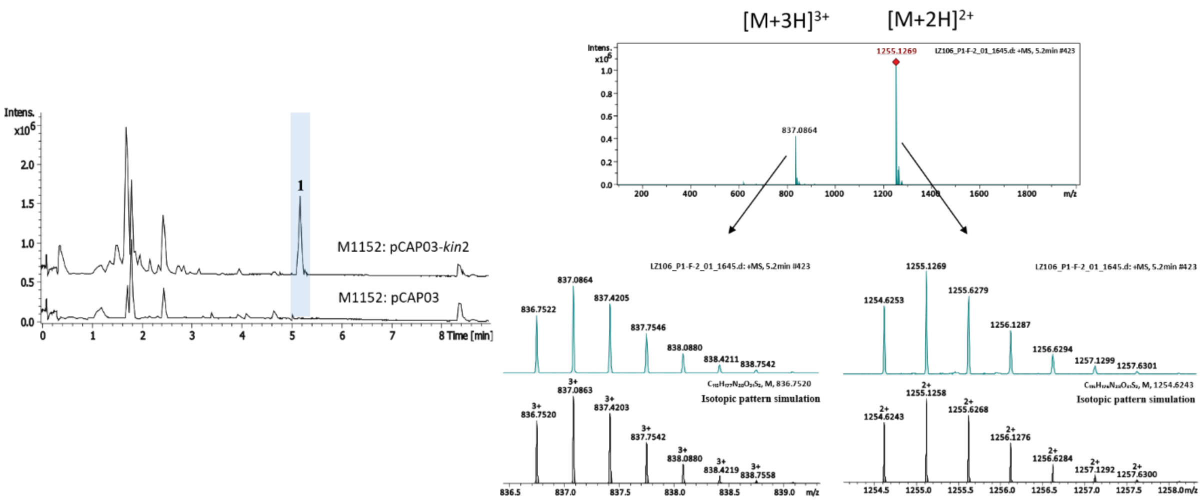

Supplementary Fig. 39. MS analysis of **7** identified from heterologous expression system.

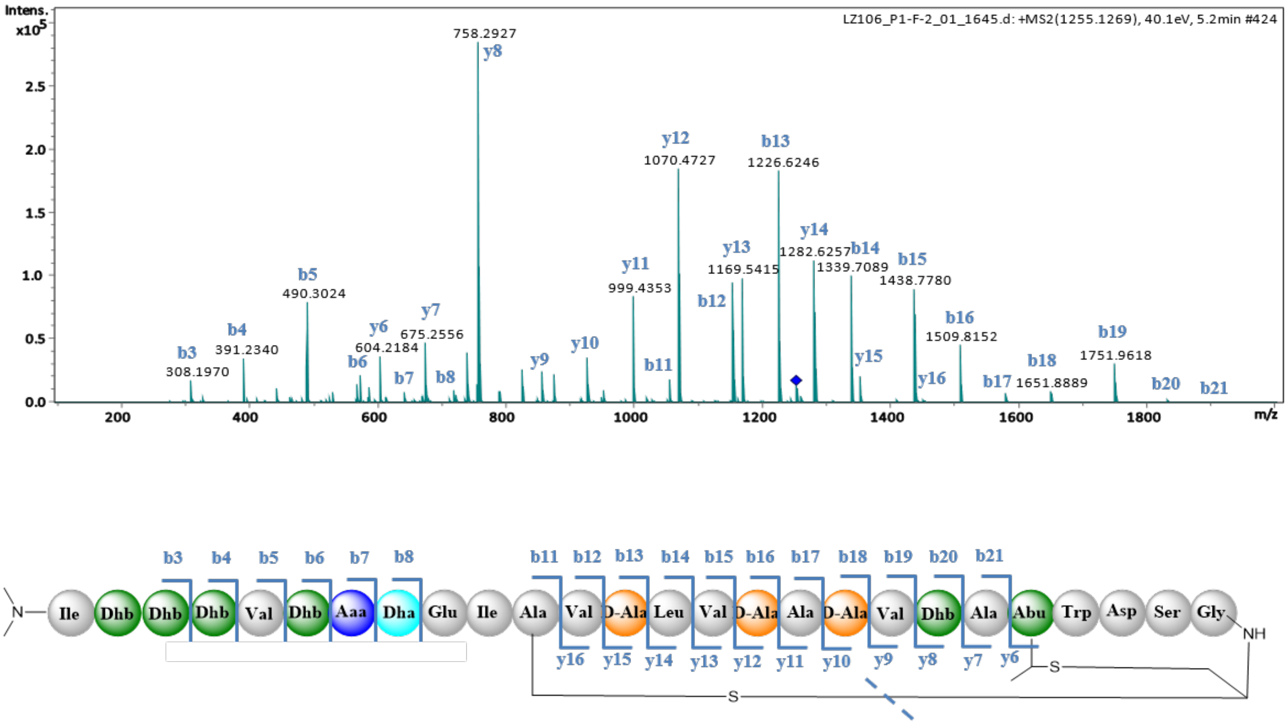

Supplementary Fig. 40. MS^2 fragmentation analysis of **7** identified in the heterologous expression system.

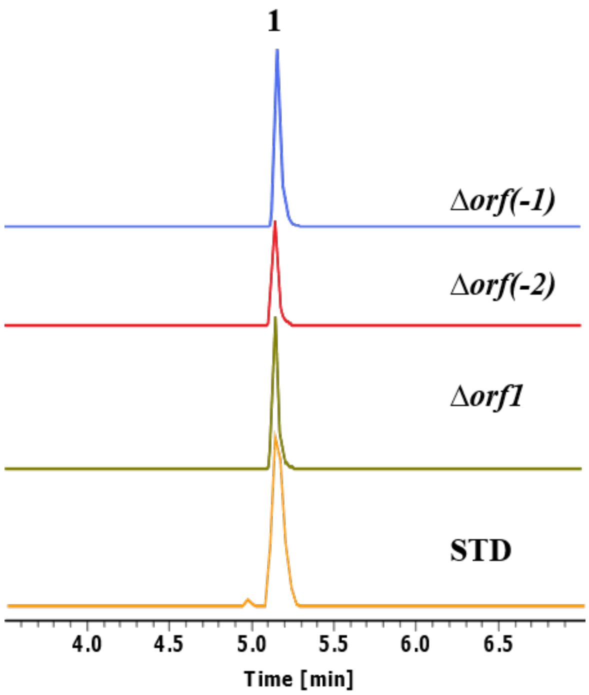

Supplementary Fig. 41. EIC extraction of **7** in the variants of the heterologous expression systems.

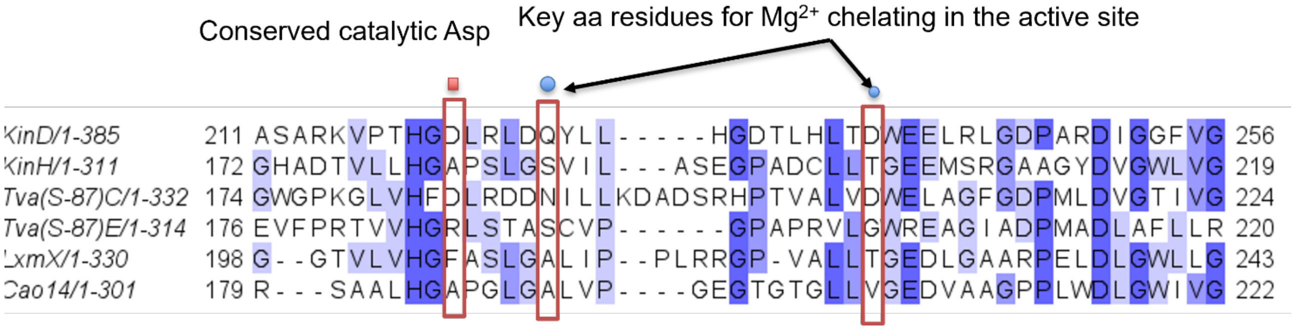

Supplementary Fig 42. Sequence comparison of KinD, KinH, LxmX and Cao14 with two kinase homologues, TvaC$_{S-87}$ (active kinase) and TvaE$_{S-87}$ (inactive kinase), in the TVA pathway, suggesting that, while KinD is likely to be an active kinase, KinH is an inactive kinase homologue.

The conserved catalytic residue Asp is indicated by red square. Mg^{2+}-chelating residues Asn/Asp are highlighted with blue circle.

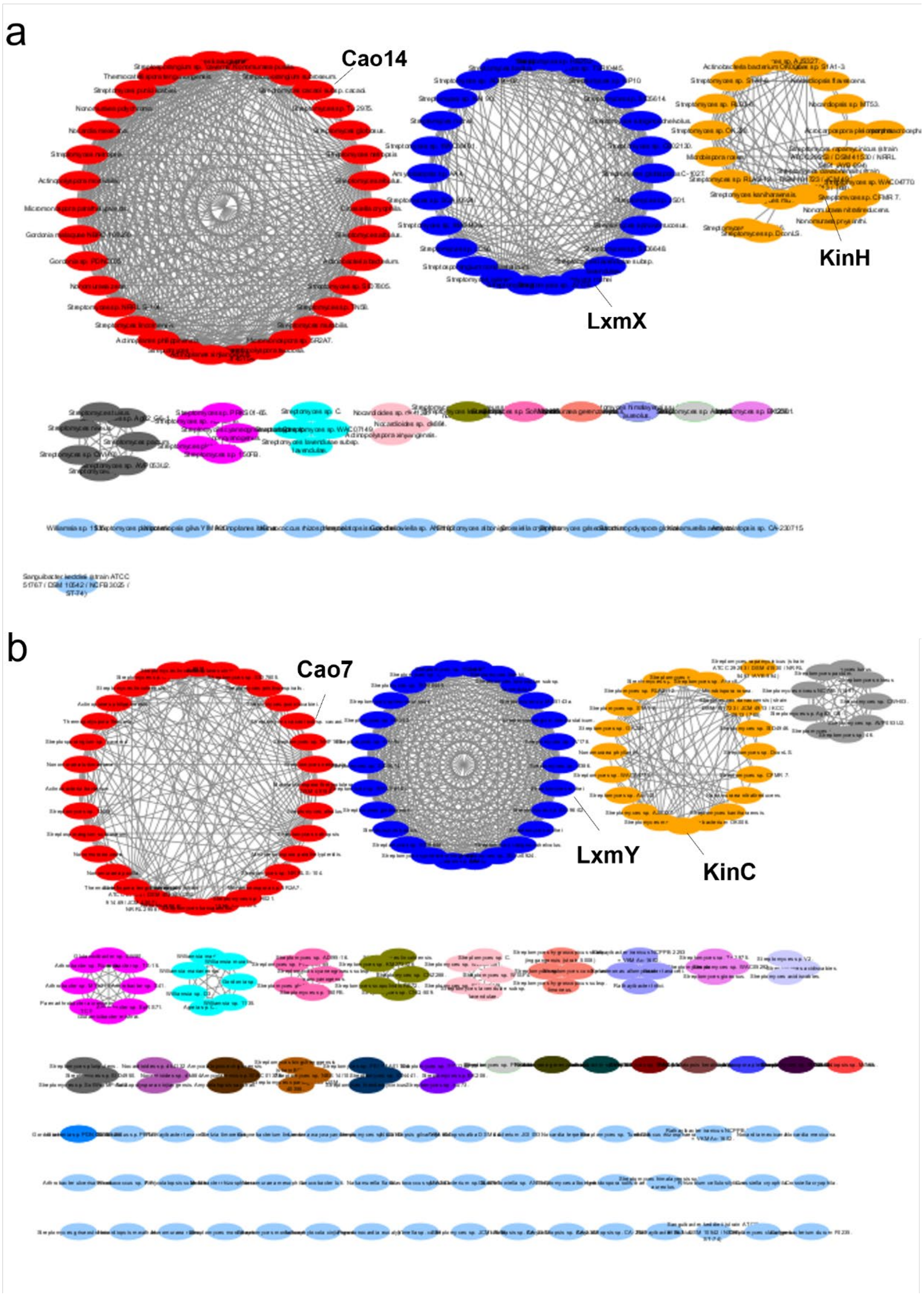

Supplementary Fig 43. Sequence similarity network (SSN) using the tools of the Enzyme Function Initiative (EFI).

a. SSN analysis suggested KinC forms a separate cluster with the putative lyases in the pathways of other RiPPs such as LxmY in lexapeptide and Cao7 in cacaoidin. **b**. SSN analysis suggested KinH forms a separate cluster with the putative inactive kinase homologues in the pathways of other RiPPs such as LxmX in lexapeptide and Cao14 in cacaoidin.

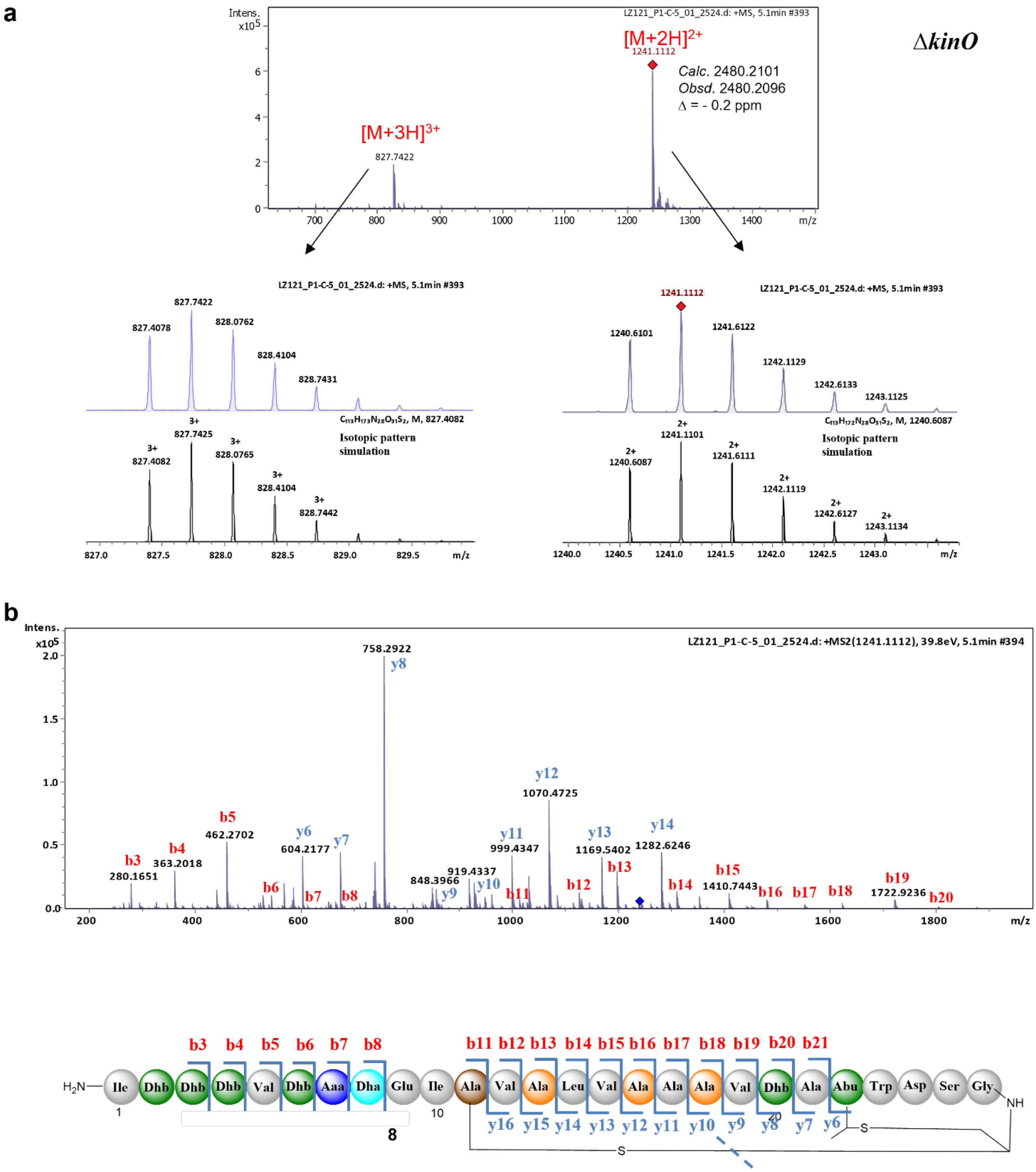

Supplementary Fig. 44. MS analysis of the intermediate **8** accumulated in the *kin*O variant. **a**. HR-MS analysis of **9**. **b**. MS^2 fragmentation analysis of the corresponding ion.

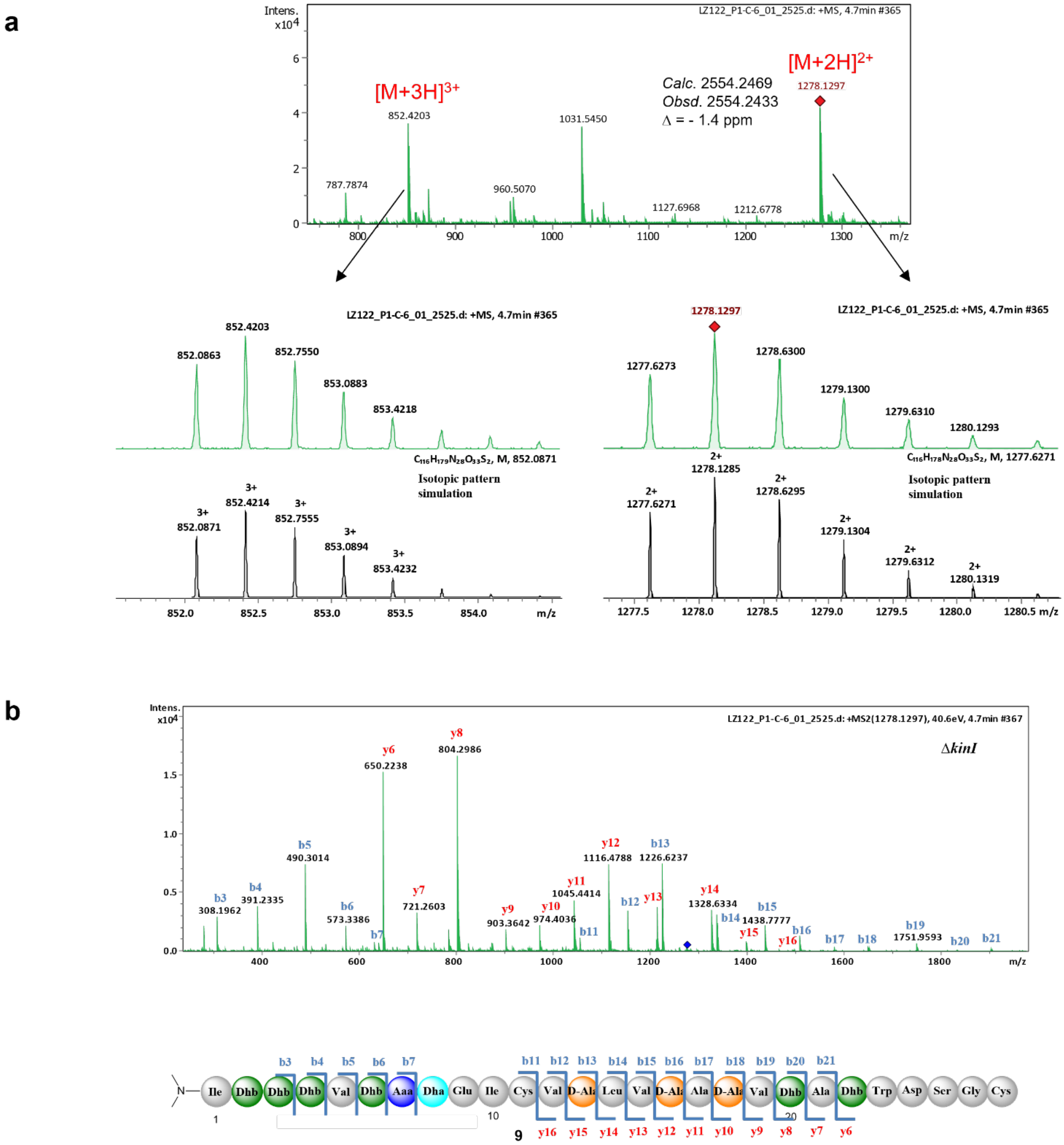

Supplementary Fig. 45. MS analysis of the intermediate **9** accumulated in the *kinI* variant. **a**. HR-MS analysis of **9**. **b**. MS^2 fragmentation analysis of the corresponding ion.

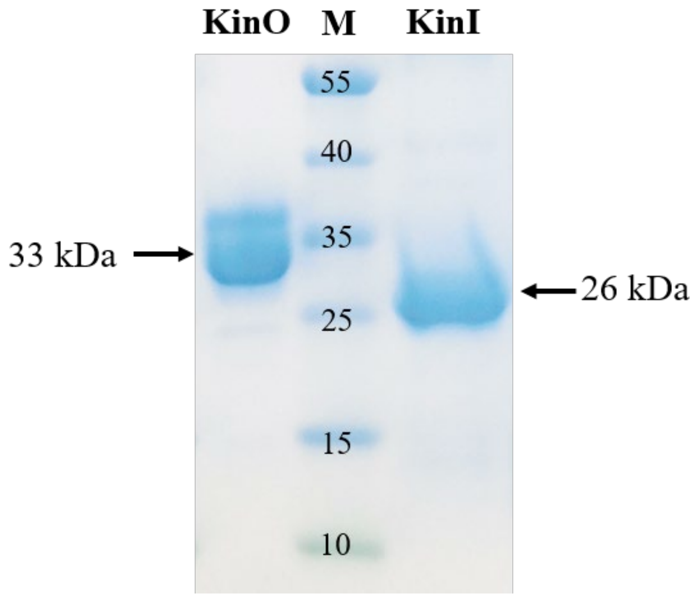

Supplementary Fig. 46. SDS page analysis of recombinant KinI (Right) and KinO (Left) with the corrected molecular weights, respectively.

All experiments were repeated independently with similar results for three times.

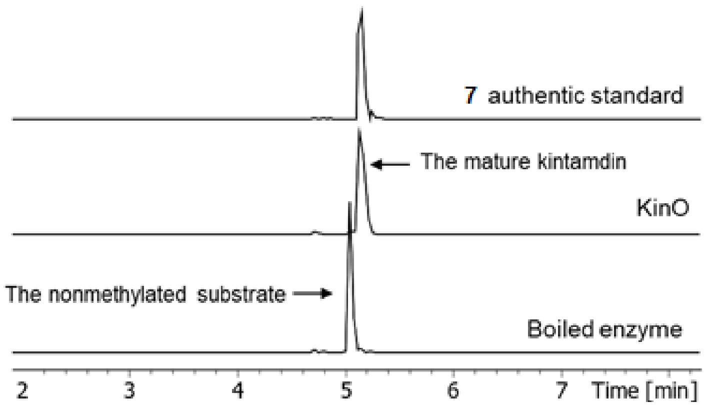

Supplementary Fig. 47. EIC analysis of the product generated in the assay of incubation of KinO with **7** accumulated.

a

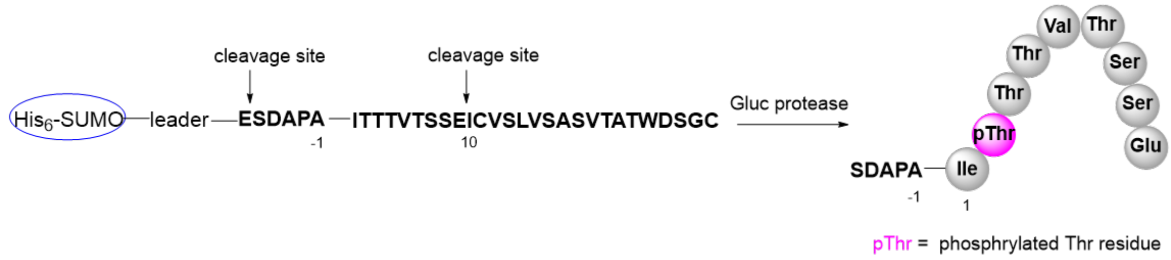

b

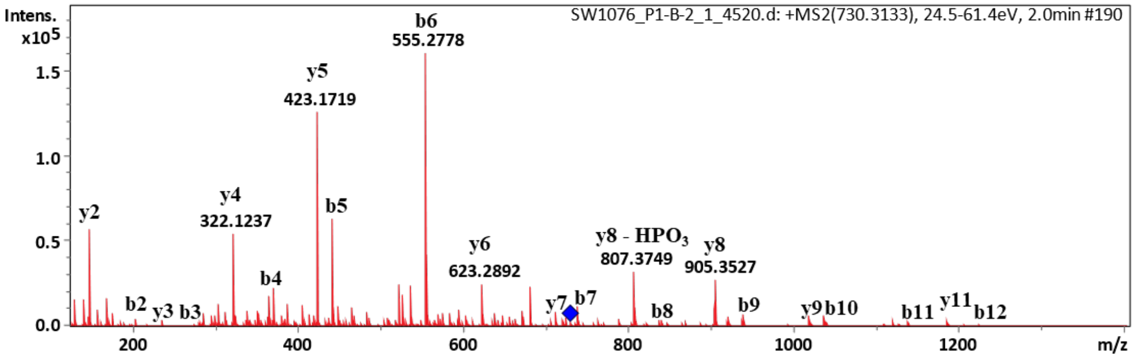

c

		b-ions				y-ions			
#	Residue		Calculated	Observed	Mass error (ppm)		Calculated	Observed	Mass error (ppm)
1	Ser	-	-	-	-	-	-	-	-
2	Asp	b2	203.0662	203.0674	-5.6	y12	1257.5610	1257.5621	-0.9
3	Ala	b3	274.1034	274.1029	1.6	y11	1186.5239	1186.5275	-3.0
4	Phe	b4	371.1561	371.1553	2.3	y10	-	-	-
5	Ala	b5	442.1932	442.1929	0.8	y9	1018.4340	1018.4375	-3.4
6	Ile	b6	555.2773	555.2778	-0.9	y8	905.3499	905.3527	-3.1
7	pThr	b7	736.2913	736.2928	-2.0	y7	724.3359	724.3377	-2.5
8	Thr	b8	837.3390	837.3412	-2.6	y6	623.2883	623.2892	-1.6
9	Thr	b9	938.3867	938.3904	-3.9	y5	522.2406	522.2410	-0.8
10	Val	b10	1037.4551	1037.4583	-3.1	y4	423.1722	423.1719	0.7
11	Thr	b11	1138.5028	1138.5045	-1.5	y3	322.1245	322.1237	2.6
12	Ser	b12	1225.5430	1225.5403	2.2	y2	235.0925	235.0935	-4.4
13	Ser	b13	1312.5668	1312.5658	0.8	y1	148.0604	148.0609	-3.0
14	Glu	-	-	-	-	-	-	-	-

Supplementary Fig. 48. MS^2 fragmentation analysis of the first peptidyl fragments from the Gluc-treated linear peptide from the co-expression of *kin*AD in *E. coli*.

a. Schematic process of protease treatment of purified SUMO-His_6-KinA(D), leading the identification of the monophosphorylated CP fragment (Ser-(-5) to Glu-9). **b**. MS^2 fragmentation spectrum of this peptidyl fragments. **c**. The table of y- and b- ions deduced from MS^2 fragmentation spectrum.

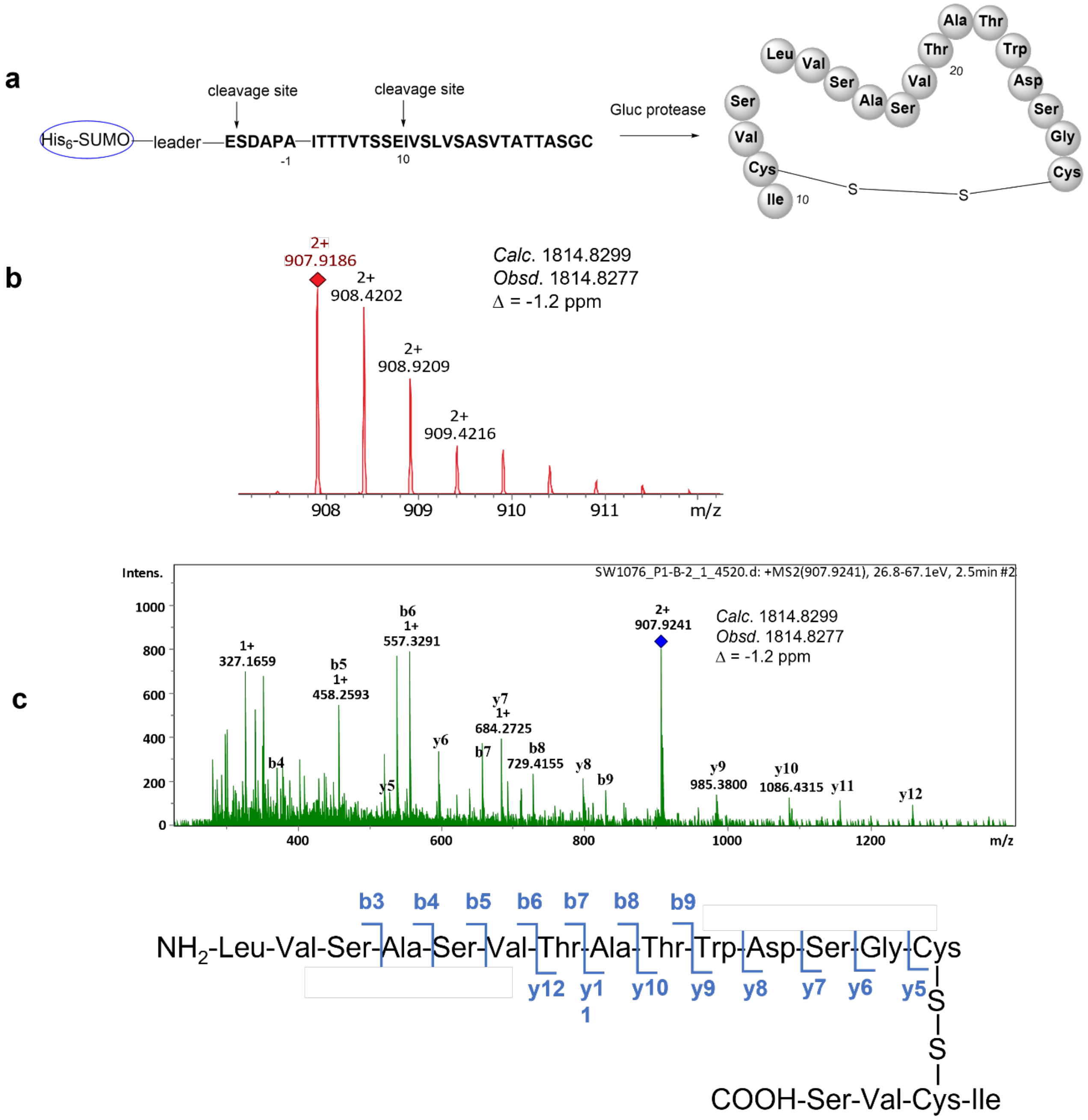

d

b-ions				y-ions			
	Calculated	Observed	Mass error (ppm)		Calculated	Observed	Mass error (ppm)
b3	300.1918	300.1906	4.1	y12	1258.5078	1258.5114	-2.8
b4	371.2289	371.2302	-3.6	y11	1157.4602	1157.4700	-8.5
b5	458.2609	458.2593	3.5	y10	1086.4231	1086.4315	-7.8
b6	557.3293	557.3291	0.3	y9	985.3754	985.3800	-4.7
b7	658.3770	658.3773	-0.4	y8	799.2961	799.2981	-2.5
b8	729.4141	729.4155	-1.9	y7	684.2691	684.2725	-4.9
b9	830.4618	830.4615	0.4	y6	597.2371	597.2367	-0.7
				y5	540.2156	540.2193	-6.8

Supplementary Fig. 49. MS and MS^2 fragmentation analysis of the unmodified peptidyl fragment from the co-expression of *kin*AD in *E. coli* after Gluc-treatment.

a. Schematic process of protease treatment of purified SUMO-His$_6$-KinA(D), leading the identification of the unmodified CP fragment. **b**. MS analysis of the unmodified CP fragment (Ile-10 to Cys-27). **c**. MS^2 fragmentation spectrum of this peptidyl fragments. **d**. The predicted y- and b-ions with the table of y- and b- ions deduced from MS^2 fragmentation spectrum.

a

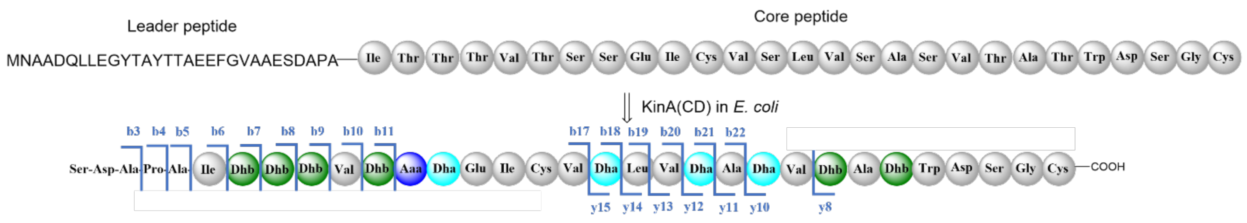

b

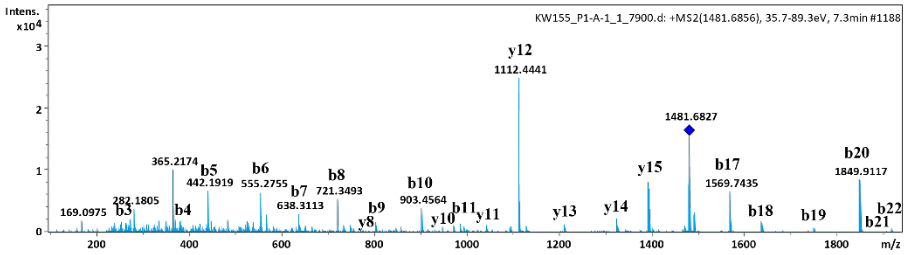

c

b-ions	Calculated	Observed	Mass error (ppm)	y-ions	Calculated	Observed	Mass error (ppm)
b3	274.1034	274.1036	-1.0	y15	1393.6205	1393.6181	1.7
b4	371.1561	371.1555	1.8	y14	1324.5990	1324.5977	1.0
b5	442.1932	442.1919	3.0	y13	1211.5150	1211.5157	-0.6
b6	555.2773	555.2755	3.2	y12	1112.4466	1112.4441	2.3
b7	638.3144	638.3113	4.8	y11	1043.4251	1043.4216	3.4
b8	721.3515	721.3493	3.1	y10	972.3880	972.3857	2.3
b9	804.3886	804.3882	0.6	y8	804.2981	804.2932	6.2
b10	903.4571	903.4564	0.7				
b11	986.4942	986.4911	3.1				
b17	1568.7414	1568.7399	0.9				
b18	1637.7628	1637.7633	-0.3				
b19	1750.8469	1750.8396	4.2				
b20	1849.9153	1849.9117	2.0				
b21	1918.9368	1918.9454	-4.5				
b22	1989.9739	1989.9669	3.5				

Supplementary Fig. 50. MS^2 fragmentation analysis of the first peptidyl fragments from the Gluc-treated linear peptide from the co-expression of *kin*ACD in *E. coli*.

a. Schematic process of protease treatment of purified SUMO-His$_6$-KinA(CD), leading the identification of the fully dehydrated CP. **b**. MS^2 fragmentation spectrum of this peptide. **c**. The table of y- and b- ions deduced from MS^2 fragmentation spectrum.

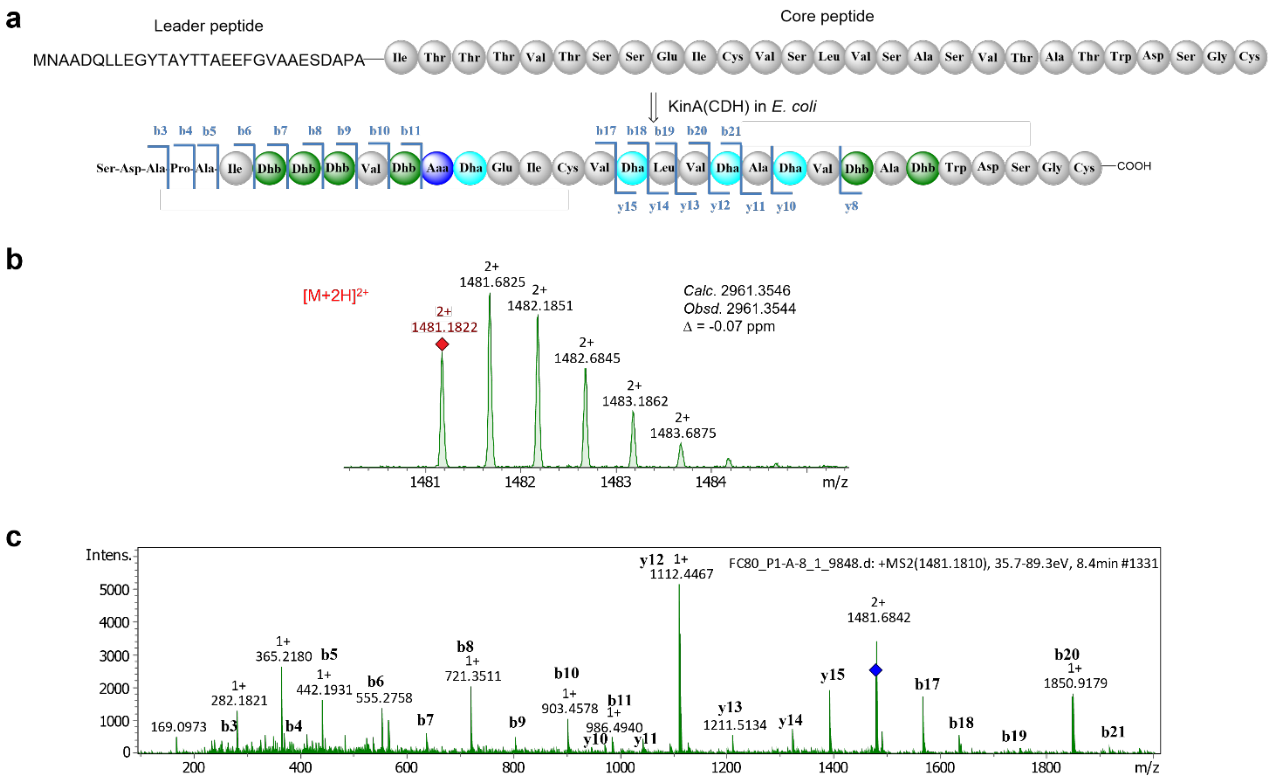

d

	b-ions				y-ions		
	Calculated	Observed	Mass error (ppm)		Calculated	Observed	Mass error (ppm)
b3	274.1034	274.1031	1.0	y15	1393.6205	1393.6187	1.3
b4	371.1561	371.1558	1.0	y14	1324.5990	1324.5977	1.0
b5	442.1932	442.1931	0.4	y13	1211.5150	1211.5134	1.3
b6	555.2773	555.2758	2.7	y12	1112.4466	1112.4467	-0.2
b7	638.3144	638.3127	2.7	y11	1043.4251	1043.4298	-4.5
b8	721.3515	721.3511	0.6	y10	972.3880	972.3878	0.2
b9	804.3886	804.3872	1.8	y8	804.2981	804.3000	-2.3
b10	903.4571	903.4578	-0.8				
b11	986.4942	986.4940	0.2				
b17	1568.7414	1568.7406	0.5				
b18	1637.7628	1637.7702	-4.5				
b19	1750.8469	1750.8517	-2.8				
b20	1849.9153	1849.9182	-1.6				
b21	1918.9368	1918.9329	2.0				

Supplementary Fig. 51. MS and MS^2 fragmentation analysis of the modified CP from the co-expression of *kin*ACDH in *E. coli* after Gluc-treatment.

a. Schematic process of protease treatment of purified SUMO-His$_6$-KinA(CDH), leading the identification of the dehydrated CP **11**. **b**. MS analysis of the dehydrated CP (Ser-(-5) to Cys-27). **c**. MS^2 fragmentation spectrum of this peptidyl fragment. **d**. The predicted y- and b- ions with the table of y- and b- ions deduced from MS^2 fragmentation spectrum.

a

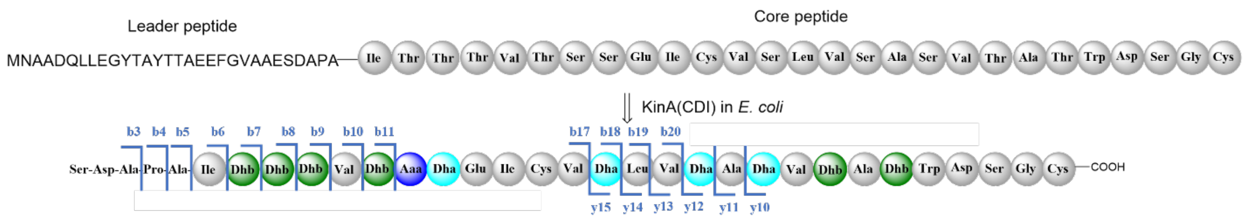

b

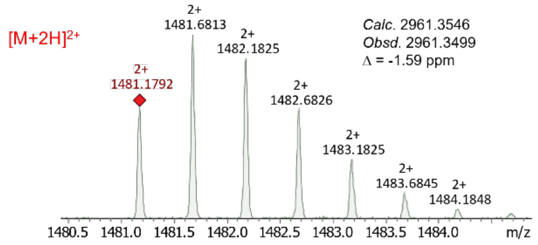

c

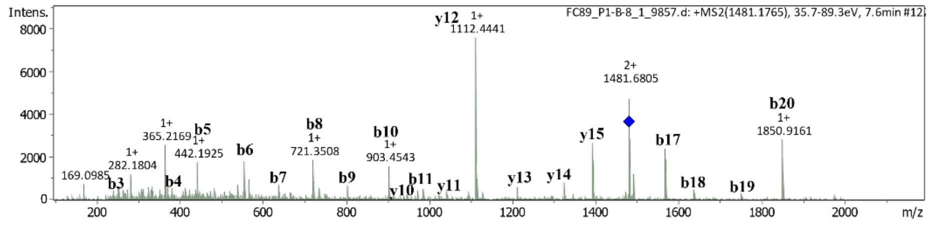

d

b-ions				y-ions			
	Calculated	Observed	Mass error (ppm)		Calculated	Observed	Mass error (ppm)
b3	274.1034	274.1023	3.9	y15	1393.6205	1393.6183	1.6
b4	371.1561	371.1544	4.8	y14	1324.5990	1324.6018	-2.1
b5	442.1932	442.1925	1.6	y13	1211.5150	1211.5140	0.8
b6	555.2773	555.2762	2.0	y12	1112.4466	1112.4441	2.2
b7	638.3144	638.3133	1.8	y11	1043.4251	1043.4235	1.5
b8	721.3515	721.3508	1.0	y10	972.3880	972.3860	2.0
b9	804.3886	804.3830	7.1				
b10	903.4571	903.4543	3.1				
b11	986.4942	986.4925	1.7				
b17	1568.7414	1568.7406	-0.1				
b18	1637.7628	1637.7539	5.4				
b19	1750.8469	1750.8443	1.5				
b20	1849.9153	1849.9153	0.0				

Supplementary Fig. 52. MS and MS^2 fragmentation analysis of the modified CP from the co-expression of *kin*ACDI in *E. coli* after Gluc-treatment.

a. Schematic process of protease treatment of purified SUMO-His$_6$-KinA(CDI), leading the identification of the dehydrated CP **11**. **b**. MS analysis of the dehydrated CP (Ser-(-5) to Cys-27). **c**. MS^2 fragmentation spectrum of this peptidyl fragment. **d**. The predicted y- and b- ions with the table of y- and b- ions deduced from MS^2 fragmentation spectrum.

a

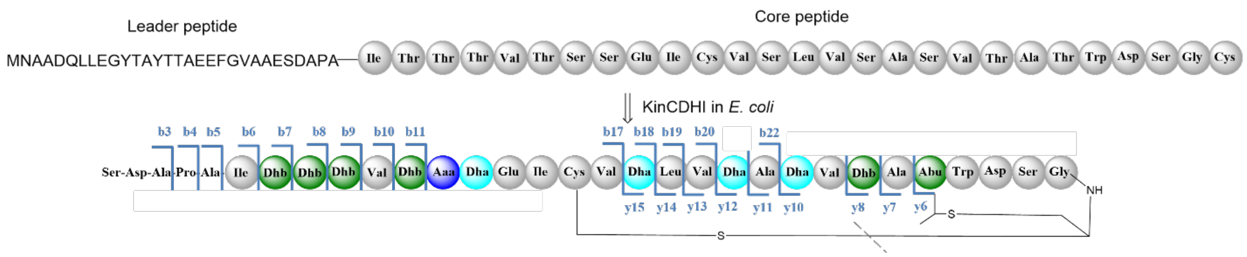

b

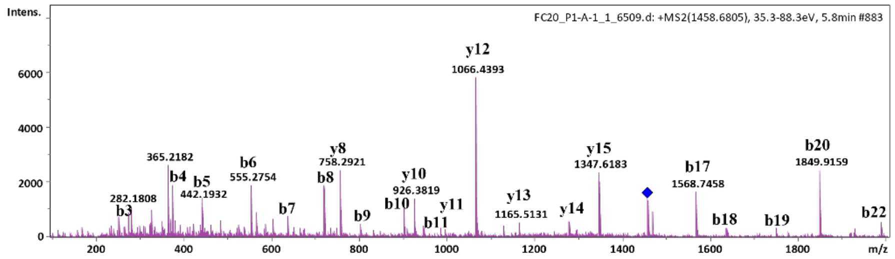

c

	b-ions				y-ions		
	Calculated	Observed	Mass error (ppm)		Calculated	Observed	Mass error (ppm)
b3	274.1034	274.1046	-4.6	y15	1347.6150	1347.6183	-2.4
b4	371.1561	371.1561	6.2	y14	1278.5936	1278.5951	-1.2
b5	442.1932	442.1932	0.0	y13	1165.5095	1165.5131	-3.1
b6	555.2773	555.2754	3.3	y12	1066.4411	1066.4393	1.7
b7	638.3144	638.3164	-3.0	y11	997.4220	997.4196	-2.4
b8	721.3515	721.3522	-0.9	y10	926.3825	926.3819	0.6
b9	804.3886	804.3905	-2.3	y9	-	-	-
b10	903.4571	903.4557	1.5	y8	758.2921	758.2926	0.7
b11	986.4942	986.4955	-1.4	y7	675.2524	675.2555	4.7
b17	1568.7414	1568.7458	-2.8	y6	604.2184	604.2158	4.3
b18	1637.7628	1637.7744	-7.1				
b19	1750.8469	1750.8410	3.4				
b20	1849.9153	1849.9159	-0.3				
b22	1989.9739	1989.9674	3.2				

Supplementary Fig. 53. MS^2 fragmentation analysis of the modified CP from the co-expression of *kin*ACDHI in *E. coli* after Gluc-treatment.

a. Schematic process of protease treatment of purified SUMO-His$_6$-KinA(CDHI), leading the identification of the dehydrated cyclic peptide **12**. **b**. MS^2 fragmentation spectrum of this dehydrated cyclic peptide. **c**. The predicted y- and b- ions with the table of y- and b- ions deduced from MS^2 fragmentation spectrum.

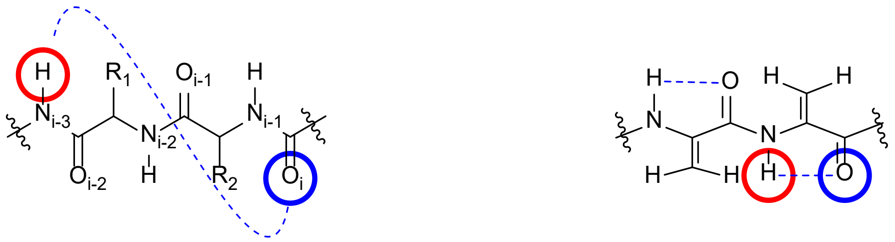

Dash lines represent intra-molecular H-bonding

Supplementary Fig. 54. Peptides containing dehydroamino acid residues tend to be 2.0_5-helix (left) [15, 16] where some peptides containing normal α-amino acid form 3_{10}-helix (right).

3_{10}-helix constitutes nearly 10-15% of all helices in protein secondary structures. The 2.0_5-helix is 2 residue per turn and stabilized by H-bonds encompassing 5-membered pseudo-cycles between NH and the carbonyl of the same amino acid residue as shown above. As such, dehydroamino acid stretches arrange as flat peptides. The N-to-C termini distance of such foldamers is the longest an α-peptide can span, almost double than that of an α-helix containing normal amino acid residues.

a

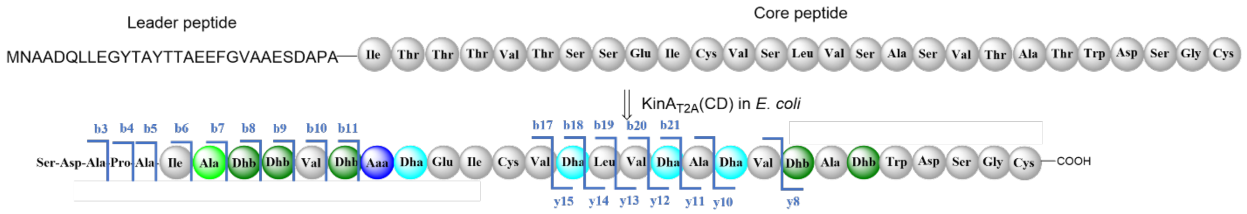

b

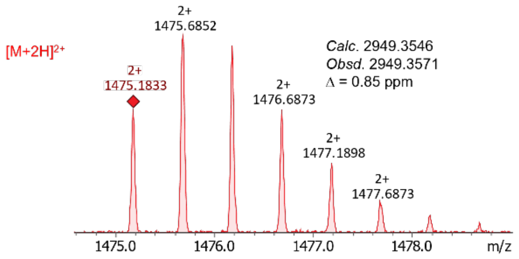

c

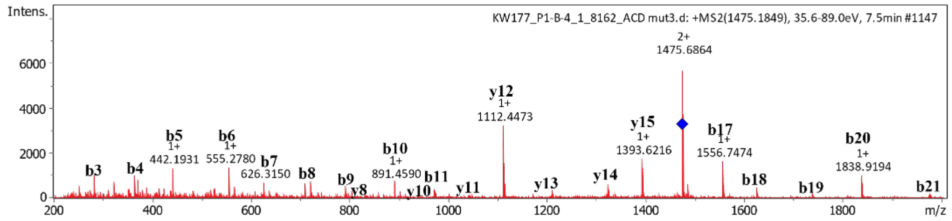

d

	b-ions				y-ions		
	Calculated	Observed	Mass error (ppm)		Calculated	Observed	Mass error (ppm)
b3	274.1034	274.1057	-8.5	y15	1393.6205	1393.6216	-0.8
b4	371.1561	371.1569	-1.1	y14	1324.5990	1324.5997	-0.5
b5	442.1932	442.1931	0.3	y13	1211.5150	1211.5195	-3.7
b6	555.2773	555.2780	-1.3	y12	1112.4466	1112.4473	-0.6
b7	626.3144	626.3150	-0.9	y11	1043.4251	1043.4318	-6.4
b8	709.3515	709.3524	-1.2	y10	972.3880	972.3889	-0.9
b9	792.3886	792.3900	-1.8	y8	804.2981	804.3020	-4.8
b10	891.4571	891.4590	-2.2				
b11	974.4942	974.4926	1.6				
b17	1556.7414	1556.7474	-3.9				
b18	1625.7628	1625.7619	0.6				
b19	1738.8469	1738.8425	2.5				
b20	1837.9153	1837.9242	-4.8				
b21	1906.9368	1906.9295	3.8				

Supplementary Fig. 55. MS and MS^2 fragmentation analysis of the modified CP from the co-expression of *kin*A$_{T2A}$CD in *E. coli* after Gluc-treatment.

a. Schematic process of protease treatment of purified SUMO-His$_6$-KinA$_{T2A}$(CD), leading the identification of the fully dehydrated CP of KinA$_{T2A}$. **b**. MS analysis of the dehydrated CP of KinA$_{T2A}$. **c**. MS^2 fragmentation spectrum of this peptidyl fragment. **d**. The predicted y- and b- ions with the table of y- and b- ions deduced from MS^2 fragmentation spectrum.

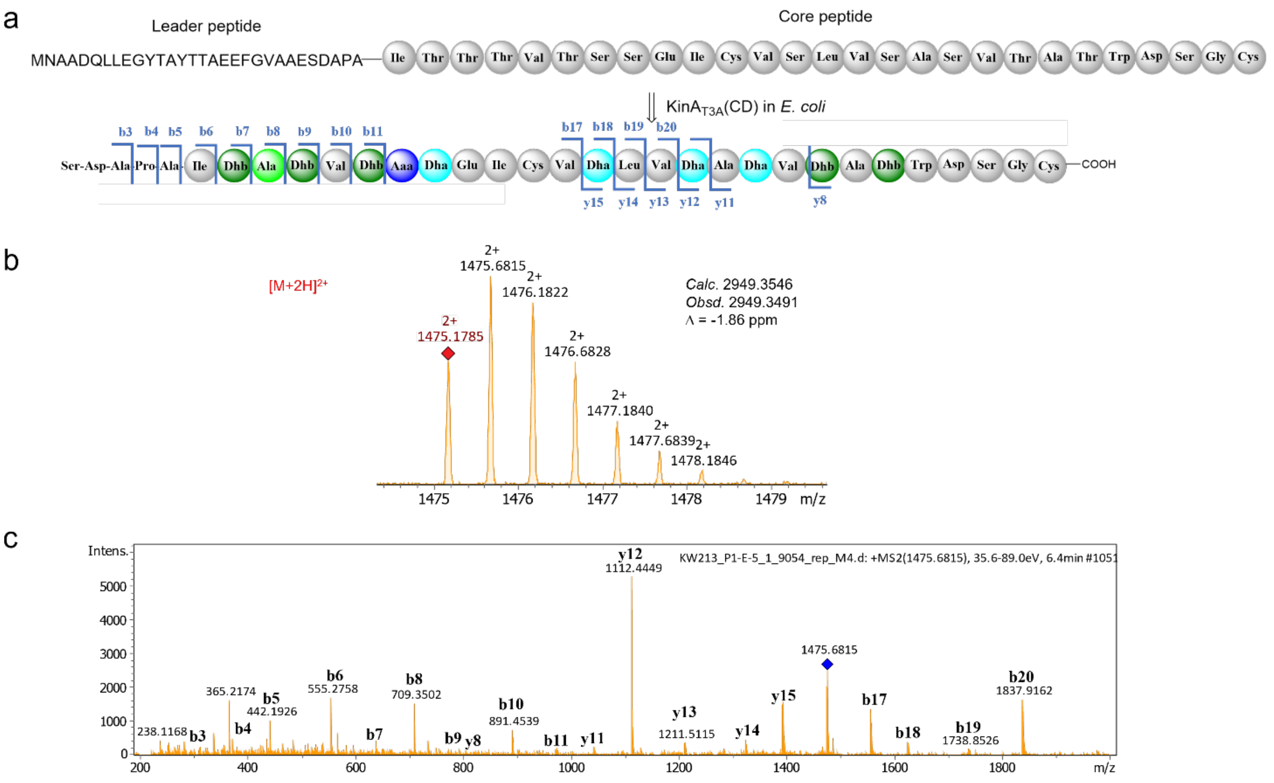

d

b-ions				y-ions			
	Calculated	Observed	Mass error (ppm)		Calculated	Observed	Mass error (ppm)
b3	274.1034	274.1010	8.7	y15	1393.6205	1393.6196	0.7
b4	371.1561	371.1536	6.8	y14	1324.5990	1324.5970	1.6
b5	442.1932	442.1926	1.4	y13	1211.5150	1211.5115	2.9
b6	555.2773	555.2758	2.8	y12	1112.4466	1112.4449	1.5
b7	638.3144	638.3139	0.8	y11	1043.4251	1043.4303	-5.0
b8	709.3515	709.3502	1.9	y8	804.2981	804.2973	1.1
b9	792.3886	792.3923	-4.7				
b10	891.4571	891.4539	3.6				
b11	974.4942	974.4859	8.5				
b17	1556.7414	1556.7416	-1.0				
b18	1625.7628	1625.7617	0.7				
b19	1738.8469	1738.8526	-3.3				
b20	1837.9153	1837.9162	-0.5				

Supplementary Fig. 56. MS and MS^2 fragmentation analysis of the modified CP from the co-expression of *kin*A$_{T3A}$CD in *E. coli* after Gluc-treatment.

a. Schematic process of protease treatment of purified SUMO-His$_6$-KinA$_{T3A}$(CD), leading the identification of the dehydrated CP of KinA$_{T3A}$. **b**. MS analysis of the dehydrated CP of KinA$_{T3A}$. **c**. MS^2 fragmentation spectrum of this peptidyl fragment. **d**. The predicted y- and b- ions with the table of y- and b- ions deduced from MS^2 fragmentation spectrum.

a

b

c

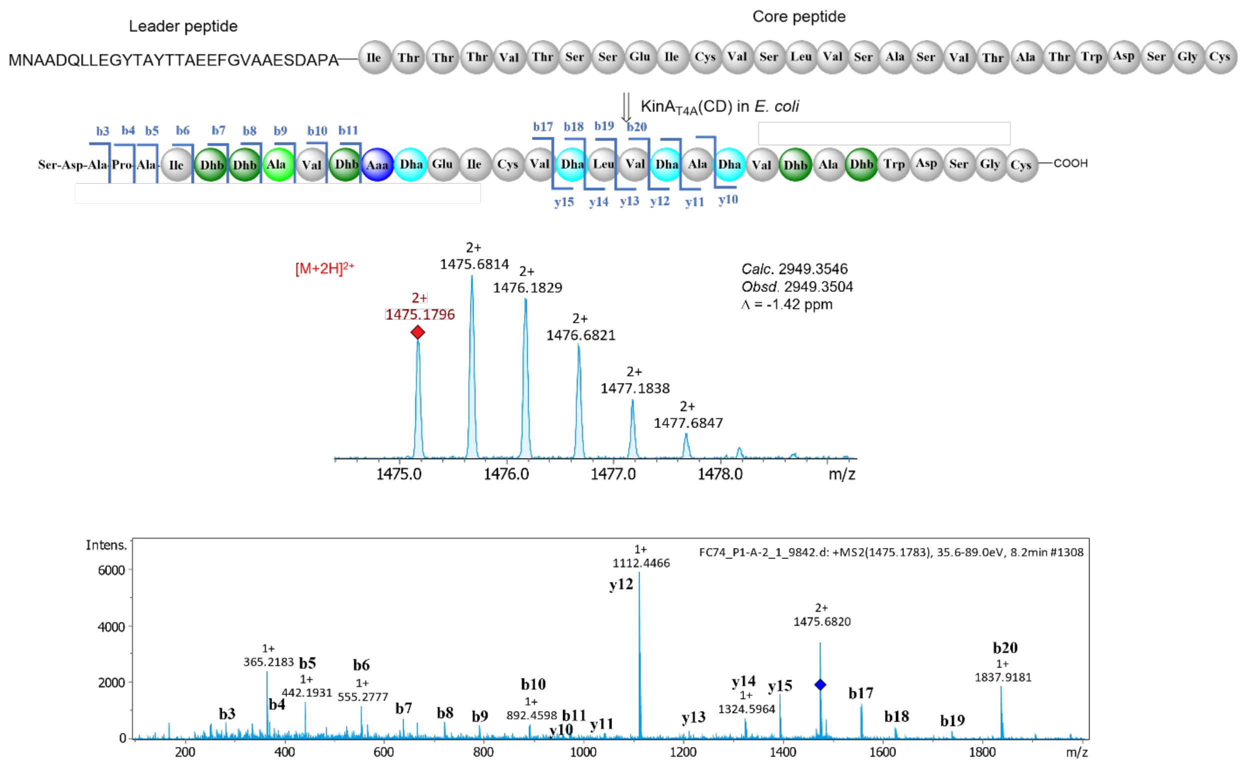

d

b-ions				y-ions			
	Calculated	Observed	Mass error (ppm)		Calculated	Observed	Mass error (ppm)
b3	274.1034	274.1030	1.3	y15	1393.6205	1393.6190	1.1
b4	371.1561	371.1533	7.5	y14	1324.5990	1324.5964	2.0
b5	442.1932	442.1931	0.4	y13	1211.5150	1211.5127	1.9
b6	555.2773	555.2777	-0.6	y12	1112.4466	1112.4466	0.0
b7	638.3144	638.3138	0.9	y11	1043.4251	1043.4270	-1.8
b8	721.3515	721.3486	4.1	y10	972.3880	972.3815	6.6
b9	792.3886	792.3897	-1.3				
b10	891.4571	891.4566	0.5				
b11	974.4942	974.4980	-3.9				
b17	1556.7414	1556.7427	-0.9				
b18	1625.7628	1625.7663	-2.1				
b19	1738.8469	1738.8405	3.6				
b20	1837.9153	1837.9189	-1.5				

Supplementary Fig. 57. MS and MS^2 fragmentation analysis of the modified CP from the co-expression of *kin*A$_{T4A}$CD in *E. coli* after Gluc-treatment.

a. Schematic process of protease treatment of purified SUMO-His$_6$-KinA$_{T4A}$(CD), leading the identification of the dehydrated CP of KinA$_{T4A}$. **b**. MS analysis of the dehydrated CP of KinA$_{T4A}$. **c**. MS^2 fragmentation spectrum of this peptidyl fragment. **d**. The predicted y- and b- ions with the table of y- and b- ions deduced from MS^2 fragmentation spectrum.

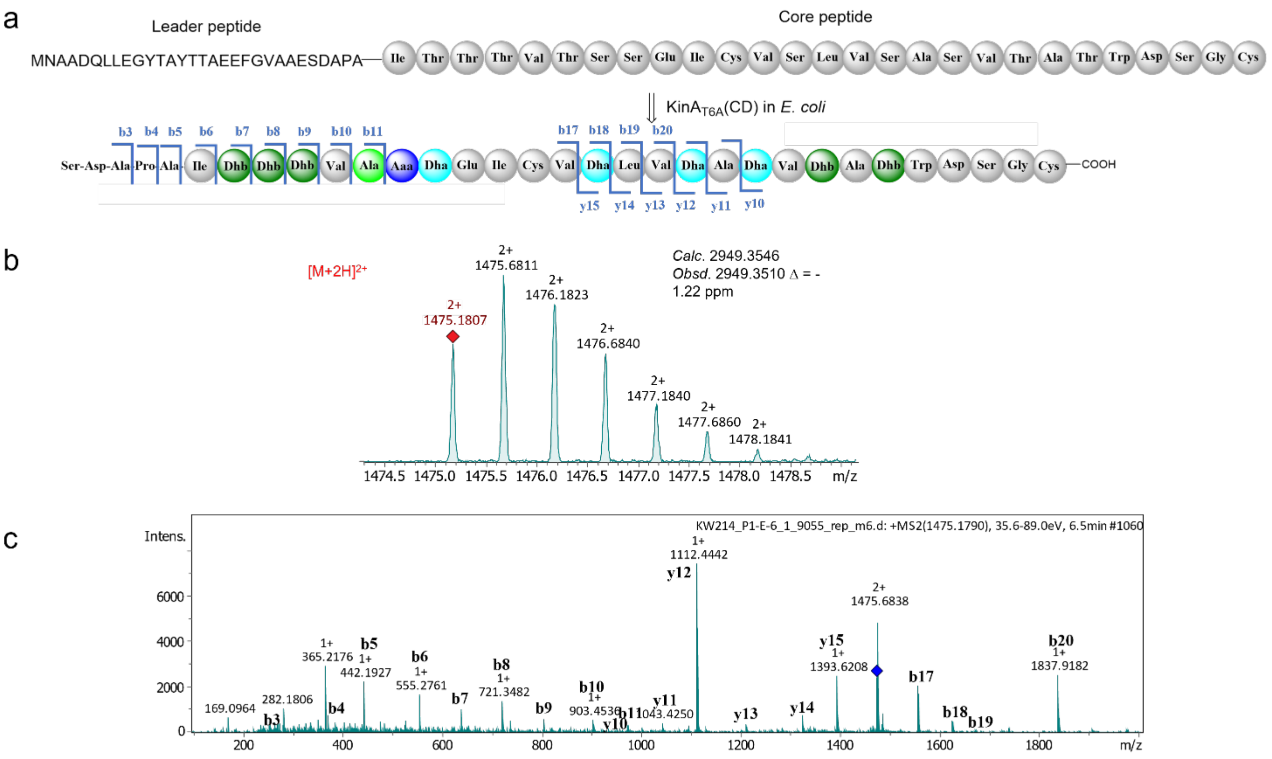

d

b-ions	Calculated	Observed	Mass error (ppm)	y-ions	Calculated	Observed	Mass error (ppm)
b3	274.1034	274.1030	1.5	y15	1393.6205	1393.6208	-0.2
b4	371.1561	371.1549	3.4	y14	1324.5990	1324.5942	3.6
b5	442.1932	442.1927	1.2	y13	1211.5150	1211.5051	8.1
b6	555.2773	555.2761	2.2	y12	1112.4466	1112.4442	2.1
b7	638.3144	638.3125	3.0	y11	1043.4251	1043.4250	0.1
b8	721.3515	721.3482	4.6	y10	972.3880	972.3834	4.8
b9	804.3886	804.3896	-1.2				
b10	903.4571	903.4536	3.8				
b11	974.4942	974.4944	-0.2				
b17	1556.7414	1556.7397	1.1				
b18	1625.7628	1625.7576	3.2				
b19	1738.8469	1738.8495	-1.5				
b20	1837.9153	1837.9182	-1.6				

Supplementary Fig. 58. MS and MS^2 fragmentation analysis of the modified CP from the co-expression of *kin*A$_{T6A}$CD in *E. coli* after Gluc-treatment.

a. Schematic process of protease treatment of purified SUMO-His$_6$-KinA$_{T6A}$(CD), leading the identification of the dehydrated CP of KinA$_{T6A}$. **b**. MS analysis of the dehydrated CP of KinA$_{T6A}$. **c**. MS^2 fragmentation spectrum of this peptidyl fragment. **d**. The predicted y- and b- ions with the table of y- and b- ions deduced from MS^2 fragmentation spectrum.

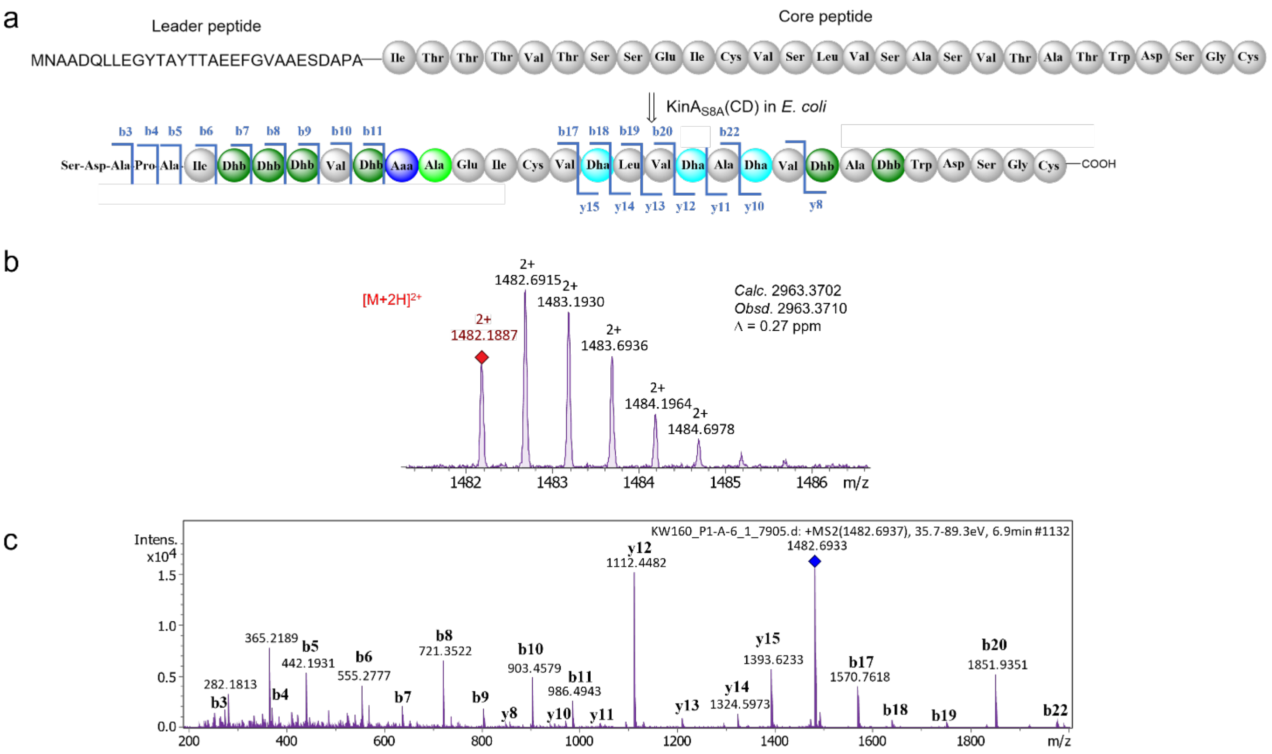

d

	b-ions				y-ions		
	Calculated	Observed	Mass error (ppm)		Calculated	Observed	Mass error (ppm)
b3	274.1034	274.1027	2.4	y15	1393.6205	1393.6233	-2.0
b4	371.1561	371.1569	-2.1	y14	1324.5990	1324.5973	1.3
b5	442.1932	442.1931	0.4	y13	1211.5150	1211.5168	-1.5
b6	555.2773	555.2777	-0.7	y12	1112.4466	1112.4482	-1.4
b7	638.3144	638.3147	-0.5	y11	1043.4251	1043.4249	0.2
b8	721.3515	721.3522	-0.9	y10	972.3880	972.3932	-4.5
b9	804.3886	804.3884	0.4	y8	804.2981	804.2977	0.5
b10	903.4571	903.4579	-0.9				
b11	986.4942	986.4943	-0.1				
b17	1570.7570	1570.7618	-3.1				
b18	1639.7785	1639.7807	-1.4				
b19	1752.8625	1752.8635	-0.5				
b20	1851.9309	1851.9351	-2.2				
b22	1991.9895	1991.9906	-0.5				

Supplementary Fig. 59. MS and MS^2 fragmentation analysis of the modified CP from the co-expression of *kin*A_{S8A}CD in *E. coli* after Gluc-treatment.

a. Schematic process of protease treatment of purified SUMO-His_6-KinA_{S8A}(CD), leading the identification of the dehydrated CP of KinA_{S8A}. **b**. MS analysis of the dehydrated CP of KinA_{S8A}. **c**. MS^2 fragmentation spectrum of this peptidyl fragment. **d**. The predicted y- and b- ions with the table of y- and b- ions deduced from MS^2 fragmentation spectrum.

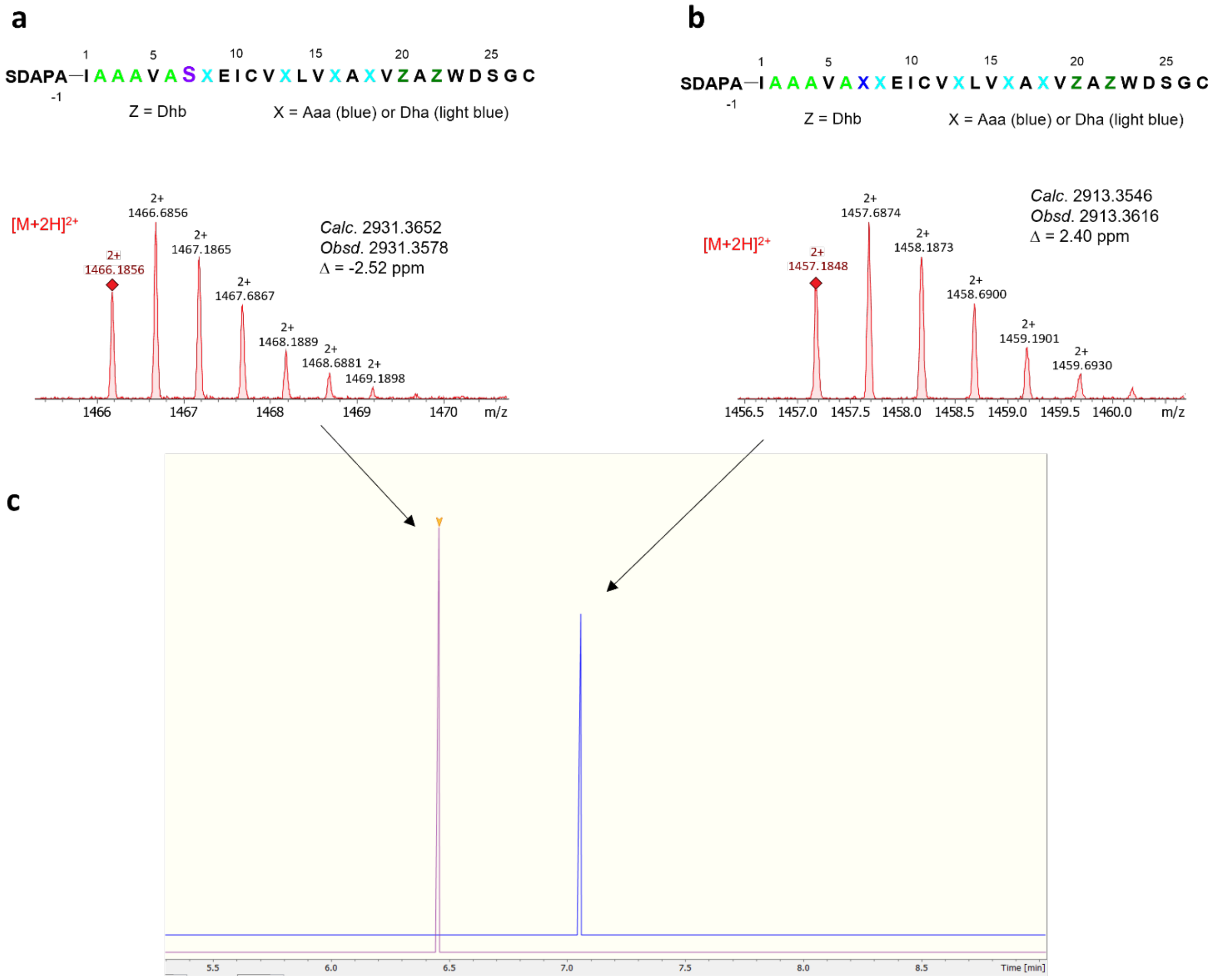

Supplementary Fig. 60. MS comparison of ion intensities of the fully dehydrated and partially dehydrated linear peptides from the co-expression of *kin*A_{TtoA}CD in *E. coli* after Gluc-treatment.

a. MS spectrum of the partially dehydrated linear peptide. **b**. MS spectrum of the fully dehydrated linear peptide. **c**. MS comparison of extracted ion chromatographs of the partially and fully dehydrated peptides.

a

b

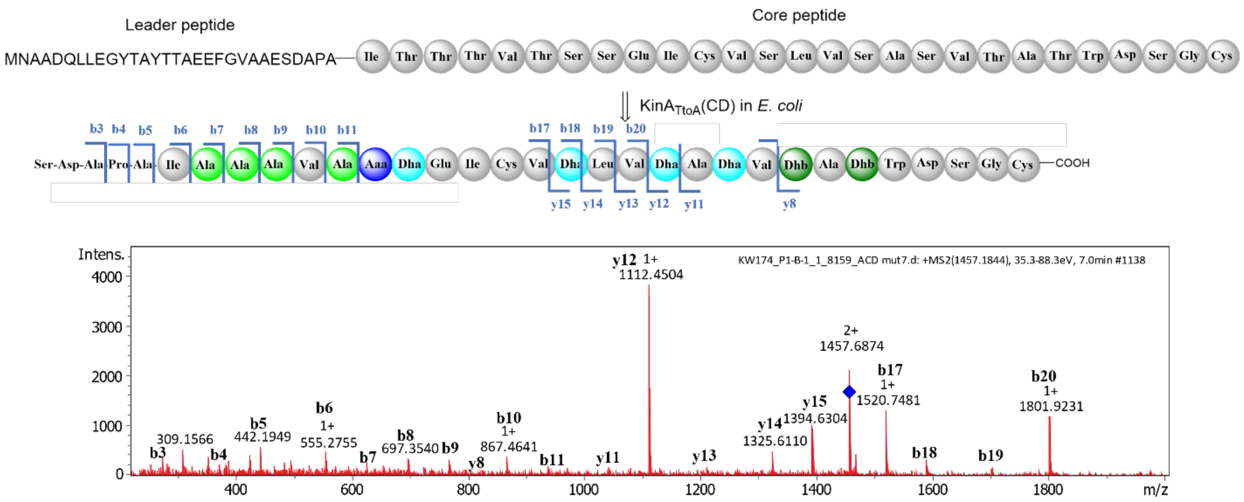

c

	b-ions				y-ions		
	Calculated	Observed	Mass error (ppm)		Calculated	Observed	Mass error (ppm)
b3	274.1034	274.1048	-5.3	y15	1393.6205	1393.6304	-2.0
b4	371.1561	371.1579	-4.8	y14	1324.5990	1324.5954	-3.9
b5	442.1932	442.1941	-3.7	y13	1211.5150	1211.5166	-1.3
b6	555.2773	555.2755	3.3	y12	1112.4466	1112.4504	-3.4
b7	626.3144	626.3145	-0.1	y11	1043.4251	1043.4331	-7.6
b8	697.3515	697.3540	-3.5	y8	804.2981	804.3019	-4.7
b9	768.3886	768.3876	1.3				
b10	867.4571	867.4641	-8.2				
b11	938.4942	938.4943	-0.1				
b17	1520.7414	1520.7481	-4.5				
b18	1589.7628	1589.7674	-2.9				
b19	1702.8469	1702.8535	-3.9				
b20	1801.9153	1801.9231	-4.3				

Supplementary Fig. 61. MS and MS^2 fragmentation analysis of the fully dehydrated CP from the co-expression of *kin*A_{TtoA}CD in *E. coli* after Gluc-treatment.

a. Schematic process of protease treatment of purified SUMO-His_6-KinA_{TtoA}(CD), leading the identification of the fully dehydrated CP of KinA_{TtoA}. **b**. MS^2 fragmentation spectrum of this peptidyl fragment. **c**. The predicted y- and b- ions with the table of y- and b- ions deduced from MS^2 fragmentation spectrum.

a

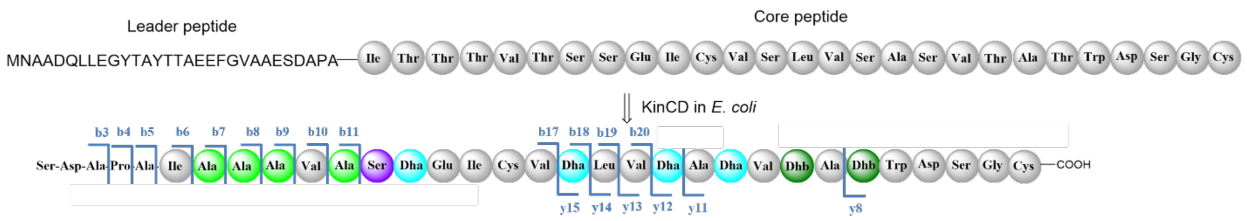

b

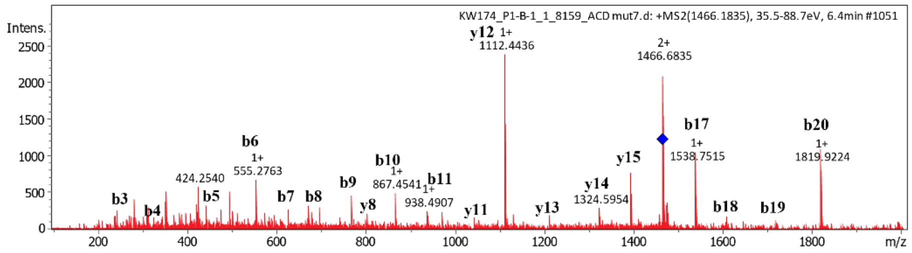

c

b-ions				y-ions			
	Calculated	Observed	Mass error (ppm)		Calculated	Observed	Mass error (ppm)
b3	274.1034	274.1058	-9.1	y15	1393.6205	1393.6160	3.2
b4	371.1561	371.1532	7.9	y14	1324.5990	1324.5954	2.8
b5	442.1932	442.1941	-2.0	y13	1211.5150	1211.5159	-0.7
b6	555.2773	555.2763	1.8	y12	1112.4466	1112.4436	2.7
b7	626.3144	626.3106	6.1	y11	1043.4251	1043.4212	3.7
b8	697.3515	697.3494	1.6	y8	804.2981	804.2957	3.0
b9	768.3886	768.3850	4.8				
b10	867.4571	867.4541	3.5				
b11	938.4942	938.4907	3.7				
b17	1538.7519	1538.7515	0.3				
b18	1607.7734	1607.7699	2.1				
b19	1720.8574	1720.8513	3.6				
b20	1819.9259	1819.9224	1.9				

Supplementary Fig. 62. MS and MS^2 fragmentation analysis of the partially dehydrated CP from the co-expression of *kin*A$_{TtoA}$CD in *E. coli* after Gluc-treatment.

a. Schematic process of protease treatment of purified SUMO-His$_6$-KinA$_{TtoA}$(CD), leading the identification of the partially dehydrated CP of KinA$_{TtoA}$. **b**. MS^2 fragmentation spectrum of this peptidyl fragment. **c**. The predicted y- and b- ions with the table of y- and b- ions deduced from MS^2 fragmentation spectrum.

a

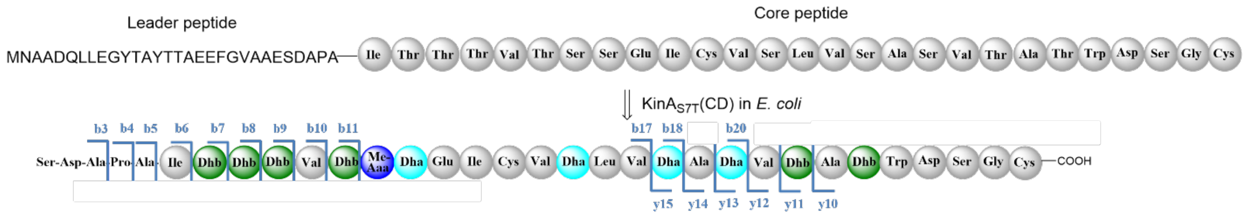

b

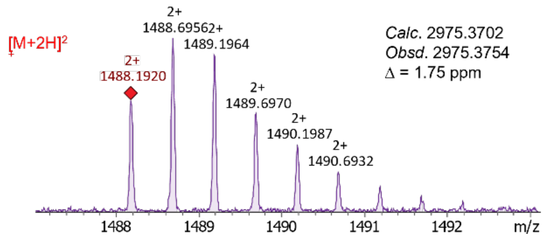

c

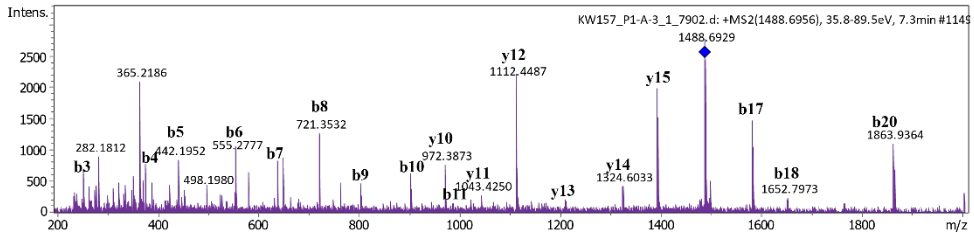

d

b-ions				y-ions			
	Calculated	Observed	Mass error (ppm)		Calculated	Observed	Mass error (ppm)
b3	274.1034	274.1037	-1.3	y15	1393.6205	1393.6211	-0.4
b4	371.1561	371.1562	0.1	y14	1324.5990	1324.6003	-3.2
b5	442.1932	442.1952	-4.3	y13	1211.5150	1211.5117	2.8
b6	555.2773	555.2777	-0.7	y12	1112.4466	1112.4487	-1.9
b7	638.3144	638.3160	-2.4	y11	1043.4251	1043.4250	0.1
b8	721.3515	721.3532	-2.3	y10	972.3880	972.3873	0.8
b9	804.3886	804.3882	0.6				
b10	903.4571	903.4578	-0.8				
b11	986.4942	986.4960	-1.9				
b17	1582.7570	1582.7631	-3.8				
b18	1651.7785	1651.7788	-0.2				
b20	1863.9309	1863.9364	-2.9				

Supplementary Fig. 63. MS and MS^2 fragmentation analysis of the fully dehydrated mutated CP from the co-expression of *kin*A$_{S7T}$CD in *E. coli* after Gluc-treatment.

a. Schematic process of protease treatment of purified SUMO-His$_6$-KinA$_{S7T}$(CD), leading the identification of the partially dehydrated CP of KinA$_{S7T}$. **b**. MS analysis of the partially dehydrated CP of KinA$_{S7T}$. **c**. MS^2 fragmentation spectrum of this peptidyl fragment. **d**. The predicted y- and b-ions with the table of y- and b- ions deduced from MS^2 fragmentation spectrum.

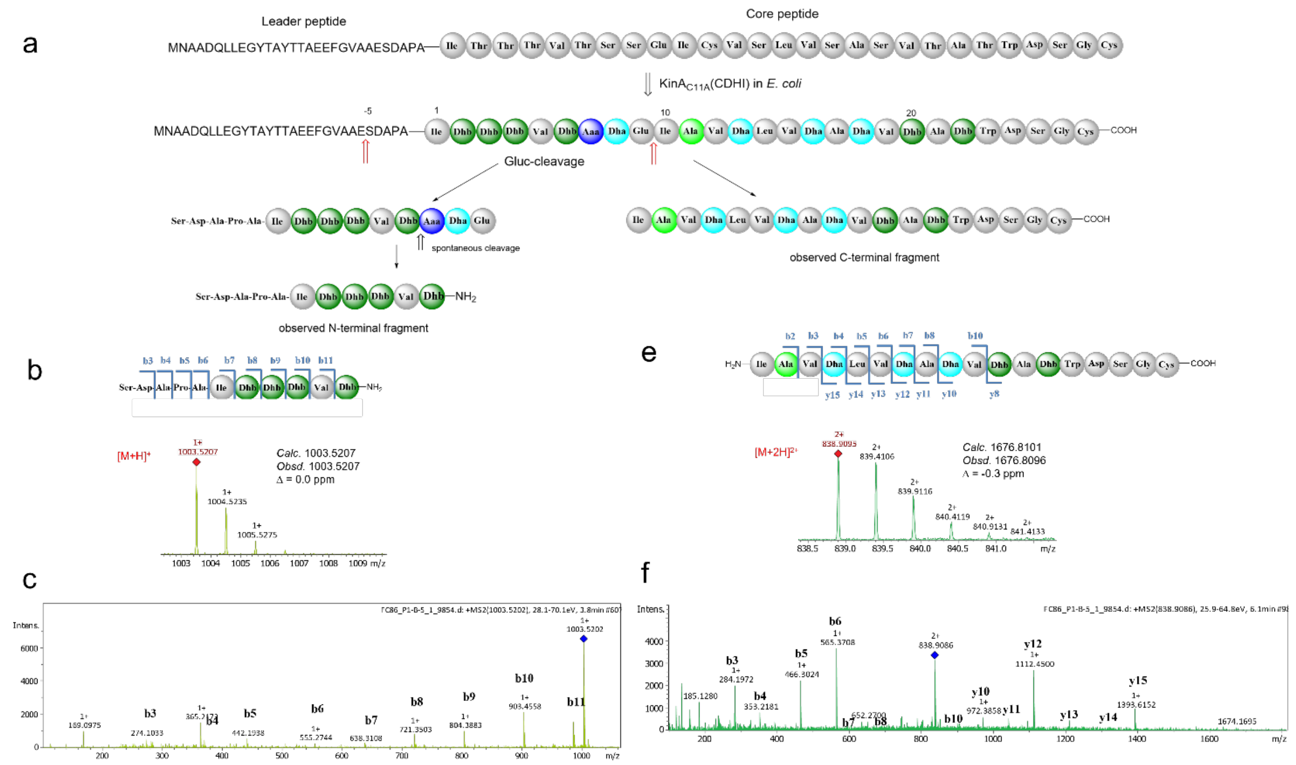

d

b-ions	Calculated	Observed	Mass error (ppm)
b3	274.1034	274.1033	0.3
b4	371.1561	371.1571	-2.5
b5	442.1932	442.1938	-1.4
b6	555.2773	555.2744	5.2
b7	638.3144	638.3108	5.7
b8	721.3515	721.3511	1.7
b9	804.3886	804.3883	0.5
b10	903.4571	903.4558	1.4
b11	986.4942	986.4981	-4.0

g

b-ions	Calculated	Observed	Mass error (ppm)	y-ions	Calculated	Observed	Mass error (ppm)
b2	185.1285	185.1280	2.5	y15	1393.6205	1393.6152	3.8
b3	284.1969	284.1972	-1.1	y14	1324.5990	1324.6046	-5.2
b4	353.2183	353.2181	0.6	y13	1211.5150	1211.5226	-6.3
b5	466.3024	466.3024	0.0	y12	1112.4466	1112.4500	-3.1
b6	565.3708	565.3708	0.0	y11	1043.4251	1043.4208	4.1
b7	634.3923	634.3923	0.0	y10	972.3880	972.3858	2.3
b8	705.4294	705.4312	-2.5	y8	804.2981	804.2967	1.8
b10	873.5153	873.5198	4.5				

Supplementary Fig. 64. MS analysis of trace amount of two dehydrated peptidyl fragments from the co-expression of *kin*A$_{C11A}$CDHI in *E. coli* after Gluc-treatment.

a. Schematic process of protease treatment of purified SUMO-His$_6$-KinA$_{S7T}$(CD), leading the identification of two KinA$_{C11A}$-related dehydrated linear peptidyl fragments. **b**. MS analysis of the N-terminal dehydrated linear CP of KinA$_{C11A}$. **c**. MS^2 fragmentation spectrum of this peptidyl fragment. **d**. The predicted y- and b- ions with the table of y- and b- ions deduced from MS^2 fragmentation spectrum. **e**. MS analysis of the C-terminal dehydrated linear CP of KinA$_{C11A}$. **f**. MS^2 fragmentation spectrum of this peptidyl fragment. **g**. The predicted y- and b- ions with the table of y- and b- ions deduced from MS^2 fragmentation spectrum.

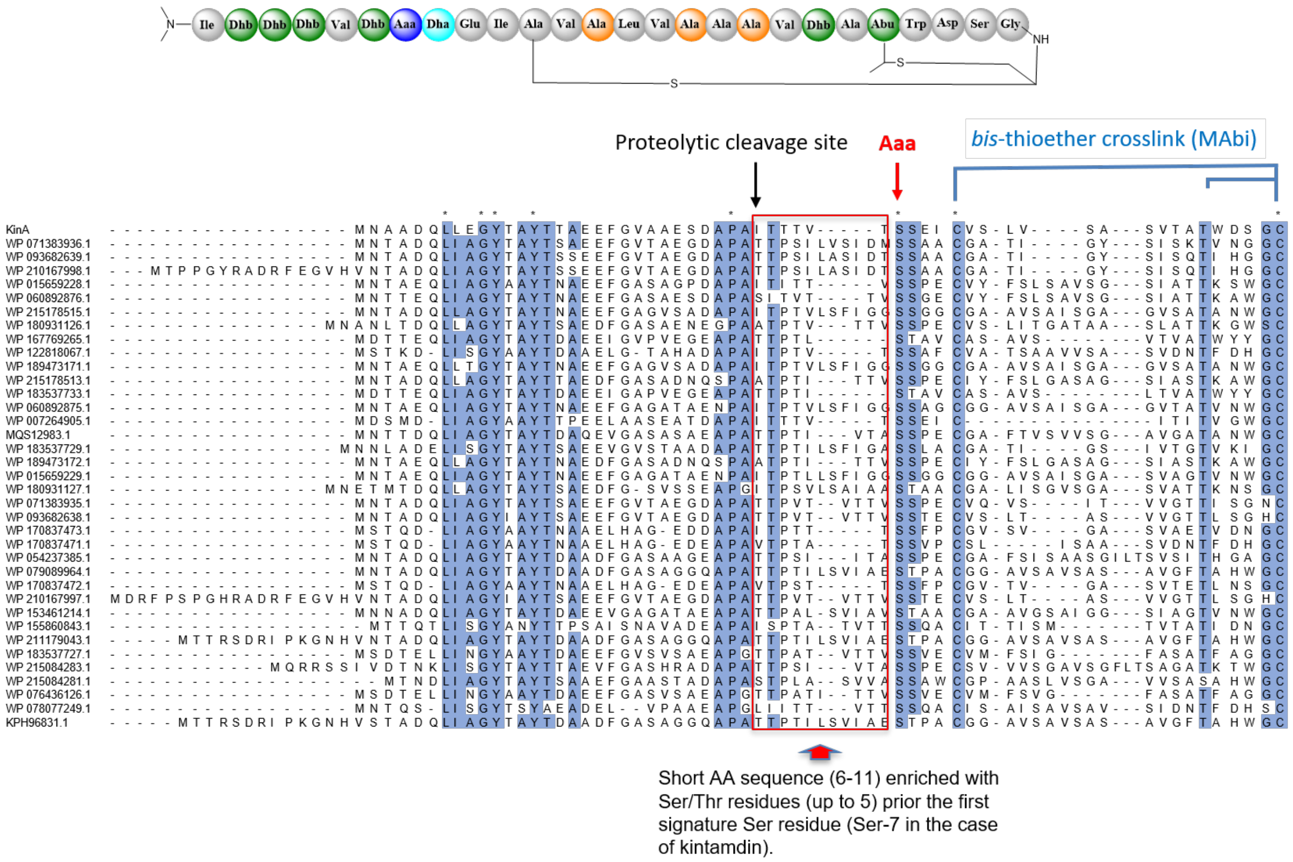

Supplementary Fig. 65. The representative KinA-like peptide sequences identified from conserved genome context.

This suggested that a group of hypothetical peptides contains characteristic motifs, S-S/T-X-X-C-X_n-T-X-X-X-X-C, together with AA sequence enriched with Dha/Dhb residues prior the first signature Ser residue in the mature core peptides.

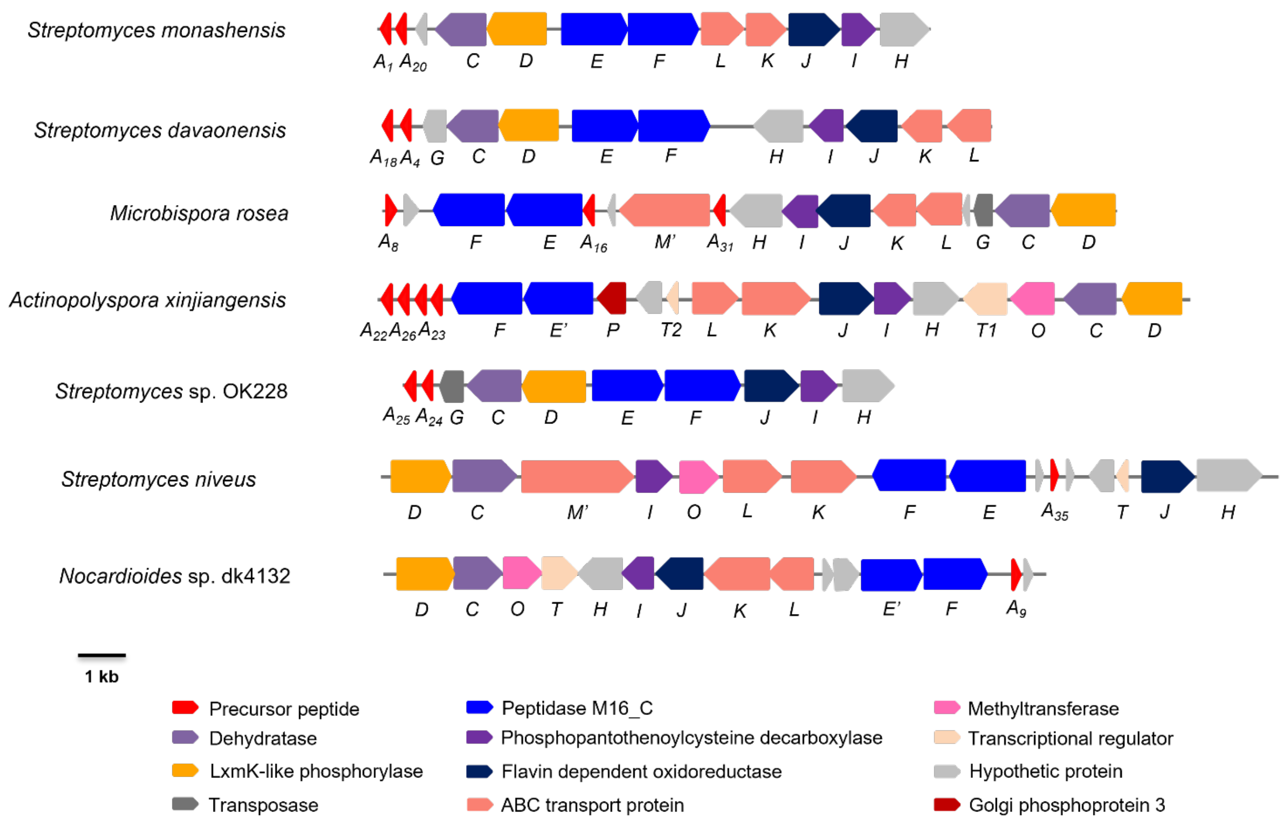

Supplementary Fig. 66. Representative BGCs containing *kinA*-like precursor peptides identified by NCBI BlastP listed in **Supplementary Table S12**.

a

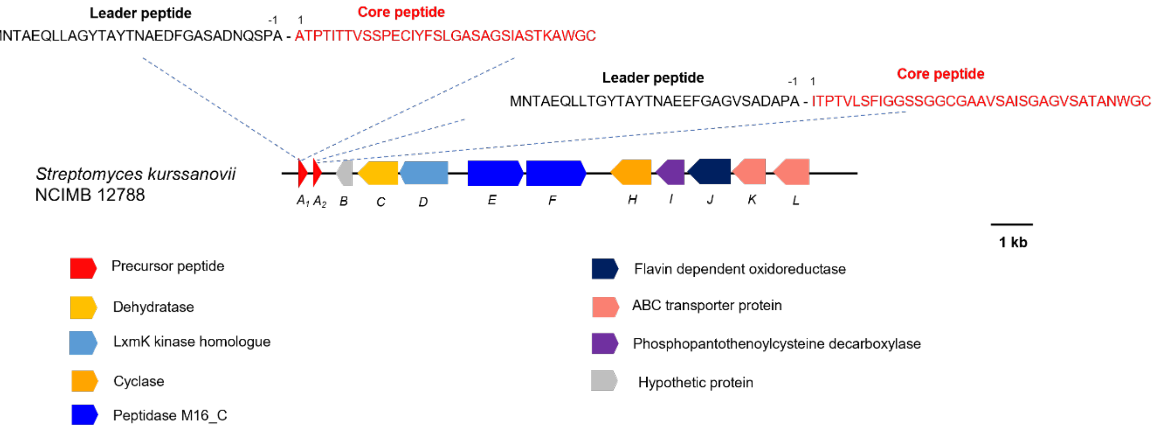

b

homologue	Size (aa)	Accession number#	AA identity	Homologue	Size (aa)	Accession number#	AA identity
KinA-like A_1	66	WP_189473171.1	70%*	KinF-like	470	WP_189473177.1	49%
KinA-like A_2	63	WP_189473172.1	50%*	KinH-like	315	WP_189473180.1	44%
KinC-like	326	WP_189473174.1	55%	KinI-like	204	WP_189473271.1	66%
KinD-like	377	WP_189473175.1	57%	KinJ-like	329	WP_189473181.1	70%
KinE-like	437	WP_189473176.1	62%				

*KinA only share high AA identities of leader peptide with A_1 (70% from 1 to 37) and A_2 (57% from 1 to 44)

accession numbers can be found in the website of National Centre for Biotechnology Information (NCBI).

Supplementary Fig. 67. The BGC responsible for new putative β-bithionin RiPPs in *Streptomyces kurssanovii* NCIMB 12788.

a. The organization of the BGC responsible for new putative β-bithionin RiPPs in *Streptomyces kurssanovii* NCIMB 12788 with two sequences of putative precursor peptides. **b**. ORFs encoded in the BGC with accession numbers.

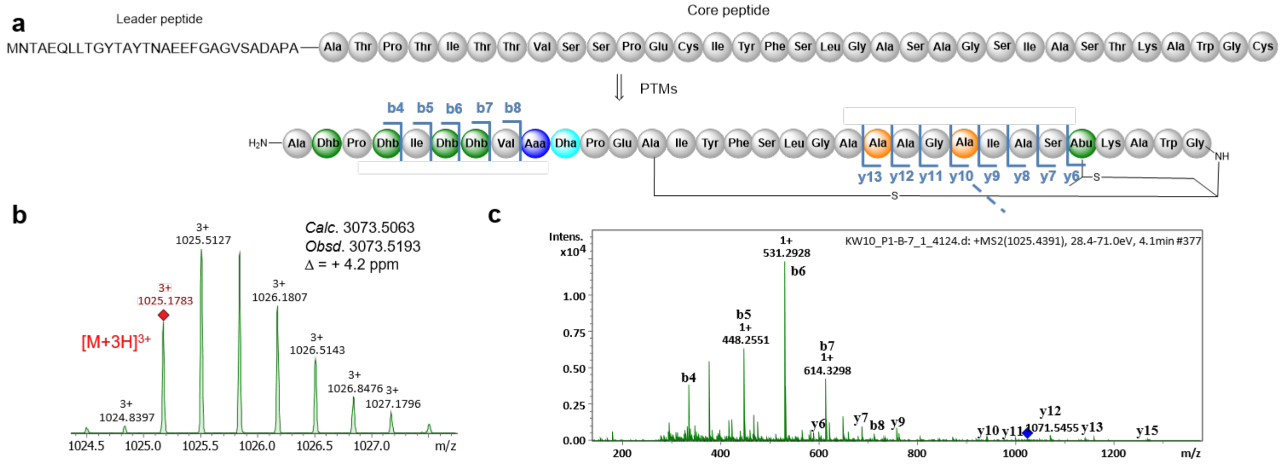

d

	b-ions				y-ions		
	Calculated	Observed	Mass error (ppm)		Calculated	Observed	Mass error (ppm)
b4	335.1714	335.1709	1.4	y15	1270.6367	1270.6579	-
b5	448.2554	448.2551	0.7	y13	1142.5781	1142.5882	-
b6	531.2926	531.2928	-0.4	y12	1071.5404	1071.5455	-4.8
b7	614.3297	614.3298	-0.2	y11	1000.5033	1000.5089	-5.6
b8	713.3981	713.3979	0.2	y10	943.4818	943.4842	-2.5
				y9	872.4447	872.4470	-2.6
				y8	759.3607	759.3605	0.2
				y7	688.3235	688.3227	1.2
				y6	601.2915	601.2903	2.0

Supplementary Fig. 68. Identification of one of the new β-bithionin RiPPs from the culture broth of *Streptomyces kurssanovii* NCIMB 12788 from conserved genomic analysis and experimental evidence.

a. The proposed structure of one of the new β-bithionin RiPPs in *S. kurssanovii* from the precursor peptide KinA-like A_1. **b**. MS spectrum of the corresponding ion ($[M+3H]^{3+}$ = 1025.1783, Δ = 4.2 ppm). **c**. MS^2 fragmentation spectrum of the corresponding ion. **d**. The list of observed y- and b-ions deduced from MS^2 fragmentation analysis.

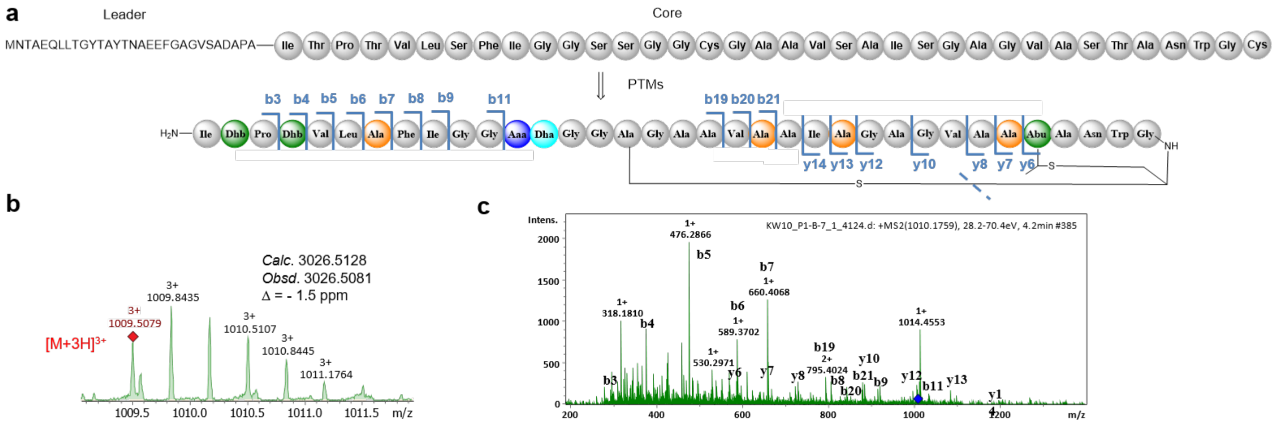

d

	b-ions				y-ions		
	Calculated	Observed	Mass error (ppm)		Calculated	Observed	Mass error (ppm)
b3	294.1812	294.1824	-4.2	y14	1197.5833	1197.5833	0.9
b4	377.2183	377.2183	0.0	y13	1084.4993	1084.5024	-2.9
b5	476.2867	466.2866	0.3	y12	1013.4622	1013.4638	-1.6
b6	589.3708	589.3702	1.0	y10	885.4036	885.4085	-2.5
b7	660.4079	660.4068	1.6	y8	729.3137	729.3148	-1.4
b8	807.4763	807.4794	-3.8	y7	658.2766	658.2762	0.5
b9	920.5604	920.5625	-2.2	y6	587.2395	587.2396	-0.3
b11	1034.6033	1034.6061	-2.7				
b19	1588.7946	795.4024 (2+)	0.5				
b20	1687.8630	844.4376 (2+)	4.8				
b21	1758.9001	880.4586 (2+)	0.8				

Supplementary Fig. 69. Identification of the second new β-bithionin RiPP from the culture broth of *Streptomyces kurssanovii* NCIMB 12788 from conserved genomic analysis and experimental evidence.

a. The proposed structure of the second β-bithionin RiPP in *S. kurssanovii* from the precursor peptide KinA-like A_2. **b**. MS spectrum of the corresponding ion ($[M+3H]^{3+}$ = 1009.5079, Δ = - 1.5 ppm). **c**. MS^2 fragmentation spectrum of the corresponding ion. **d**. The list of observed y- and b-ions deduced from MS^2 fragmentation analysis.

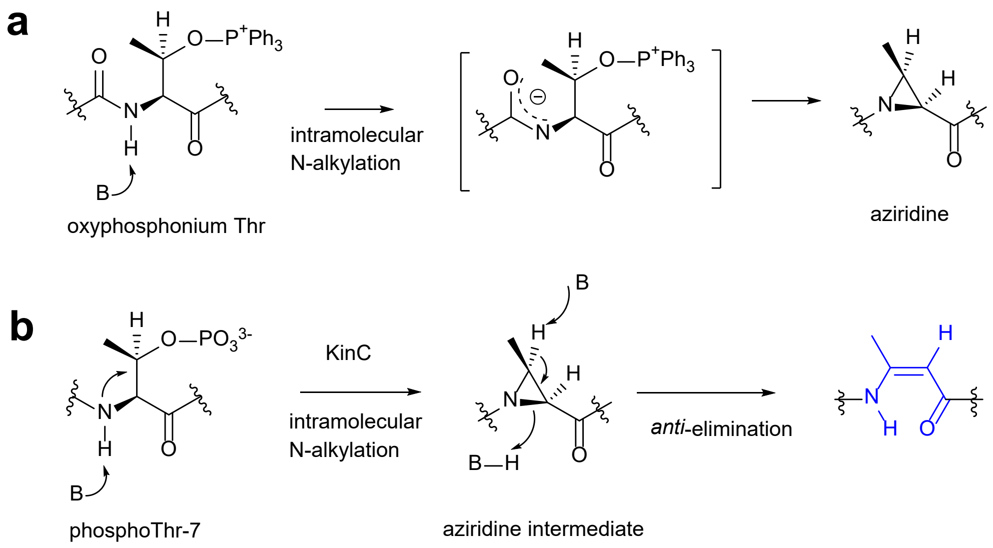

Supplementary Fig. 70. Proposed mechanisms of activation and aziridination on Thr residues in chemical and biochemical reactions.

a. the chemical reaction of activation and aziridination using Mitsunobu agents. It was proposed that deprotonation of the amide-NH by the moderate or strong base (i.e. the reduced DEAD anion (H-DEAD$^-$)) present in the Mitsunobu reaction mixture generates a small amount of amide anion which is expected to promote an intramolecular N-alkylation. **b**. proposed activation and aziridination of Thr residue in the KinA$_{S7T}$ variant, leading to the formation of methyl-Aaa residue.

Supplementary References

1. Wang, M. *et al.* Sharing and community curation of mass spectrometry data with Global Natural Products Social Molecular Networking. *Nat. Biotechnol.* **34**, 828–837 (2016).

2. Zhang, J. J. *et al.* HHS Public Access. *Methods Enzym.* **621**, 87–110 (2019).

3. Hanwell, M. D. *et al.* Avogadro: an advanced semantic chemical editor, visualization, and analysis platform. *J. Cheminform.* **4**, 1–17 (2012).

4. Bannwarth, C. *et al.* GFN2-xTB - An Accurate and Broadly Parametrized Self-Consistent Tight-Binding Quantum Chemical Method with Multipole Electrostatics and Density-Dependent Dispersion Contributions. *J. Chem. Theory Comput.* **15**, 1652–1671 (2019).

5. Caldeweyher, E. *et al.* A generally applicable atomic-charge dependent London dispersion correction. *J. Chem. Phys.* **150**, 154122 (2019).

6. Ryckaert, J.-P. *et al.* Numerical integration of the cartesian equations of motion of a system with constraints: molecular dynamics of n-alkanes. *J. Comput. Phys.* **23**, 327–341 (1977).

7. Van Gunsteren, W. F. & Berendsen, H. J. C. Algorithms for macromolecular dynamics and constraint dynamics. *Mol. Phys.* **34**, 1311–1327 (1977).

8. Gust, B. *et al.* λ Red-mediated genetic manipulation of antibiotic-producing Streptomyces. *Adv. Appl. Microbiol.* **54**, 107–128 (2004).

9. Wagner T, *et al*. Enterococcus faecium produces membrane vesicles containing virulence factors and antimicrobial resistance related proteins. *J Proteomics*. **187**, 28-38 (2018).

10. Tang, X. *et al.* Identification of Thiotetronic Acid Antibiotic Biosynthetic Pathways by Target-directed Genome Mining. *ACS Chem. Biol.* **10**, 2841–2849 (2015).

11. Paranthaman S, Dharmalingam K. Intergeneric conjugation in *Streptomyces peucetius* and *Streptomyces* sp. strain C5: chromosomal integration and expression of recombinant plasmids carrying the chiC gene. *Appl Environ Microbiol*. **69**, 84-91 (2003).

12. MacNeil, D. J. *et al.* Analysis of *Streptomyces avermitilis* genes required for avermectin biosynthesis utilizing a novel integration vector. *Gene* **111**, 61–68 (1992).

13. Datsenko, K. A. & Wanner, B. L. One-step inactivation of chromosomal genes in *Escherichia coli* K-12 using PCR products. *Proc. Natl. Acad. Sci.* **97**, 6640–6645 (2000).

14. Gomez-Escribano, J. P. & Bibb, M. J. Engineering *Streptomyces coelicolor* for heterologous expression of secondary metabolite gene clusters. *Microb. Biotechnol.* **4**, 207–215 (2011).

15 Crisma, M. *et al*. Flat peptides. *J. Am. Chem. Soc*. **121**, 3272–3278 (1999),

16 Santi S., *et al*. Flat, $C^{\alpha,\beta}$-Didehydroalanine Foldamers with Ferrocene Pendants: Assessing the Role of α-Peptide Dipolar Moments. *ChemPlusChem*, **86**, 723-730 (2021).
