# Supplementary material for: A ribosomally synthesised and post-translationally modified peptide containing a β-enamino acid and a macrocyclic motif
Source: Nat Commun. 2022 Aug 26;13:5044. doi: 10.1038/s41467-022-32774-3 (PMC9415263; doi:10.1038/s41467-022-32774-3)
Supplement: Supplementary file 3 — Reporting Summary [file 41467_2022_32774_MOESM3_ESM.pdf]

nature research

Corresponding author(s): HAI DENG

Last updated by author(s): Jul 25, 2022

Reporting Summary

Nature Research wishes to improve the reproducibility of the work that we publish. This form provides structure for consistency and transparency in reporting. For further information on Nature Research policies, see our Editorial Policies and the Editorial Policy Checklist.

Statistics

For all statistical analyses, confirm that the following items are present in the figure legend, table legend, main text, or Methods section.

n/a	Confirmed	
☐	☒	The exact sample size (n) for each experimental group/condition, given as a discrete number and unit of measurement
☐	☒	A statement on whether measurements were taken from distinct samples or whether the same sample was measured repeatedly
☒	☐	The statistical test(s) used AND whether they are one- or two-sided<br>*Only common tests should be described solely by name; describe more complex techniques in the Methods section.*
☒	☐	A description of all covariates tested
☐	☒	A description of any assumptions or corrections, such as tests of normality and adjustment for multiple comparisons
☐	☒	A full description of the statistical parameters including central tendency (e.g. means) or other basic estimates (e.g. regression coefficient) AND variation (e.g. standard deviation) or associated estimates of uncertainty (e.g. confidence intervals)
☒	☐	For null hypothesis testing, the test statistic (e.g. F, t, r) with confidence intervals, effect sizes, degrees of freedom and P value noted<br>*Give P values as exact values whenever suitable.*
☒	☐	For Bayesian analysis, information on the choice of priors and Markov chain Monte Carlo settings
☒	☐	For hierarchical and complex designs, identification of the appropriate level for tests and full reporting of outcomes
☒	☐	Estimates of effect sizes (e.g. Cohen's d, Pearson's r), indicating how they were calculated

Our web collection on statistics for biologists contains articles on many of the points above.

Software and code

Policy information about availability of computer code

Data collection	Bruker Topspin 3; Agilent ChemStation v17; Avogadro 1.2.0, xTB 6.3.0, Biomek software v5.0
Data analysis	Mestrenova 12.0; Bruker DataAnalysis 5.1; Thermo Xcalibur 3.1; ProteoWizard MSconvert; MZmine 2.38; Agilent ChemStation v17; UCSF Chimera 1.15, GraphPad Prism 9.0

For manuscripts utilizing custom algorithms or software that are central to the research but not yet described in published literature, software must be made available to editors and reviewers. We strongly encourage code deposition in a community repository (e.g. GitHub). See the Nature Research guidelines for submitting code & software for further information.

Data

Policy information about availability of data

All manuscripts must include a data availability statement. This statement should provide the following information, where applicable:

- Accession codes, unique identifiers, or web links for publicly available datasets
- A list of figures that have associated raw data
- A description of any restrictions on data availability

Data supporting the findings of this work are available within the paper and its Supplementary Information files. A reporting summary for this Article is available as a Supplementary Information file. The datasets generated and analysed during the current study are available from the corresponding author upon request. GenBank accession numbers used are reported in Supplementary Table 7, 8 and 12.

Field-specific reporting

Please select the one below that is the best fit for your research. If you are not sure, read the appropriate sections before making your selection.

☒ Life sciences ☐ Behavioural & social sciences ☐ Ecological, evolutionary & environmental sciences

For a reference copy of the document with all sections, see nature.com/documents/nr-reporting-summary-flat.pdf

Life sciences study design

All studies must disclose on these points even when the disclosure is negative.

Sample size	No sample size calculation was conducted. three independent measurements were performed for all metabolite analyses in wild type or mutated bacterial strains or gene co-expression of wild type or mutated precursor peptides with various biosynthetic genes following standards in the field.
Data exclusions	No data were excluded
Replication	triplicated HPLC and LC-MS analysis for metabolite production evaluation from wild type/variant bacterial strains and in gene co-expression systems of wild-type and mutated precursor peptides with various biosynthetic genes; PCR verification followed by DNA sequencing to confirm the mutagenesis.
Randomization	this is not relevant since the study does not involve different experimental groups
Blinding	this is not relevant since the study does not involve clinical trial and group allocation

Reporting for specific materials, systems and methods

We require information from authors about some types of materials, experimental systems and methods used in many studies. Here, indicate whether each material, system or method listed is relevant to your study. If you are not sure if a list item applies to your research, read the appropriate section before selecting a response.

Materials & experimental systems

n/a	Involved in the study	
☒	☐	Antibodies
☐	☒	Eukaryotic cell lines
☒	☐	Palaeontology and archaeology
☒	☐	Animals and other organisms
☒	☐	Human research participants
☒	☐	Clinical data
☒	☐	Dual use research of concern

Methods

n/a	Involved in the study	
☒	☐	ChIP-seq
☒	☐	Flow cytometry
☒	☐	MRI-based neuroimaging

Eukaryotic cell lines

Policy information about cell lines

Cell line source(s)	Lung normal cell lines (ATCC CCL-171); Skin cancer cell ATCC CRL-11147; Breast cancer cell ATCC HTB-22; Colon cancer cell ATCC HTB-38
Authentication	none of the cell lines were authenticated as they are commercially available.
Mycoplasma contamination	Not tested for mycoplasma contamination
Commonly misidentified lines (See ICLAC register)	no commonly misidentified cell lines.
